# Health workers’ perceptions and experiences of using mHealth technologies to deliver primary healthcare services: a qualitative evidence synthesis

**DOI:** 10.1002/14651858.CD011942.pub2

**Published:** 2020-03-26

**Authors:** Willem A Odendaal, Jocelyn Anstey Watkins, Natalie Leon, Jane Goudge, Frances Griffiths, Mark Tomlinson, Karen Daniels

**Affiliations:** South African Medical Research CouncilHealth Systems Research UnitCape TownWestern CapeSouth Africa; Stellenbosch UniversityDepartment of PsychiatryCape TownSouth Africa; University of WarwickWarwick Medical SchoolCoventryUK; Brown UniversitySchool of Public HealthProvidenceRhode IslandUSA; University of the WitwatersrandCentre for Health Policy, School of Public Health, Faculty of Health SciencesJohannesburgSouth Africa; Stellenbosch UniversityInstitute for Life Course Health Research, Department of Global HealthCape TownSouth Africa; Queens UniversitySchool of Nursing and MidwiferyBelfastUK; University of Cape TownHealth Policy and Systems Division, School of Public Health and Family MedicineCape TownWestern CapeSouth Africa7925

## Abstract

**Background:**

Mobile health (mHealth), refers to healthcare practices supported by mobile devices, such as mobile phones and tablets. Within primary care, health workers often use mobile devices to register clients, track their health, and make decisions about care, as well as to communicate with clients and other health workers. An understanding of how health workers relate to, and experience mHealth, can help in its implementation.

**Objectives:**

To synthesise qualitative research evidence on health workers' perceptions and experiences of using mHealth technologies to deliver primary healthcare services, and to develop hypotheses about why some technologies are more effective than others.

**Search methods:**

We searched MEDLINE, Embase, CINAHL, Science Citation Index and Social Sciences Citation Index in January 2018. We searched Global Health in December 2015. We screened the reference lists of included studies and key references and searched seven sources for grey literature (16 February to 5 March 2018). We re‐ran the search strategies in February 2020. We screened these records and any studies that we identified as potentially relevant are awaiting classification.

**Selection criteria:**

We included studies that used qualitative data collection and analysis methods. We included studies of mHealth programmes that were part of primary healthcare services. These services could be implemented in public or private primary healthcare facilities, community and workplace, or the homes of clients. We included all categories of health workers, as well as those persons who supported the delivery and management of the mHealth programmes. We excluded participants identified as technical staff who developed and maintained the mHealth technology, without otherwise being involved in the programme delivery. We included studies conducted in any country.

**Data collection and analysis:**

We assessed abstracts, titles and full‐text papers according to the inclusion criteria. We found 53 studies that met the inclusion criteria and sampled 43 of these for our analysis. For the 43 sampled studies, we extracted information, such as country, health worker category, and the mHealth technology. We used a thematic analysis process. We used GRADE‐CERQual to assess our confidence in the findings.

**Main results:**

Most of the 43 included sample studies were from low‐ or middle‐income countries. In many of the studies, the mobile devices had decision support software loaded onto them, which showed the steps the health workers had to follow when they provided health care. Other uses included in‐person and/or text message communication, and recording clients' health information. Almost half of the studies looked at health workers' use of mobile devices for mother, child, and newborn health.

We have moderate or high confidence in the following findings.

***mHealth changed how health workers worked with each other***: health workers appreciated being more connected to colleagues, and thought that this improved co‐ordination and quality of care. However, some described problems when senior colleagues did not respond or responded in anger. Some preferred face‐to‐face connection with colleagues. Some believed that mHealth improved their reporting, while others compared it to "big brother watching".

***mHealth changed how health workers delivered care***: health workers appreciated how mHealth let them take on new tasks, work flexibly, and reach clients in difficult‐to‐reach areas. They appreciated mHealth when it improved feedback, speed and workflow, but not when it was slow or time consuming. Some health workers found decision support software useful; others thought it threatened their clinical skills. Most health workers saw mHealth as better than paper, but some preferred paper. Some health workers saw mHealth as creating more work.

***mHealth led to new forms of engagement and relationships with clients and communities***: health workers felt that communicating with clients by mobile phone improved care and their relationships with clients, but felt that some clients needed face‐to‐face contact. Health workers were aware of the importance of protecting confidential client information when using mobile devices. Some health workers did not mind being contacted by clients outside working hours, while others wanted boundaries. Health workers described how some community members trusted health workers that used mHealth while others were sceptical. Health workers pointed to problems when clients needed to own their own phones.

***Health workers' use and perceptions of mHealth could be influenced by factors tied to costs, the health worker, the technology, the health system and society, poor network access, and poor access to electricity***: some health workers did not mind covering extra costs. Others complained that phone credit was not delivered on time. Health workers who were accustomed to using mobile phones were sometimes more positive towards mHealth. Others with less experience, were sometimes embarrassed about making mistakes in front of clients or worried about job security. Health workers wanted training, technical support, user‐friendly devices, and systems that were integrated into existing electronic health systems. The main challenges health workers experienced were poor network connections, access to electricity, and the cost of recharging phones. Other problems included damaged phones. Factors outside the health system also influenced how health workers experienced mHealth, including language, gender, and poverty issues. Health workers felt that their commitment to clients helped them cope with these challenges.

**Authors' conclusions:**

Our findings propose a nuanced view about mHealth programmes. The complexities of healthcare delivery and human interactions defy simplistic conclusions on how health workers will perceive and experience their use of mHealth. Perceptions reflect the interplay between the technology, contexts, and human attributes. Detailed descriptions of the programme, implementation processes and contexts, alongside effectiveness studies, will help to unravel this interplay to formulate hypotheses regarding the effectiveness of mHealth.

## Plain language summary

**Health workers' perceptions and experiences of using mHealth technologies to deliver primary healthcare services: a qualitative evidence synthesis**

**What is the aim of this review?**

The aim of this Cochrane Review of qualitative research was to explore how health workers view and experience the use of mobile phones and tablets when delivering primary healthcare services. To answer this, we analysed 43 studies about health workers' views and experiences of mobile health (mHealth) programmes.

**Key messages**

Health workers welcomed the benefits of mHealth, and described how they used mobile phones to improve their work and relationships with each other and with clients. However, they also experienced challenges, including poor network coverage and access to electricity. People implementing mHealth programmes should try to address these challenges and build on health workers' positive experiences.

**What was studied in this review?**

Health workers often use mobile phones and tablets to provide services. It is important to understand what health workers think about these devices, as this can affect the quality of services. We looked for studies that explored health workers' views and experiences of using mobile devices in primary care.

**What are the main findings of this review?**

We sampled 43 of the 53 included studies in this review. Most were from low‐ or middle‐income countries, and included both lay health workers and health professionals. Many used mobile phones or tablets when delivering health care to mothers, children or newborns. Many of the mobile devices had software that helped health workers follow the correct steps when providing services. Health workers also used mobile phones to communicate with clients, and to record clients' health information.

We have moderate or high confidence in the following findings.

***mHealth changed how health workers worked with each other***: health workers appreciated being more connected to colleagues, and thought that this improved co‐ordination and quality of care. However, some described problems when senior colleagues did not respond or responded in anger. Some preferred face‐to‐face connection with colleagues. Some believed that mHealth improved their reporting, while others compared it to "big brother watching".

***mHealth changed how health workers delivered care***: health workers appreciated how mHealth let them take on new tasks, work flexibly, and reach clients in difficult‐to‐reach areas. They appreciated mHealth when it improved feedback, speed and workflow, but not when it was slow or time consuming. Some health workers found decision support software useful; others thought it threatened their clinical skills. Most health workers saw mHealth as better than paper, but others saw mHealth as creating more work.

***mHealth led to new forms of engagement and relationships with clients and communities***: health workers felt that communicating with clients by mobile phone improved care and their relationships with clients, but felt that some clients needed face‐to‐face contact. Health workers were aware of the importance of protecting confidential client information when using mobile devices. Some health workers did not mind being contacted by clients outside working hours, while others wanted boundaries. Health workers described how some community members trusted health workers that used mHealth while others were sceptical. Health workers pointed to problems when clients needed to own their own phones.

***Health workers' use and perceptions of mHealth could be influenced by factors tied to costs, the health worker, the technology, the health system and society, poor network access, and poor access to electricity***: some health workers did not mind covering extra costs. Others complained that phone credit was not delivered on time. Health workers who were accustomed to using mobile phones were sometimes more positive towards mHealth. Others with less experience, were sometimes embarrassed about making mistakes in front of clients or worried about job security. Health workers wanted training, technical support, user‐friendly devices, and systems that were integrated into existing electronic health systems. The main challenges health workers experienced were poor network connections, access to electricity, and the cost of recharging phones. Other problems included damaged phones. Factors outside the health system also influenced how health workers experienced mHealth, including language, gender, and poverty issues. Health workers felt that their commitment to clients helped them cope with these challenges.

**How up‐to‐date is this review?**

We searched for studies published up to January 2018.

## Summary of findings

**Summary of findings for the main comparison:** Summary of qualitative findings for the main comparison

**Finding**	**Studies contributing to the review finding**	**GRADE‐CERQual assessment of confidence in the** **evidence**	**Explanation of GRADE‐CERQual assessment**
**mHealth changed how health workers worked with each other **			
1. Through being connected to other health workers and across various healthcare services, health workers appreciated that mobile devices allowed them to better co‐ordinate the delivery of care.	Barnabee 2014; Chang 2011; Hampshire 2016; Henry 2016; Huq 2014; Khan 2015; Lodhia 2016; Madon 2014; Messinger 2017; Murray 2011; Mwendwa 2016; Quinn 2013; Ramirez 2017; Rothstein 2016; Schoen 2017; Toda 2017; van der Wal 2016; Watkins 2018	Moderate confidence	Due to no/very minor concerns regarding coherence, minor concerns regarding adequacy and methodological limitations, and moderate concerns regarding relevance
2. Lower‐level health workers valued being able to reach higher‐level health workers via mobile devices, and perceived the advice and support they received as improving their care and as satisfying to clients. When higher‐level professionals responded in anger, it made lower‐level health workers reluctant to call them.	Ayiasi 2015; Chang 2011; Cherrington 2015; Hampshire 2016; Huq 2014; Khan 2015; Lodhia 2016; Madon 2014; Messinger 2017; Mwendwa 2016; Quinn 2013; Toda 2017; van der Wal 2016; Watkins 2018	Moderate confidence	Due to no/very minor concerns regarding coherence, relevance, and adequacy, but moderate concerns regarding methodological limitations
3. When higher‐level health workers failed to respond and support lower‐level workers through mobile devices, lower‐level staff had negative perceptions of these devices. One study emphasised the importance of having health professionals' buy‐in with mobile health to ensure that mobile devices were optimally used to support lay health workers.	Cherrington 2015; Huq 2014; Mwendwa 2016; Quinn 2013; Toda 2017; van der Wal 2016	Moderate confidence	Due to no/very minor concerns regarding coherence and relevance, minor concerns regarding methodological limitations, but moderate concerns regarding adequacy
4. The use of mobile devices allowed some health workers to feel connected to their peers within their own organisations. However, others preferred face‐to‐face communication with their peers.	Barnabee 2014; Hampshire 2016; Henry 2016; Jennings 2013; Madon 2014; Valaitis 2005; van der Wal 2016; Watkins 2018	Moderate confidence	Due to no/very minor concerns regarding coherence, minor concerns regarding methodological limitations and relevance, and moderate concerns regarding adequacy
5. Some health workers relayed that mobile devices improved their reporting to supervisors and encouraged them to report more truthfully. Others compared mobile device‐facilitated supervision to "big brother watching". Some supervisors thought that mobile devices allowed them to better identify staff who needed support.	Barnabee 2014; Chang 2011; Jennings 2013; Madon 2014; Medhanyie 2015; Mwendwa 2016; Schoen 2017; Toda 2017; Valaitis 2005; van der Wal 2016	Moderate confidence	Due to no/very minor concerns regarding coherence, minor concerns regarding relevance and adequacy, and moderate concerns regarding methodological limitations
6. Health workers had positive experiences with using instant messaging through WhatsApp. This application was seen as cheap and suitable for a range of activities, such as communicating with peers and posting photos as evidence of work done.	Hampshire 2016; Henry 2016; Schoen 2017	Very low confidence	Due to serious concerns regarding methodological limitations and adequacy, moderate concerns regarding relevance, and no/very minor concerns regarding coherence
7. Even when health workers received messages that were automated, rather than sent directly from a manager or supervisor, this was still experienced and responded to, as a kind of supervision. Some lower‐level health workers experienced it as supportive to their work, while others felt guilty for not providing correct care as per these messages.	Cherrington 2015; Ilozumba 2018; Jones 2012; Mwendwa 2016	Low confidence	Due to moderate concerns regarding relevance and adequacy, minor concerns regarding methodological limitations, and no/very minor concerns regarding coherence
**mHealth changed how health workers delivered care**			
8. The task optimisation enabled through mHealth interventions was widely valued by health workers.	Barnabee 2014; Chang 2011; Ilozumba 2018; Khan 2015; Kolltveit 2017; Lodhia 2016; Praveen 2014	Moderate confidence	Due to no/very minor concerns regarding coherence, relevance, and adequacy, and moderate concerns regarding methodological limitations
9. At times, health workers used their mobile devices to access the Internet for health information, and found it useful when they were with clients who needed the information. This interaction also included health workers providing clients with additional information beyond the healthcare intervention. But, if the only way that health workers could access online information, required them to use their own money to purchase data, then this could be prohibitive to them accessing such information.	Bacchus 2016; Hampshire 2016; Schoen 2017; Watkins 2018	Low confidence	Due to no/very minor concerns regarding coherence, minor concerns regarding methodological limitations and relevance, and serious concerns regarding adequacy
10. mHealth held the promise of increasing service efficiency for many health workers, but the experience of whether this promise was borne out in practice, varied in the accounts of health workers. It was experienced as efficient if it improved feedback, speed and workflow, but inefficient when the technology was slow and time consuming. Some were concerned that if mHealth was too efficient, making work faster, that this may justify staff cutbacks.	Ayiasi 2015; Barnabee 2014; Chang 2011; Cherrington 2015; Coetzee 2017; Garg 2016; Ginsburg 2016; Hampshire 2016; Hao 2015; Huq 2014; Jennings 2013; Jones 2012; Kolltveit 2017; Lodhia 2016; Madon 2014; Medhanyie 2015; Messinger 2017; Mwendwa 2016; Praveen 2014; Ramirez 2017; Rothstein 2016; Schoen 2017Schoen 2017; Toda 2017; Valaitis 2005; van der Wal 2016; Watkins 2018	High confidence	Due to no/very minor concerns regarding relevance and adequacy, and minor concerns regarding methodological limitations and coherence
11. Health workers frequently reported mobile devices as overcoming the difficulties of rural and geographically challenging contexts when it made it possible for them to provide health care without having to travel. Some reported that reducing travel time allowed them more time with their clients.	Chang 2011; Hampshire 2016; Hirsch‐Moverman 2017; Lodhia 2016; Messinger 2017; Mwendwa 2016; Quinn 2013; Rothstein 2016; Toda 2017; Valaitis 2005	High confidence	Due to no/very minor concerns regarding coherence and adequacy, and minor concerns regarding methodological limitations and relevance
12. Health workers appreciated the portability and work schedule flexibility of mobile devices.	Hampshire 2016; Murray 2011; Nguyen 2015; Orchard 2014; Ramirez 2017; Schoen 2017; Toda 2017; Valaitis 2005; van der Wal 2016	Moderate confidence	Due to no/very minor concerns regarding coherence, relevance, and adequacy, but moderate concerns regarding methodological limitations
13. Through mHealth, health workers were able to use treatment and screening algorithms that were loaded onto mobile devices. Their perceptions of using these electronic algorithms ranged from finding it easy and useful, to threatening their clinical competency, and an information overload. There were also some concerns that erroneous data entry may lead to wrong treatment guidance.	Ginsburg 2016; Ilozumba 2018; Lodhia 2016; Mitchell 2012; Mwendwa 2016; Nguyen 2015; Orchard 2014; Ramirez 2017; Rothstein 2016; Shao 2015; Surka 2014; Tewari 2017; van der Wal 2016	High confidence	Due to no/very minor concerns regarding coherence, relevance, and adequacy, and minor concerns regarding methodological limitations
14. Using mobile devices to record routine client or surveillance data was mostly perceived by health workers and their managers as helpful for decision making, and increasing community and health worker appreciation of these data.	Khan 2015; Lodhia 2016; Madon 2014; Murray 2011; Nguyen 2015; Ramirez 2017; Rothstein 2016; Schoen 2017; Toda 2017	Moderate confidence	Due to no/very minor concerns regarding coherence, relevance, and adequacy, but moderate concerns regarding methodological limitations
15. In most cases health workers perceived mobile health as more advantageous than paper. However, some continued to prefer paper.	Bacchus 2016; Coetzee 2017; Ginsburg 2016; Madon 2014; Mitchell 2012; Mwendwa 2016; Nguyen 2015; Rothstein 2016; Schoen 2017; Surka 2014; Toda 2017; Valaitis 2005; van der Wal 2016; Vedanthan 2015; Watkins 2018	High confidence	Due to no/very minor concerns regarding coherence, relevance, and adequacy, and minor concerns regarding methodological limitations
16. mHealth interventions sometimes required health workers to perform tasks that were peripheral to regular service delivery, such as registering clients onto the system. These more menial tasks were sometimes regarded as undermining to professional staff.	Hirsch‐Moverman 2017; Medhanyie 2015; Murray 2015; Wolff‐Piggott 2018	Very low confidence	Due to serious concerns regarding methodological limitations and adequacy, and moderate concerns regarding coherence and relevance
17. Some health workers experienced the use of mHealth as generating an extra workload when, for instance, it resulted in reaching more clients needing care, or having to maintain both a mobile health and paper system. Some workers disliked this, particularly when their superiors did not perceive their mobile health work as part of their job description. Others did not object to the additional work, yet others wanted to be remunerated.	Chang 2011; Hao 2015; Kolltveit 2017; Lodhia 2016; Murray 2015; Mwendwa 2016; Praveen 2014; Rothstein 2016; Shao 2015; Wolff‐Piggott 2018	High confidence	Due to no/very minor concerns regarding coherence, relevance and adequacy, and minor concerns regarding methodological limitations
**mHealth led to new forms of engagement and relationships with clients and communities**			
18. Through mobile devices, health workers and clients could communicate directly with each other, which health workers reported as improving care and their relationship with clients. When clients initiated the contact, health workers felt that clients took ownership of their health. Health workers felt that some clients still warrant face‐to‐face contact.	Barnabee 2014; Chang 2011; Cherrington 2015; Garg 2016; Hirsch‐Moverman 2017; Huq 2014; Jennings 2013; Lodhia 2016; Messinger 2017; Schoen 2017; van der Wal 2016; Watkins 2018	Moderate confidence	Due to no/very minor concerns regarding coherence and relevance, minor concerns regarding methodological limitations, and moderate concerns regarding adequacy
19. Health workers were aware of the importance of protecting confidential client information when using mobile devices, and the confidentiality risks in cases of stolen phones and using their SIM cards in colleagues' phones. Health workers were alert to clients' concerns when they shared personal information concerning stigmatised issues, such as HIV/AIDS and interpersonal violence, and suggested ways to keep the information confidential. They emphasised building a trusting relationship with clients prior to using the devices.	Bacchus 2016; Coetzee 2017; Garg 2016; Hirsch‐Moverman 2017; Lodhia 2016; Murray 2015; Mwendwa 2016; Rothstein 2016; Valaitis 2005; Wolff‐Piggott 2018	High confidence	Due to no/minor concerns regarding methodological limitations, coherence, relevance, and adequacy
20. Health workers were concerned that concentrating too much on the mobile technology during client consultations could be to the detriment of their service and interaction with clients.	Bacchus 2016; Schoen 2017; Vedanthan 2015	Low confidence	Due to serious concerns about adequacy, moderate concerns regarding relevance, minor concerns regarding methodological limitations, and no/very minor concerns regarding coherence
21. Health workers had differing reactions to being contactable via mobile devices during and outside of working hours: some felt it was useful, some were ambivalent about it, and others objected to it. Workers suggested setting boundaries to protect themselves from this.	Chang 2011; Cherrington 2015; Hampshire 2016; Huq 2014; Jennings 2013; Schoen 2017; Valaitis 2005	Moderate confidence	Due to no/very minor concerns regarding methodological limitations and coherence, minor concerns regarding relevance, and moderate concerns regarding adequacy
22. Health workers experienced the use of mobile technology to provide health care, as being met with both trust and skepticism from clients and the communities they served. They described how trust or skepticism in the device was translated into trust or skepticism of their service when using the device. Some found that using mobile devices raised their social status with clients, and even their families. Others were concerned that using expensive equipment would emphasise inequity between themselves and clients.	Ayiasi 2015; Barnabee 2014; Cherrington 2015; Coetzee 2017; Ginsburg 2016; Ilozumba 2018; Jones 2012; Khan 2015; Lodhia 2016; Madon 2014; Mitchell 2012; Mwendwa 2016; Valaitis 2005; van der Wal 2016	High confidence	Due to no/very minor concerns regarding coherence, relevance, and adequacy, and minor concerns regarding methodological limitations
23. Health workers experienced clients as having an opinion not only about their use of mobile devices, but as having an opinion on the devices themselves, which influenced how they responded to care delivered with the support of these devices. Health workers ascribed clients' enthusiasm for mobile devices as due to these clients' perception of the devices as prestigious, offering trustworthy information, and providing confidentiality. They perceived clients as more receptive when these clients were familiar with the devices used. There were concerns that clients who felt that the use of these devices during care was too time consuming, and would respond negatively to its use.	Bacchus 2016; Garg 2016; Ginsburg 2016; Ilozumba 2018; Jones 2012; Khan 2015; Messinger 2017; Mitchell 2012; Schoen 2017; Shao 2015; Valaitis 2005; van der Wal 2016; Vedanthan 2015; Westergaard 2017	Moderate confidence	Due to no/very minor concerns regarding coherence, relevance and adequacy, but moderate concerns regarding methodological limitations
24. Some interventions required clients to have phones as well as health workers. Health workers described this as challenging for multiple reasons, including clients not having phones, changing their phone numbers regularly, not knowing how to use a phone, being a target of crime because of possession of the phone, and women being prohibited from accessing phones. Health workers suggested competitive pricing to increase clients' access to phones, and to issue clients with phones.	Chang 2011; Hirsch‐Moverman 2017; Huq 2014; Murray 2015; Tewari 2017; van der Wal 2016; Wolff‐Piggott 2018	Moderate confidence	Due to no/very minor concerns regarding coherence, minor concerns regarding methodological limitations and adequacy, and moderate concerns regarding relevance
25. Health workers were ambivalent about interventions that required clients to use the health workers' mobile devices during consultations. Their optimism was tempered by concern that there was a loss of meaningful engagement with clients.	Bacchus 2016; Coetzee 2017	Low confidence	Due to serious concerns regarding adequacy, moderate concerns regarding relevance, and no/very minor concerns regarding methodological limitations and coherence
26. Health workers reported that their access to mobile devices was beneficial to clients and communities who were too poor to own mobile phones.	Chang 2011; van der Wal 2016	Very low confidence	Due to serious concerns regarding relevance and adequacy, moderate concerns regarding methodological limitations, and no/very minor concerns regarding coherence
27. Health workers felt that health promotion and educational messaging directed at clients using mobile health interventions, impacted positively on clients' health behaviours, but cautioned against repetitive showing of health promotion videos. In one instance, issuing clients with mobile phones led to increased use of healthcare services.	Bacchus 2016; Barnabee 2014; Chang 2011; Coetzee 2017; Ginsburg 2016; Huq 2014; Ilozumba 2018; Jones 2012; Lodhia 2016; Madon 2014; Murray 2011; Praveen 2014; van der Wal 2016	Moderate confidence	Due to no/very minor concerns regarding methodological limitations, coherence, and relevance, but moderate concerns regarding adequacy
**Health workers' use and perceptions of mHealth could be influenced by factors tied to costs, the health worker, the technology, the health system and society, poor network access, and poor access to electricity **			
28. Some health workers accepted bearing the costs of mHealth interventions themselves, but were dissatisfied when phone credit to use the phones was not delivered on time. Health workers felt that clients appreciated it when health workers called them, as it saved them costs.	Hampshire 2016; Khan 2015; Messinger 2017; Quinn 2013; van der Wal 2016; Watkins 2018; Wolff‐Piggott 2018	High confidence	Due to no/very minor concerns regarding coherence, relevance and adequacy, and minor concerns regarding methodological limitations
29. Health workers' digital literacy impacted on their experience and perceptions of the use of mobile devices in health service delivery: being digitally literate resulted in positive experiences and perceptions, whilst low digital literacy caused concerns about job security and embarrassment when making mistakes in front of clients. For some workers, prior exposure to mobile devices did not affect their perceptions and use of mobile health. Some turned their lack of digital literacy into building a relationship with clients by asking clients to show them how to use the devices. Not using the devices often enough, resulted in loss in digital literacy.	Bacchus 2016; Cherrington 2015; Coetzee 2017; Ginsburg 2016; Hao 2015; Hirsch‐Moverman 2017; Ilozumba 2018; Kolltveit 2017; Madon 2014; Mitchell 2012; Murray 2011; Mwendwa 2016; Nguyen 2015; Praveen 2014; Quinn 2013; Shao 2015; Surka 2014; Valaitis 2005; van der Wal 2016; Watkins 2018	Moderate confidence	Due to no/very minor concerns regarding coherence, relevance, and adequacy, but moderate concerns regarding methodological limitations
30. Health workers expressed a need for training and familiarity with mobile devices to overcome their initial anxiety in using the devices. Peer training from technologically proficient colleagues was experienced as valuable. In several cases, health workers wanted refresher training and pointed to the importance of training replacement staff. Not having mentors who used mobile devices, impacted negatively on lower‐level workers' ability to learn how to use these devices.	Coetzee 2017; Ginsburg 2016; Ilozumba 2018; Kolltveit 2017; Lodhia 2016; Madon 2014; Murray 2011; Mwendwa 2016; Nguyen 2015; Praveen 2014; Rothstein 2016; Tewari 2017; Toda 2017; van der Wal 2016; Vedanthan 2015	High confidence	Due to no/very minor concerns regarding coherence, relevance, and adequacy, and minor concerns regarding methodological limitations
31. All categories of health workers required technical support to solve user problems. At times, face‐to‐face support was provided, but technical support from proficient colleagues was useful too. Having technical problems solved through real‐time improvements worked well for some health workers, while others suggested a help function be added to the devices.	Cherrington 2015; Garg 2016; Hao 2015; Ilozumba 2018; Kolltveit 2017; Lodhia 2016; Madon 2014; Murray 2011; Mwendwa 2016; Rothstein 2016; Toda 2017; van der Wal 2016	High confidence	Due to no/very minor concerns regarding coherence, relevance, and adequacy, and minor concerns regarding methodological limitations
32. Health workers highlighted that mobile technology applications should be user‐friendly, easy to learn, and improve the quality of their care. When the applications were not easy to use, health workers became frustrated and reluctant users of mobile devices.	Ginsburg 2016; Khan 2015; Kolltveit 2017; Lodhia 2016; Mwendwa 2016; Praveen 2014; Ramirez 2017; Rothstein 2016; Schoen 2017; Toda 2017; van der Wal 2016	High confidence	Due to no/very minor concerns regarding coherence, relevance, and adequacy, and minor concerns regarding methodological limitations
33. Health workers held mixed views on choosing between tablets and smartphones. Some felt that the type of content on the device was more important than the device itself. However, other health workers preferred tablets over smartphones, mainly because the bigger size of the screen was perceived as easier for client engagement.	Schoen 2017; Shao 2015	Very low confidence	Due to serious concerns regarding relevance and adequacy, minor concerns regarding methodological limitations, and no/very minor concerns regarding coherence
34. Some health workers felt that sustainable, at scale mHealth programmes required approval and stewardship from political leaders, such as ministries of health. Leadership interest in mHealth interventions was described as motivating to health workers. Health workers suggested that such leaders should be engaged early and continuously throughout the programme, and be provided with evidence of effectiveness, so as to secure their support. The lack of high‐level stewardship impacted negatively on the mHealth programme.	Ginsburg 2016; Kolltveit 2017; Lodhia 2016	Low confidence	Due to serious concerns regarding adequacy, and no/very minor concerns regarding methodological limitations, coherence and relevance
35. Health worker accounts pointed to the strong influence of the health systems and social context in which the intervention was embedded. Contextual and systems issues, such as difference in language use between clients and health workers, gender discrimination, discomfort with professional hierarchies, poverty, resource constraints, staff attrition, and more, all of which were external to the technology and the physical device, influenced how health workers experienced mHealth and the use of mobile devices for service delivery, in their different contexts.	Chang 2011; Huq 2014; Khan 2015; Kolltveit 2017; Lodhia 2016; Praveen 2014; Rothstein 2016; Shao 2015; Tewari 2017; Toda 2017; van der Wal 2016; Wolff‐Piggott 2018	Moderate confidence	Due to no/very minor concerns regarding methodological limitations and relevance, but moderate concerns regarding coherence and adequacy
36. It was important for health workers that mobile health interventions be integrated with other existing electronic health information systems. This interoperability made it more likely that mobile devices would be integrated into standard care practices, while the absence of integration frustrated health workers.	Garg 2016; Ginsburg 2016; Lodhia 2016; Rothstein 2016	Moderate confidence	Due to no/very minor concerns regarding methodological limitations and coherence, but moderate concerns regarding relevance and adequacy
37. Health workers offered programmatic and implementation recommendations to improve mobile health interventions. The most cited of these was that the interventions be expanded to other settings and services, beyond what they were using it for as described in the studies. Other recommendations included raising community awareness about mHealth programmes, being involved in developing programmes, and appointing a 'mobile health champion'. Workers also suggested that those collecting surveillance data, must be informed of how the data are used.	Bacchus 2016; Barnabee 2014; Ginsburg 2016; Hao 2015; Khan 2015; Kolltveit 2017; Lodhia 2016; Madon 2014; Medhanyie 2015; Mitchell 2012; Murray 2015; Mwendwa 2016; Rothstein 2016; Schoen 2017; Toda 2017; van der Wal 2016	High confidence	Due to no/very minor concerns regarding, coherence, relevance, and adequacy, and moderate concerns regarding methodological limitations
38. Health workers had several technical recommendations to improve mobile health devices, for instance solar panels to counter poor electricity access and using photos to track clients' recovery from illness. Other recommendations included using sturdier devices, bigger screens, and having common applications, such as work scheduling on the devices.	Coetzee 2017; Henry 2016; Lodhia 2016; Praveen 2014; Quinn 2013; Schoen 2017	Moderate confidence	Due to no/very minor concerns regarding coherence, minor concerns regarding adequacy, and methodological limitations, and moderate concerns regarding relevance
39. The main challenges health workers experienced in using mobile devices, were poor network connectivity, access to electricity, and the costs to recharge devices. Solutions offered, included using solar panels, using the powered‐up phone of a colleague, or reverting back to the paper‐based system. Sometimes poor connectivity resulted in client dissatisfaction because it created delays in receiving health care. Health workers' commitment to their clients motivated them to cope with these and other challenges.	Chang 2011; Ginsburg 2016; Hampshire 2016; Ilozumba 2018; Khan 2015; Lodhia 2016; Madon 2014; Mwendwa 2016; Nguyen 2015; Praveen 2014; Quinn 2013; Schoen 2017; Toda 2017; van der Wal 2016; Watkins 2018	High confidence	Due to no/very minor concerns regarding coherence, relevance, and adequacy, and minor concerns regarding methodological limitations
40. Health workers expressed dissatisfaction with mobile devices when technology changes were too rapid, showed a dislike for typing, and were concerned that mHealth impersonalised their interaction with clients. Since these dissatisfactions were only infrequently raised within the data set, it is unclear if these perceptions reflect wider experience.	Bacchus 2016; Hao 2015; Schoen 2017; Valaitis 2005	Low confidence	Due to serious concerns regarding adequacy, moderate concerns regarding relevance, minor concerns regarding methodological concerns, and no/very minor concerns regarding coherence
41. Health workers discussed challenges, beyond network and electricity issues, that sometimes were just an annoyance or a concern, but at other times also impeded their mHealth activities, and their ability to provide a service assisted by the use of mobile devices. These included damaged devices, loss and theft of devices, having to carry two devices, not being able to readily buy phone credit when needed, not being able to send long messages because of character limitations, and the limitations of the language capabilities of their devices.	Chang 2011; Cherrington 2015; Coetzee 2017; Hampshire 2016; Hao 2015; Ilozumba 2018; Lodhia 2016; Medhanyie 2015; Murray 2015; Mwendwa 2016; Praveen 2014; Quinn 2013; Rothstein 2016; Toda 2017; Valaitis 2005; van der Wal 2016	Moderate confidence	Due to no/very minor concerns regarding coherence and relevance, minor concerns regarding methodological limitations, and moderate concerns regarding adequacy
42. Health workers complained when the tasks asked of them in mHealth interventions were felt to be beyond their clinical capacity, and when support from higher‐level workers was absent.	Orchard 2014; Praveen 2014	Very low confidence	Due to serious concerns regarding relevance and adequacy, moderate concerns regarding methodological limitations, and no/very minor concerns regarding coherence

## Background

Mobile health (mHealth) refers to medical and public healthcare practices supported by mobile devices, such as mobile and smartphones, client‐monitoring devices, personal digital assistants (PDAs), and tablets (WHO 2011). It also refers to these devices' capabilities to create, store, retrieve, and transmit information between users (Akter 2010). mHealth relies mainly on the mobile phone's utility of voice, short message services (SMS) and multimedia message services (MMS), but also includes more complex applications, such as global positioning systems, Bluetooth technology, and third and fourth generation mobile telecommunications (3G and 4G systems) (WHO 2011).

These devices leverage the reach and speed of mobile networks and mobile computing power to improve the reach of healthcare delivery (Leon 2012; West 2014), including the capturing, processing, and exchange of information (Gagnon 2009), holding the potential to transform aspects of health service delivery and health systems management (Qiang 2011; Tomlinson 2013). In pursuit of universal health coverage, mobile health has the potential to extend the scope, accessibility and quality of health services, to increase the accountability mechanisms, to expand the population base accessing health services, and to increase capacity of the healthcare workforce (Agarwal 2016; Labrique 2013a; Labrique 2013b; Mehl 2014).

The growing interest in mHealth as a research topic is reflected in the 25 effectiveness reviews published in the Cochrane Library (Appendix 1). Two overviews of reviews have also identified 29 systematic reviews (Marcolino 2018; Mbuagbaw 2015), of which 17 were non‐Cochrane Reviews. These Cochrane and non‐Cochrane reviews cover mobile health technologies that vary in their type and purpose, from the use of email for clinical communication between healthcare professionals (Pappas 2012), to the use of mobile phones for healthcare appointment reminders (Gurol‐Urganci 2013). The evidence on the effectiveness of mHealth cited in these reviews also varies. The overview of reviews from Marcolino 2018 shows mixed results and a lack of long‐term studies, although some evidence suggests an effect on some health outcomes. The growing importance and interest in mHealth is also reflected in the launch of two new journals, one of which is within the Lancet group of journals, namely *The Lancet Digital Health* (www.thelancet.com/journals), and *mHealth* (mhealth.amegroups.com).

### Description of the topic

This review synthesises evidence of how health workers perceive and experience their use of mHealth devices to provide and support primary healthcare services, defined in this review as either the first contact point of health care (Awofeso 2004), any rehabilitative, therapeutic, preventive and promotive health care (Global Health Watch 2011), being delivered at an individual or community level, or both (Muldoon 2006); or bringing healthcare services to where people work and live, which in particular applies to low‐income settings (Muldoon 2006).

These devices refer to mobile devices, such as mobile and smartphones, client‐monitoring devices, PDAs, and digital tablets, and particularly refers to these devices' capabilities to create, store, retrieve, and transmit information between users (Blaya 2010; Braun 2013; Catalani 2013; Hall 2014). Examples of how mHealth supports primary healthcare services, include: (i) clinical decision support during client consultation (Ginsburg 2016; Ilozumba 2018); (ii) information management to organise or deliver services, or both (Lodhia 2016; Ramirez 2017); (iii) health promotion messages to health workers and clients alike (Cherrington 2015); and (iv) communication between health workers, as well as between health workers and clients (Barnabee 2014; Chang 2011; Hampshire 2016). We use the terms 'mHealth' and 'mobile health' interchangeably in this review.

### Why is it important to do this review?

The release in April 2019, of the World Health Organization (WHO) guideline on digital interventions for health system strengthening (WHO 2019), attests to recognition at the highest level of global health, that mHealth is now a significant component in the delivery and support of healthcare policy, guideline and decision‐making processes. Processes, such as the development of this guideline, should be supported by “… social scientific studies explicating processes of technology adoption …” (Chib 2015). Identifying, appraising and synthesising the qualitative evidence of health workers' perceptions and experiences of mHealth programmes, complement the reviews of mHealth effectiveness and help improve our understanding of the barriers to, and facilitators of, its successful implementation (Chang 2013; Grimsbø 2012; Medhanyie 2015), as well as helping us to understand the outcomes, implementation, and feasibility of these programmes. This is particularly important as decision makers move from assessing the options to implementing the intervention, and thus need to consider more than whether an intervention works or not, but also the extent to which it may be acceptable in different contexts (Langlois 2018). This review is one of two qualitative evidence syntheses, that have been used alongside a suite of reviews of effectiveness, to inform the recently published WHO guidelines (WHO 2019); the other Cochrane Review focuses on clients' and peoples' perceptions and experiences of targeted digital communication, accessible via mobile devices for reproductive, maternal, newborn, child and adolescent health (Ames 2019).

### How this review might inform or supplement what is already known in this area

The Cochrane and non‐Cochrane effectiveness reviews (Agarwal 2018a; Agarwal 2018b; Agarwal 2018c; Braun 2013; Gonçalves‐Bradley 2018a; Gonçalves‐Bradley 2018b; Vasudevan 2018; Vervloet 2012), showed mixed or inconclusive results. In order to understand this heterogeneity, we need to go beyond the numbers and explore the context in which the interventions are delivered, and the experiences of the people involved in the delivery (Langlois 2018). This may lead to a better understanding of possible reasons why mHealth interventions have worked differently in different contexts. It is therefore, important to supplement the evidence of effectiveness by exploring the barriers and facilitators to the successful implementation of mHealth interventions, through qualitative studies that take contextualised experience into account (Glenton 2013). This would support the call by some of the effectiveness reviews that "… clients’ and healthcare providers' evaluation and perceptions of the safety of the interventions, potential harms, and adverse effects … should be assessed" (Gurol‐Urganci 2013), and "… barriers to trial development and implementation should also be tackled [in future studies]" (Atherton 2012). This qualitative evidence synthesis intends to be both complementary to the effectiveness reviews, as well as providing robust evidence in its own right.

## Objectives

To synthesise qualitative research evidence on health workers' perceptions and experiences of using mHealth technologies to deliver primary healthcare services, and to develop hypotheses about why some technologies are more effective than others.

## Methods

### Criteria for considering studies for this review

#### Types of studies

We included primary studies that used qualitative methods for data collection (e.g. interviews, focus group discussions, document analysis, and observations), and qualitative methods for data analysis (for instance, thematic analysis, and grounded theory). We excluded primary studies that collected data using qualitative methods but did not perform a qualitative analysis (e.g. open‐ended survey questions where the responses were analysed using descriptive statistics). We included mixed‐methods studies when it was possible to extract data that resulted from the qualitative methods. We included studies regardless of whether they had or had not been carried out alongside studies of effectiveness of mHealth.

#### Topic of interest

##### Study participants

We included studies that focus on the perceptions and experiences of the following.

1. All categories of health workers (i.e. professionals, paraprofessionals and lay health workers) who were involved in providing primary healthcare services to clients. We defined a paraprofessional health worker as someone with some form of secondary education and subsequent informal and/or formal training, lasting a few months to more than a year (Olaniran 2017). We defined a lay health worker as any health worker who performs functions related to healthcare delivery, is trained in some way to provide these functions, but has received no formal professional or paraprofessional certificate or tertiary education degree (Lewin 2005). Where appropriate, we distinguished between different categories of health workers, for example, health professionals and lay health workers.
2. Any other individuals or groups involved in delivering and managing mHealth programmes which aimed to provide or support primary healthcare services to clients. These individuals or groups included administrative staff, information technology staff, managerial and supervisory staff, they may have been based in a primary healthcare facility or in the community, but could also have been employed at a district or national level. The criterion was that they were to be involved in supporting the delivery of primary healthcare services or the mHealth programmes or both, irrespective of their placement.

Given the review's focus, i.e. health workers' use of mHealth to deliver primary healthcare services, we excluded the perceptions and experience of clients in this review. We also excluded participants identified as technical staff who developed and maintained the mHealth architecture used, for example, those involved in writing the software programmes or who provided technical support to the end users.

##### Settings

We included studies of mHealth programmes that were part of primary healthcare services delivery. For the purposes of this review, we defined 'primary healthcare services' as one or any combination of the following.

1. The first contact point of health care (Muldoon 2006).
2. All rehabilitative, therapeutic, preventive, and promotive health care (Global Health Watch 2011).
3. Being delivered at an individual or community level, or both (Muldoon 2006).
4. Bringing healthcare services to where people work and live, which in particular applies to low‐income settings (Muldoon 2006).

These services could be implemented in public or private primary healthcare facilities, in the community and workplace, or the homes of clients. We included studies conducted in any country.

While our review focuses on primary healthcare services as a micro‐level health system, we understand and acknowledge that these services are embedded within broader, meso‐level, i.e. district health systems, which deliver health care at secondary and tertiary levels (Gilson 2012; Langlois 2018). These district‐level systems are in turn, shaped by the socioeconomic, political, and health system contexts at a macro level, i.e. national and global levels (Langlois 2018). It is therefore, to be anticipated that the barriers and facilitators to the successful implementation of mHealth programmes will be found across the three tiers, ranging from micro‐level issues, such as protecting the confidentiality of primary healthcare client information (Labrique 2013), to meso‐ and macro‐level issues, such as reliable network coverage (Aranda‐Jan 2014), and the integration of mHealth platforms into higher‐level existing electronic health systems (Aranda‐Jan 2014), and that this will be reflected in the experience of participants.

##### mHealth interventions

This review focused on health workers' perceptions and experiences of their use of mHealth devices to provide and support primary healthcare services.

In this review, mHealth devices were defined as mobile devices that are used to create, store, retrieve, and transmit data in real time between users (see Appendix 2 for more technical definitions related to these devices).

We included interventions in which health workers used mobile devices to provide and support any type of primary healthcare service, which revolved around uni‐ and bi‐directional communication between health workers and clients, between health workers themselves, and between health workers and programme staff, other than health workers. In some instances, there was no direct interpersonal communication per se, but only health workers interacting with digital information available on the devices. Examples of these communications and interactions with data, included client registration and tracking, disease surveillance, various forms of decision support during consultations, for instance algorithms loaded on the devices, automated messaging to health workers, and stock notifications. We accessed the World Health Organization (WHO) taxonomy for digital health interventions (WHO 2018), and added their classification to our description of the technologies used in the included studies (Characteristics of included studies). According to this classification, digital health interventions are categorised according to targeted primary users, identified as: clients, health workers, health system or resource managers, and interventions for data services.

### Search methods for the identification of studies

#### Electronic searches

In our search for synthesised evidence, we searched PDQ‐Evidence (www.pdq‐evidence.org) and the Cochrane Library (www.cochranelibrary.com) for related reviews on 21 February 2018. We scanned any identified reviews to assess if any of the studies included or cited in the reviews could potentially also be included in our review.

We searched the following databases for primary studies without any language, date, or geographic restrictions.

1. MEDLINE Epub Ahead of Print, In‐Process & Other Non‐Indexed Citations, MEDLINE Daily and MEDLINE 1946 to present, Ovid (searched 12 January 2018)
2. Embase 1974 to 11 January 2018, Ovid (searched 12 January 2018)
3. CINAHL 1981 to present, EbscoHost (searched 11 January 2018)
4. Science Citation Index and Social Sciences Citation Index 1987 to present, and Emerging Sources Citation Index 2015 to present, ISI Web of Science (searched 12 January 2018) (topic search)
5. Global Health 1973 to 2015 Week 48, Ovid (searched 8 December 2015)

We did not search Global Health in 2018 as we had no access to this database.

#### Searching other resources

We screened the reference lists of all the included studies and key references (i.e. relevant systematic reviews).

##### Grey literature

We conducted a grey literature search in the following sources to identify studies not indexed in the databases listed above.

1. Eldis: www.eldis.org (searched 21 February 2018)
2. Google Scholar: scholar.google.co.za (searched 21 February 2018)
3. mHealth Database: www.africanstrategies4health.org/mhealth (searched 05 March 2018)
4. mHealth Evidence: www.mhealthevidence.org (searched 21 February 2018)
5. mHealth Knowledge: mhealthknowledge.org (searched 05 March 2018 )
6. mPowering: partnerships.usaid.gov/partnership/mpowering‐frontline‐health‐workers (searched 05 March 2018)
7. OpenGrey: www.opengrey.eu (searched 16 February 2018)
8. The Grey Literature Report: www.greylit.org (searched 21 February 2018)

The search strategies for the main databases can be found in Appendix 3.

We re‐ran the search strategies in February 2020. We screened these records and potentially relevant studies are awaiting classification; we will assess these studies at the next update.

#### Selection of studies

We collated all titles and abstracts identified through the search strategy into one reference management database Covidence. After removing duplicate records, each record was independently assessed by the first review author and any one of the other review authors, for its potential inclusion eligibility. We excluded records that were not relevant to the topic of this review. Thereafter, we retrieved the full text of all of the abstracts and titles that have been assessed as potentially eligible. Using the same process as for the abstracts, each full text was independently assessed by the first review author and any one of the other review authors, based on the review's inclusion criteria. To minimise bias, a review author was not permitted to assess a full text to which (s)he was an author. Given the high number of full texts we had to assess, we recruited an additional researcher and trained her to assist us with these assessments. We resolved disagreements between review authors through email correspondence and face‐to‐face discussions. When the two review authors could not reach consensus, we reverted to a team decision through email correspondence. In one instance, these email discussions resulted in a refinement of our inclusion criteria: though we included mobile health communication in our protocol, we did not specify the equipment used for emailing, and during a team discussion we agreed to exclude papers in which email was sent from stationery devices, such as a laptop used by a general practitioner in his/her consultation room. We contacted several study authors for more study information, when the information in the full text was insufficient to determine inclusion or exclusion of the study.

**Translation of languages other than English**

Abstracts of three studies required translation. Two of these were in Spanish and one in French. We translated the abstracts of these studies, using open source software (Google Translate: translate.google.com), and excluded the studies based on the translated version of the abstracts. No full‐text studies required translation.

#### Sampling from the included studies

We identified 23 studies from our 2015 search. We included all 23 studies in our analysis. In 2018, we repeated our search, and identified an additional 30 studies. While small sample sizes can lead us to have less confidence in a finding, large sample sizes can also threaten our ability to carry out a thorough qualitative analysis (Glenton 2018; Sandelowski 1995). We therefore decided to select a sample of these 30 studies. Several of the studies we had identified in our 2015 search had a number of methodological limitations. This had led us to downgrade our certainty in several of the findings we had developed during our first analysis. We therefore decided to sample studies from the 2018 search based on our assessment of their methodological limitations. While we had included all studies identified from our 2015 search regardless of their methodological limitations, we only included studies from the 2018 search that we assessed as having no to moderate concerns regarding their methodological limitations. This led us to sample 20 of the 30 studies from the 2018 search (see Table 2 for the exclusion reasons of the 10 studies we appraised as having serious methodological limitations). In the main, studies that were not sampled because of serious methodological limitations had poor descriptions of participant selection, data collection and analysis methods; thin data; and little information on author reflexivity.

**1:** Studies included but not sampled: methodological limitations

**Study ID**	**Title**	**Methodological limitation concerns**
Bardosh 2017	Operationalizing mHealth to improve patient care: a qualitative implementation science evaluation of the WelTel texting intervention in Canada and Kenya	Serious concerns due to insufficient information on study context, poorly described sampling and data analysis. The results are often written as generalisations, without being ascribed to particular participants, or particular participant groups. There was no reference to author reflexivity.
Braun 2016	An evaluation of a family planning mobile job aid for community health workers in Tanzania	Serious concerns due to insufficient information on the study context, sampling, data collection and analysis, and too few participant quotes to support their findings. There was no reference to author reflexivity.
Hamoy 2016	Real‐time Regular Routine Reporting for Health (R4Health): lessons from the implementation of a large scale mobile health system for routine health services in the Philippines	Serious concerns due to no explanation on their sampling, poorly described data collection and analysis, and no reference to author reflexivity.
Kabakyenga 2016	A demonstration of mobile phone deployment to support the treatment of acutely ill children under five in Bushenyi district, Uganda	Serious concerns due to no description of data analysis, author reflexivity, and insufficient information on the participants. It appears as if the study findings are more supported by the quantitative data than the qualitative data.
Knoble 2015	Electronic diagnostic algorithms to assist mid‐level healthcare workers in Nepal: a mixed‐method exploratory study	Serious concerns due to a very poor methods section, which made it impossible to appraise the study's methodology.
Missal 2016	Building capacity to use m‐Health in maternal, newborn and child health interventions	Serious concerns due to insufficient information on study context, sampling, data collection and analysis, and no reference to author reflexivity.
Modi 2015	Development and formative evaluation of an innovative mHealth intervention for improving coverage of community‐based maternal, newborn and child health services in rural areas of India	Serious concerns due to insufficient information on data analysis, author reflexivity, and poor data to support the study findings.
Jalloh‐Vos 2013	Mobile health: connecting managers, service providers and clients in Bombali district, Sierra Leone	Serious concerns due to insufficient information on sampling, data collection and analysis, and no reference to author reflexivity. It is also a serious concern not knowing if the cited data refer to mid‐ or end‐intervention time points.
Shieshia 2014	Strengthening community health supply chain performance through an integrated approach: using mHealth technology and multilevel teams in Malawi	Serious concerns due to insufficient information on participant demographics, sampling, data collection and analysis, and no reference to author reflexivity.
van Heerden 2017	App‐supported promotion of child growth and development by community health workers in Kenya: feasibility and acceptability study	Serious concerns due to insufficient information on data collection, and no reference to author reflexivity. There is insufficient data to support the study findings.

#### Data extraction, analysis and synthesis

It should be noted that our data coding, extraction, synthesis, and writing of findings, were conducted in two stages. We had already completed these steps for the 23 included studies from the first search (done in 2015), by the time we began the same process for the 20 included studies from the second search (done in 2018). The details of both stages are presented below.

For both the 2015 and 2018 search studies, we extracted study information, such as country, the health worker category, the healthcare issue addressed, and the specific mobile health technology used, into an Excel spreadsheet. This served as a tool to refer to the study details during the data extraction and coding.

The data coding, extraction and synthesis process was an iterative process, aligned with the thematic synthesis process outlined by Thomas 2008. For the 23 included studies from the first search, two review authors (WO, KD) independently read each study as a whole, including the background, methods, results, discussion, and conclusions sections, to get a sense of their meaning and their contribution to answering the review question. Each review author thereafter conducted a line‐by‐line coding of the data of the first study. They then met and agreed on the codes and supporting data. They used this code list to code the second paper, thus beginning the process of translating the data from one study into the next. New codes that emerged from the second, and subsequent studies, were added to the list, and we returned to the already coded studies, to determine if these codes applied to that data also. As the code list was amended, the authors began the process of organising the codes into broad themes, which in some cases had subthemes attached to it. Using the thematically coded data, the same two review authors jointly wrote up discreet findings. Since many of the extracts did not neatly fit within any theme, we continued the iterative process of trying to make sense of the extracts, by regrouping them with other extracts from which similar underlying issues had emerged, and eventually synthesised all the extracted data into findings.

The same two review authors (WO and KD) that led the analysis for the first 23 studies, did so for the 20 new studies from the 2018 search. By the time we started coding these 20 new studies, we already had an existing list of themes and subthemes to use as a deductive coding framework. However, we were cognisant that the new set of studies might yield data not yet captured in our framework. We therefore approached the analysis both deductively and inductively, reading the data to determine if and where it fit within the existing framework, and for what new insights it yielded. Data extracts were therefore grouped by WO and KD, both into existing categories, as well as into new categories that emerged from the data. Upon completing this for all the new studies, one review author (WO) amended the texts of the existing findings to reflect the additional data. The rest of the author team verified that all the supporting data were reflected in the amended and new findings. Upon completing this for all the new studies, one review author (WO) amended the texts of the existing findings to reflect the additional data. We also constantly evaluated each extract against our inclusion criteria and review objectives, deciding up until the very end, whether or not it was an appropriate fit. The findings thus represent the final translation of the coded data across all of the 43 included sample studies.

The aim of the data synthesis was to develop a set of findings we believe represent a trustworthy, coherent, and detailed understanding of the perceptions and experiences of those who deliver and support primary healthcare services through using mobile devices. As detailed above, we synthesised the coded data into a set of 42 discreet findings. Thereafter, the one review author (WO) involved in drafting the findings, thematically analysed these findings and grouped them into four overarching themes. These themes provide a coherent overview of our findings.

#### Assessing the methodological limitations of included studies

At a minimum, all included studies had to have used qualitative data collection and analysis methods. Prior to the data coding, extraction, synthesising, and writing the findings from both searches, two review authors (WO, KD) independently assessed the methodological limitations of the included studies using an adapted Critical Appraisals Skills Programme (CASP) tool (Atkins 2008). We assessed each study on the following nine criteria.

1. Adequately described setting and context
2. A well described sampling strategy that is appropriate
3. A well described data collection strategy that is appropriate
4. An adequately described data analysis method that is appropriate
5. Sufficient evidence to support the claims made/findings
6. Adequate evidence of researcher reflexivity
7. Demonstrated sensitivity to ethical concerns
8. Adequately described study limitations
9. Any other concerns raised by the review authors

Based on their assessment, the two review authors (WO, KD) independently graded each study as having no, or very minor, minor, moderate, or serious methodological limitations. Thereafter, they met and reached consensus on their respective assessments.

#### Assessing our confidence in the synthesis findings

Three review authors (WO, JAW, KD) used the GRADE‐CERQual (Confidence in the Evidence from Reviews of Qualitative research) approach to summarise our confidence in each finding (Lewin 2018).

GRADE‐CERQual assesses confidence in the evidence, based on the following four key components.

1. Methodological limitations of included studies: the extent to which there are concerns about the design or conduct of the primary studies that contributed evidence to an individual review finding.
2. Coherence of the review finding: an assessment of how clear and cogent the fit is between the data from the primary studies and a review finding that synthesises those data. By cogent, we mean well supported or compelling.
3. Adequacy of the data contributing to a review finding: an overall determination of the degree of richness and quantity of data supporting a review finding.
4. Relevance of the included studies to the review question: the extent to which the body of evidence from the primary studies supporting a review finding is applicable to the context (perspective or population, phenomenon of interest, setting) specified in the review question.

After assessing each of the four components, we made a judgement about the overall confidence in the evidence supporting the review finding. We judged confidence as high, moderate, low, or very low. A sample (40%) of the final assessment was peer reviewed by a fourth review author (NL), and we adjusted some of the assessments after reaching consensus with the fourth review author. We started with high confidence in all findings, and then downgraded any findings where we had important concerns regarding any of the GRADE‐CERQual components.

#### Summary of qualitative findings table and evidence profiles

We presented summaries of the findings and our assessments of our confidence in these findings in Table 1. We presented detailed descriptions of our confidence assessment in Appendix 4.

#### Linking the review findings to Cochrane intervention Reviews

We sought to understand how our findings were related to, and could help to inform, the findings of six of the Cochrane Reviews of effectiveness that were used to inform the WHO guideline on digital interventions for health system strengthening (WHO 2019). These reviews assessed the effectiveness of the following mHealth interventions.

1. Birth and death notification via mobile devices (Vasudevan 2018)
2. Stock notification and commodity management via mobile devices (Agarwal 2018a)
3. Client to provider telemedicine (Gonçalves‐Bradley 2018a)
4. Tracking of client's health status and services received (Agarwal 2018b)
5. Health provider decision support via mobile devices (Agarwal 2018c)
6. Health provider to health provider telemedicine (Gonçalves‐Bradley 2018b)

Each of these interventions was also the topic of a recommendation in the WHO guideline (WHO 2019).

As part of the WHO's guideline process, our qualitative evidence was used as a source of information about intervention acceptability and feasibility. The WHO technical team prepared GRADE evidence‐to‐decision tables for each recommendation. Each table included evidence from the relevant Cochrane Review of effectiveness. In addition, each table included evidence from this qualitative evidence synthesis regarding the acceptability and feasibility of each intervention. The WHO's technical team prepared these tables, with input from the review authors. The technical team and review authors of this synthesis also collaborated on a supplementary document presenting evidence about the acceptability and feasibility of all these interventions. The guideline panel used these tables and supplementary documents as the basis for their recommendations.

While our review was not directly linked to the effectiveness reviews, the findings from our review may be used to shed light on the outcomes observed in the effectiveness reviews, by offering insight into contextual factors, including health worker preferences, that may have influenced outcomes, either positively or negatively. Furthermore, the findings from our review may be used to develop hypothesis for subsequent consideration and assessment in future effectiveness reviews, seeking to understand why some mHealth technologies are more effective than others.

#### Review author reflexivity

The review author team represents diverse professional backgrounds, with a range of research experiences and expertise that could have influenced their input in conducting this review. All of them are experienced qualitative researchers. Except for one review author (KD), everybody has had previous experience in conducting primary mHealth research in the context of primary healthcare services in low‐income settings in South Africa, and have published on this (Coetzee 2017; Leon 2012; Neupane 2014; Watkins 2018). FG has also experience in conducting telemedicine research in high‐income contexts (Griffiths 2017). Our experiences in conducting effectiveness studies and process evaluations of mobile health programmes, included positive, negative, and mixed results. This provided us with a good platform for engaging and understanding the complexities and nuances of qualitative research of mobile health interventions.

The review authors reflected on the influence our perspectives might have on the conduct of the review, and in some cases tried to moderate this influence, in a number of ways. During the screening of abstracts/titles and full texts, the team constantly referred to each other to resolve conflicts, and in many instances a team decision was called upon. As is standard practice within qualitative research, the two review authors (KD, WO) who did the data coding, extraction, and synthesising, and wrote the findings, constantly discussed with each other how their own background and position, may have affected their analysis and writing of the findings.

WO realised that at times his research experiences resonated strongly with some of the included studies, and was aware that this could lead him to give these data more importance than was due. Conversely, he was aware that he could be more dismissive towards data which contradicted his experiences. KD questioned the weight he attributed to certain data, ensuring that all data were equally represented in the final set of findings. WO and KD repeatedly questioned each other's interpretation of the data and how it fitted with the existing findings. They also called upon other members of the author team to verify that the findings were reasonable reflections of the supporting data. JAW, KD, and WO also used the same process of constant discussion and being aware of their personal perspectives when appraising their confidence in the findings. Finally, the contact editor of this review read each finding and its supporting data closely. She pointed to any mismatch between the supporting data and a finding, and critically engaged with our interpretation of the data, which led to a refinement of our analysis and writing of the findings.

## Results

### Results of the search

We screened 7225 records. Fifty‐three studies met our inclusion criteria. We purposively sampled 43 of these studies for inclusion in our analysis (Figure 1). All of the sampled studies were published between 2005 and 2018; see Methods section ‐ 'Sampling from the included studies' for a description of how we sampled these studies. In February 2020, we re‐ran the search strategies. We screened those records and 85 studies that we identified as potentially relevant are listed under Studies awaiting classification; we will assess these studies at the next update.

**1:**
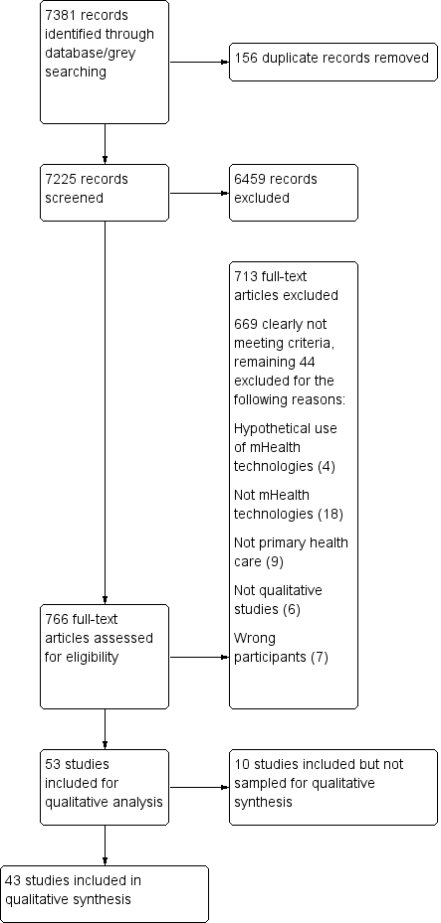
PRISMA flow diagram

### Description of the included studies

In this section, we describe the studies that we sampled for analysis. For a more detailed description of each included and sampled study, see Characteristics of included studies. For an overview of the studies that we included but did not sample, see Table 2*.*

#### Study participants

In 17 (40%) of the sampled studies, the participants included both lay health workers and a range of professional health workers, such as nurses, paramedics, doctors, midwives, pharmacists, and laboratory staff. Non‐medical professional participants, found in four studies, referred to programme managers, administrators, decision makers, and social workers. A further 10 (23%) and 12 (27%) studies only included lay health workers or health professionals, respectively. In four (9%) of the studies, the participants were only described as ‘healthcare providers’.

#### Settings

Thirty‐two (74%) of the sampled studies were from low‐ or middle‐income countries: Bangladesh (3), Brazil (1), India (4), Ethiopia (1), Ghana + Malawi (1), Ghana (2), Kenya (6), Lesotho (1), Rwanda (1), South Africa (4), Swaziland (1), Tanzania (3), Timor‐Leste and Myanmar (1 from each country), and Uganda (2). The remaining 11 studies (26%) were from high‐income countries: Australia (1), Canada (2), Ireland (1), Norway (1), Scotland (1), and USA (5). In 12 (28%) of the studies, the mobile devices were used to provide health care in clients' homes, and in nine (21%) of the studies, they were used in clinics. A further nine (21%) studies implemented in clinics plus another setting. Only two (4%) of the studies took place in a hospital, one (2%) in a general practice, and one (2%) in a school. The type of healthcare setting was unclear in the remaining nine (21%) studies. Most of the studies (14, 32%) were implemented in a rural setting, 11 (26%) in urban settings, and four (9%) in a rural and urban setting. Another four (9%) of the studies were conducted in rural and per‐urban communities, and in 10 (23%) of the studies, the setting was not detailed enough to classify.

#### Types of mobile devices

Mobile phones, also described in the studies as 'iPhones', or 'smartphones', were used in 31 (72%) of the sampled included studies, with either personal digital assistants (PDAs) and tablets in 11 (26%) studies. A combination of mobile phones and tablets was used in one study. Most of the mobile health interventions, in total 18 (42%) of the studies, comprised of software loaded onto the mobile device which guided the consultation process, for example, a screening algorithm that allowed health workers to diagnose clients. In the remainder of studies, the intervention comprised of in‐person and/or text message communication, collecting of surveillance data, and a range of other interventions, such as health promotion materials in the form of videos on the devices, accessing the Internet, and in one instance, allowing low‐level workers to send wound care images to higher‐level workers for treatment advice. The healthcare issue addressed through the mobile health programmes was in the main, maternal, neonatal and child health, with 17 studies (40%) reporting on this. Other healthcare issues included communicable and non‐communicable diseases, cardiovascular diseases, and intimate partner violence.

### Methodological limitations of the studies

Details of our assessments of the methodological limitations of the individual sampled studies can be found in Table 3. We assessed 10 studies as having no or very minor methodological limitations, 13 as having minor methodological limitations, and 14 and six studies respectively, as having moderate and serious methodological limitations. Of the 43 studies, 41 were published in peer‐reviewed journals, and two as full Masters theses (Barnabee 2014; van der Wal 2016). Most peer‐reviewed journals set a word limitation, which is not suitable for the comprehensive reporting of qualitative research. Our concerns regarding the general lack of rich data and thick description, as well as often poor descriptions of how participants were sampled and recruited, and of researcher reflexivity, may be attributable to these word limitations. Many studies provided poor descriptions of ethical considerations, apart from mentioning that ethical approval was obtained. All studies provided at a minimum a brief description of the study context, who the participants were, the mHealth programme, and to some extent, the study limitations.

**2:** Methodological limitations of the included studies

**Study**	**Was the context described?**	**Was the sampling appropriate and described?**	**Was the data collection strategy appropriate and described? **	**Was the data analysis appropriate and described? **	**Were the findings reported supported by evidence? **	**Is there evidence of researcher reflexivity?**	**Have ethical issues been taken into consideration?**	**Are study limitations discussed? **	**Any other concerns?**	**Overall assessment of methodological limitations^e^**
Ayiasi 2015	Yes^a^	Mostly^b^	Yes	Mostly	Mostly	No	No	Insufficient^c^	No^d^	Moderate
Bacchus 2016	Mostly	Yes	Yes	Yes	Yes	Yes	Yes	Yes	No	No/very minor
Barnabee 2014	Yes	No	Yes	Yes	Mostly	Yes	Yes	Yes	No	Minor
Chang 2011	No	No	Insufficient	Insufficient	Yes	No	Insufficient	Insufficient	No	Moderate
Cherrington 2015	Yes	Yes	Mostly	Yes	Yes	No	Insufficient	Mostly	Yes	Minor
Coetzee 2017	Mostly	Mostly	Yes	Yes	Yes	Mostly	Yes	Yes	No	No/very minor
Garg 2016	Mostly	Mostly	Mostly	Yes	Yes	Insufficient	Mostly	Yes	Yes	No/very minor
Ginsburg 2016	Yes	Yes	Yes	Yes	Yes	Insufficient	Mostly	No	No	Minor
Hampshire 2016	Yes	Yes	Yes	Mostly	Yes	No	Yes	No	No	Moderate
Hao 2015	Yes	Yes	Yes	Yes	Yes	No	Yes	No	No	Moderate
Henry 2016	No	No	Insufficient	No	No	No	Insufficient	Insufficient	Yes	Serious
Hirsch‐Moverman 2017	No	No	Insufficient	Insufficient	Insufficient	No	No	No	No	Serious
Huq 2014	Yes	Yes	Yes	Yes	Yes	Yes	Yes	No	No	No/very minor
Ilozumba 2018	Yes	Mostly	Mostly	Yes	Yes	Yes	Yes	Mostly	No	Minor
Jennings 2013	Yes	Mostly	Yes	Yes	Yes	No	Insufficient	Yes	No	No/very minor
Jones 2012	Yes	Yes	Mostly	Mostly	Yes	No	Insufficient	No	No	Minor
Khan 2015	Yes	Yes	Mostly	Insufficient	Yes	No	Insufficient	No	No	Moderate
Kolltveit 2017	Yes	Yes	Yes	Yes	Yes	No	Yes	Yes	No	No/very minor
Lodhia 2016	Mostly	Yes	Yes	Yes	Yes	No	Mostly	Yes	No	Minor
Madon 2014	Mostly	Yes	Yes	Yes	Yes	Insufficient	Insufficient	Mostly	No	Moderate
Medhanyie 2015	Insufficient	Insufficient	No	Insufficient	No	No	Insufficient	Yes	No	Serious
Messinger 2017	Yes	Yes	Yes	No	Yes	No	Yes	No	No	Moderate
Mitchell 2012	No	Mostly	Insufficient	Mostly	Mostly	No	No	Yes	No	Serious
Murray 2011	Insufficient	Mostly	Insufficient	Yes	Yes	Insufficient	Yes	Yes	No	Minor
Murray 2015	Mostly	Yes	Yes	Insufficient	Yes	No	Insufficient	No	No	Moderate
Mwendwa 2016	Yes	Yes	Yes	Mostly	Yes	Insufficient	Yes	No	No	Minor
Nguyen 2015	Mostly	Mostly	Yes	Yes	Yes	No	No	Mostly	Yes	Moderate
Orchard 2014	No	No	No	No	No	No	Insufficient	No	No	Serious
Praveen 2014	Yes	Yes	No	Yes	Yes	No	Yes	No	No	Minor
Quinn 2013	Mostly	Yes	Mostly	Mostly	Yes	No	Yes	No	No	Moderate
Ramirez 2017	Insufficient	Yes	Yes	Yes	No	Mostly	No	Yes	No	Moderate
Rothstein 2016	Yes	Yes	Yes	Yes	Yes	Mostly	Mostly	Yes	No	No/very minor
Schoen 2017	Mostly	No	Yes	Yes	Yes	No	Insufficient	Mostly	Yes	Minor
Shao 2015	Yes	Yes	Yes	Yes	Yes	Yes	No	Yes	No	Minor
Surka 2014	Mostly	Yes	Yes	Insufficient	Mostly	No	No	No	No	Serious
Tewari 2017	Yes	Yes	Yes	Yes	Yes	No	Mostly	No	No	Minor
Toda 2017	Yes	Mostly	Mostly	Yes	Yes	No	Mostly	Yes	No	Moderate
Valaitis 2005	Yes	Yes	Mostly	Yes	Yes	Mostly	Insufficient	Mostly	No	No/very minor
van der Wal 2016	Yes	Yes	Yes	Yes	Yes	Yes	Yes	Yes	No	No/very minor
Vedanthan 2015	Yes	Yes	Yes	Mostly	Yes	No	Mostly	Yes	No	Moderate
Watkins 2018	Mostly	Yes	Yes	Yes	Yes	Yes	Yes	Yes	Yes	No/very minor
Westergaard 2017	Yes	Yes	Yes	Mostly	Yes	No	Mostly	Yes	Yes	Moderate
Wolff‐Piggott 2018	Yes	Mostly	Yes	Mostly	Yes	Mostly	Yes	Yes	No	Minor

^a^Yes: the component was sufficiently, clearly, and appropriately described in the study. ^b^Mostly: the component was mostly described in the study, but would have been further strengthened with more detail. ^c^Insufficient: the study only offered a limited description of the component. ^d^No: the component was not described in the study. ^e^No or very minor concerns/minor concerns/moderate concerns/serious concerns.

### Confidence in the review findings

Out of 42 review findings, we had high confidence in 13 findings, moderate confidence in 18, low confidence in six, very low confidence in five (Table 1). Our explanation for each GRADE‐CERQual assessment is shown in the evidence profile in Appendix 4.

### Review findings

From the synthesised data, we drafted 42 individual findings (Table 1), which we organised into four overarching themes. Theme 1 deals with how mHealth changed how health workers worked with each other, in particular through connecting lower‐level health workers with higher‐level health workers, and peers with each other. Theme 2 describes how mHealth changed how health workers delivered care, and includes health workers' perceptions and experiences about issues, such as accessing information from the Internet, providing care over distance, and using treatment algorithms. In Theme 3, we present how mHealth led to new forms of engagement and relationships with clients and communities, mainly because mHealth allows direct, and often bi‐directional communication between health worker and client. This theme also covers issues such as elevated health worker status that comes from health workers using modern technology and needing to protect client information on their devices. Theme 4 details how health workers' use and perceptions of mHealth can be influenced by factors tied to costs, the health worker, the technology, the health system and society, and how poor network access and poor access to electricity could make mHealth difficult.

#### Theme 1: mHealth changed how health workers worked with each other

**Finding 1: Through being connected to other health workers and across various healthcare services, health workers appreciated that mobile devices allowed them to better co‐ordinate the delivery of care** (moderate confidence in the evidence)

Mobile devices enabled real‐time communication between health workers (Henry 2016; Huq 2014; Lodhia 2016; Madon 2014; Messinger 2017; Murray 2015; Quinn 2013; Ramirez 2017; Rothstein 2016; Schoen 2017; van der Wal 2016; Watkins 2018), and across various healthcare services (Khan 2015; Quinn 2013; Toda 2017; Watkins 2018), for example between home‐based care and emergency services (Barnabee 2014; Chang 2011; Hampshire 2016; Huq 2014; Mwendwa 2016), using the short message service (SMS) on a mobile phone to send prescriptions on behalf of clients (Khan 2015), and ordering supplies using a mobile device (Chang 2011; Hampshire 2016): "When there is shortage of drugs, we put it on WhatsApp so that colleagues will inform us if they have excess for us to borrow. Some indicate which drugs they have run out of so that we will avoid referring patients there" (Hampshire 2016). Health workers found mobile devices particularly useful in emergency situations, such as calling an ambulance to transport a distressed client to a health facility (Barnabee 2014; Hampshire 2016; Mwendwa 2016), and reporting disease outbreaks to healthcare facilities (Henry 2016; Toda 2017): "*…* while I was attending a patient in [village A], I received a notification from a patient in [village B]. I cannot leave the patient...I used Liga Inan [mHealth application on the mobile phone] to contact the health center in [town C] and they came with an ambulance and took the patient (P2)." (Barnabee 2014). Improved co‐ordination also resulted from mobile devices, which allowed easier screening, diagnosing, and prioritising of clients (Lodhia 2016; Rothstein 2016; Schoen 2017; van der Wal 2016; Watkins 2018), provided that its accuracy was established (Lodhia 2016). Mobile devices also allowed collaborative care when members of a care team shared clinical data and treatment plans of clients, (Ramirez 2017; Rothstein 2016; Watkins 2018), which resulted in tailoring services to clients' needs (Murray 2015).

**Finding 2: Lower‐level health workers valued being able to reach higher‐level health workers via mobile devices, and perceived the advice and support they received as improving their care and as satisfying to clients. When higher‐level professionals responded in anger, it made lower‐level health workers reluctant to call them** (moderate confidence in the evidence)

Apart from facilitating contact between same‐level health workers (Finding 1), lower‐level health workers particularly valued being able to reach higher‐level health workers through mobile devices (Ayiasi 2015; Chang 2011; Cherrington 2015; Hampshire 2016; Huq 2014; Khan 2015; Lodhia 2016; Messinger 2017; Mwendwa 2016; Quinn 2013; Toda 2017; van der Wal 2016; Watkins 2018). They received advice and support from the higher‐level health workers, such as when the latter was asked to intervene with clients refusing treatment (Ayiasi 2015), or had to advise treatment (Cherrington 2015; Huq 2014; van der Wal 2016): "… senior staff nurse, [higher‐level worker] commonly wants to know the detail of patient's condition and then advises [us lower‐level workers] over phone" (Huq 2014). Lower‐level health workers appreciated the immediacy of this support and advice (Chang 2011; Cherrington 2015; Madon 2014; Watkins 2018). These workers perceived this as improving the quality of their care and health outcomes (Ayiasi 2015; Chang 2011), as well as being satisfying to clients (Ayiasi 2015; Cherrington 2015): "They [clients] would feel like, "Oh they care", because if it was something that I didn't know the answer to and then, (someone) would get back with them" (Cherrington 2015). The exchange broke the hierarchy between health worker categories, when previously unreachable professionals could more easily be reached (Chang 2011; Huq 2014; Khan 2015). In one study, health workers perceived direct contact as improving relationships between lower‐ and higher‐level workers (Chang 2011). In contrast, when higher‐level professionals responded in anger, it made lower‐level health workers reluctant to call them (Ayiasi 2015).

**Finding 3: When higher‐level health workers failed to respond and support lower‐level workers through mobile devices, lower‐level staff had negative perceptions of these devices. One study emphasised the importance of having health professionals' buy‐in with mobile health to ensure that mobile devices were optimally used to support lay health workers** (moderate confidence in the evidence)

The data suggests that those participants who expressed an opinion desired good communication and co‐ordination with their seniors and others in their health systems context. Lack of anticipated co‐ordination, support and responsiveness, in particular in emergency cases (Mwendwa 2016), through mobile technology's connectedness with higher‐level staff or emergency services, led to a negative experience of the intervention amongst lower‐level health workers (Cherrington 2015; Huq 2014; Mwendwa 2016;Quinn 2013; Toda 2017): [Lower‐level health worker]: "The technology is good but let the higher levels take it seriously otherwise there is no need of sending the instant reports. The last time is [sic] sent a suspected tetanus baby but he died without anyone coming to see the child at the facility I had referred to" (Toda 2017). This lack of responsiveness was described as limiting the effectiveness of the intervention (Mwendwa 2016). In contrast, direct communication was expressed as having a positive impact on the facility and in turn on client experience. In one instance, a lack of managerial interest in mHealth‐facilitated disease surveillance demotivated the lower‐level workers from consistently using mHealth for their work (Toda 2017). In one study (van der Wal 2016), matters were complicated when the supervising doctor was not given a smartphone, as he could not supervise what the lower‐level health workers were doing with the phones. His lack of a phone led to his lack of supporting these workers and encouraging them not to use the intervention application either. In turn, these lower‐level workers wanted him to be given a phone, not only for his buy‐in, but also because they believed that this would enhance how they worked together.

**Finding 4: The use of mobile devices allowed some health workers to feel connected to their peers within their own organisations. However, others preferred face‐to‐face communication with their peers** (moderate confidence in the evidence)

Some health workers reported positively on being connected with their peers within their respective organisations through mobile devices (Barnabee 2014; Hampshire 2016; Watkins 2018), which some perceived as supportive (Henry 2016; Jennings 2013; Madon 2014). This was particularly the case when they were seeking advice to deal with clients experiencing complications (Barnabee 2014; Henry 2016): "In case you are stuck, you don't know what to do here, you don't know what to do anyway, you just communicate immediately [with other public health officers] and you will get the information immediately. So it has really made our work to be easy" (Henry 2016). However, others preferred face‐to‐face connection with their peers (Valaitis 2005; van der Wal 2016): "If people weren't coming into the office as regularly, then we would have to meet as a team more often just to do that informal [peer connection]" ..."I've always come in once a week, because I'm dying to see everybody" (Valaitis 2005).

**Finding 5: Some health workers relayed that mobile devices improved their reporting to supervisors and encouraged them to report more truthfully. Others compared mobile device‐facilitated supervision to "big brother watching". Some supervisors thought that mobile devices allowed them to better identify staff who needed support** (moderate confidence in the evidence)

Some health workers felt that mobile devices improved reporting to their supervisors (Barnabee 2014; Medhanyie 2015), their relationship with their supervisors (van der Wal 2016), and encouraged truthful reporting (Chang 2011). On the other hand, there were supervisors who did not think that mobile devices were a safeguard against false reporting (Jennings 2013). Mobile health‐facilitated supervision left some supervised workers with a sense of "big brother [is] watching" (Valaitis 2005). Mobile health resulted in work being more visible to the supervisors (Henry 2016). In instances where clients complained about health workers who did not visit them, workers reverted to having clients signing a paper record for such proof, and suggested electronic signatures be made available on their devices (Schoen 2017). In the context of conducting disease surveillance, health workers expressed a need for face‐to‐face interactions with those overseeing the surveillance (Toda 2017). Some supervisors expressed that mobile technology‐facilitated supervision allowed them to be more aware of their staff's work, in particular when the latter experienced problems. These supervisors perceived this increased awareness of staff's performance as positive because they could address the problems that came to their attention as a consequence (Henry 2016; Madon 2014; Mwendwa 2016): "[WhatsApp] has made me learn a thing or two, it has made me get to know characters as far as community health volunteers are concerned … I can even gauge performance when it comes to community health volunteers" (Henry 2016).

**Finding 6: Health workers had positive experiences with using instant messaging through WhatsApp. This application was seen as cheap and suitable for a range of activities, such as communicating with peers and posting photos as evidence of work done** (very low confidence in the evidence)

Some health workers valued instant messaging through WhatsApp (Hampshire 2016; Henry 2016; Schoen 2017), because it was perceived to be a cheaper way to communicate, compared to using short message services (SMS) (Henry 2016). They used WhatsApp for a range of activities, including communicating with peers, notifications when drugs were out of stock, supervision, and posting photos as evidence of work done (Hampshire 2016, Henry 2016): "It [WhatsApp] has been the best evidence. If I assess a child, I take a photo, or if I have attended any sick person in the community and I post on WhatsApp. If we [in Kibera] don't post, they will say in Makueni we don’t work" (Henry 2016). Some created groups on WhatsApp to serve different projects and interests (Henry 2016).

**Finding 7: Even when health workers received messages that were automated, rather than sent directly from a manager or supervisor, this was still experienced and responded to, as a kind of supervision. Some lower‐level health workers experienced it as supportive to their work, while others felt guilty for not providing correct care as per these messages** (low confidence in the evidence)

Automated text messages about illness and client management, and in one study, motivational messages (Jones 2012), sent to lower‐level health workers' mobile phones, were perceived by some of them as supervision (Jones 2012), and they felt it improved their care and knowledge (Cherrington 2015; Ilozumba 2018; Jones 2012; Mwendwa 2016). Workers valued the messages' conciseness, and saw it as providing up‐to‐date information and as being a useful reminder to provide correct treatment (Jones 2012): "It kept me on task...if I forgot, I would turn it on and it would pop up, "You're late"...it was wonderful" (Cherrington 2015). Some felt motivated by receiving automated treatment messages, but others felt guilty for not providing correct care as recommended by these messages (Jones 2012). There were concerns that the text messages were too repetitive in the information they carried, and that motivational messages on their own, without treatment guidance, were less meaningful than those with treatment guidance (Jones 2012).

#### Theme 2: mHealth changed how health workers delivered care

**Finding 8: The task optimisation enabled through mHealth interventions was widely valued by health workers** (moderate confidence in the evidence)

Through the use of mobile devices, health workers were able to expand their current range of tasks, at their own level (Chang 2011; Ilozumba 2018; Khan 2015), as well as take on tasks previously assigned to higher‐level workers (Barnabee 2014; Kolltveit 2017; Lodhia 2016; Praveen 2014): "In this setting ASHAs [community health workers] became proficient in not only performing risk factor measurements, but also in interpretation of the results: Earlier, I just used to go and measure BP [blood pressure], but with this tablet, I came to know what was a normal reading and how the actual reading differs from normal readings" (Praveen 2014), and conducted these tasks independently (Chang 2011). They experienced this as beneficial in improving their skills (Barnabee 2014; Ilozumba 2018; Khan 2015; Praveen 2014), and perceived it as addressing staff shortages (Lodhia 2016). Some health professionals reported that clients with mobile devices first contacted lay health workers before contacting them, which allowed the professionals to focus on clients more in need of their support: "The use of mobile phones typically led to more Patient–PHW [peer health worker, working at lower level than professional staff] communication and less Patient‐Staff." One staff member noted: "Instead of calling us, they first call the PHW … If the PHW can solve it, they don’t bother to call us [higher‐level facility staff]. If they cannot solve it, then the PHW calls us" (Chang 2011). The new tasks that mHealth allowed health workers to perform, were reported to facilitate improved interaction with clients (Barnabee 2014).

**Finding 9: At times, health workers used their mobile devices to access the Internet for health information, and found it useful when they were with clients who needed the information. This interaction also included health workers providing clients with additional information beyond the healthcare intervention. But, if the only way that health workers could access online information, required them to use their own money to purchase data, then this could be prohibitive to them accessing such information** (low confidence in the evidence)

Some health workers used their mobiles devices to gain access to the Internet where they accessed health information, clinical guidelines, health promotion material, and other information thought to be needed by clients (Bacchus 2016; Hampshire 2016; Schoen 2017; Watkins 2018): "Doctors and nurses who used the Internet for work reported using search engines on their phones such as Google, to access clinical information on diseases or prescription drugs. A few nurses reported accessing the digital versions of government clinical guidelines" (Watkins 2018). They found the quick access to such information useful, particularly when they were with clients who needed more information about a certain condition and its treatment (Hampshire 2016). The cost of data, when borne personally, sometimes prevented health workers from searching for information (Watkins 2018). In one study, health workers used their tablets to give non‐health information to clients who wanted to further their education, and thus needed access to information to enable reaching this goal: "We don’t have tablets usually so I used the tablet to do some personality tests of my clients who wanted to be in school” (Bacchus 2016).

**Finding 10: mHealth held the promise of increasing service efficiency for many health workers, but the experience of whether this promise was borne out in practice, varied in the accounts of health workers. It was experienced as efficient if it improved feedback, speed and workflow, but inefficient when the technology was slow and time consuming. Some were concerned that if mHealth was too efficient, making work faster, that this may justify staff cutbacks** (high confidence in the evidence)

Health workers' experiences of the efficiency of mobile devices varied across and within studies. In general, efficiency was related to work being done more quickly, whilst inefficiency was related to the extra time and work it took when using these devices. Health worker accounts of speed efficiency related to enhanced communication and information flow (Ayiasi 2015; Cherrington 2015; Hao 2015; Ramirez 2017; Rothstein 2016; Schoen 2017; Tewari 2017; Valaitis 2005), because of being able to send information quicker (Chang 2011; Madon 2014), and getting immediate or faster feedback and support from peers (Barnabee 2014; Chang 2011; Huq 2014; Lodhia 2016; Madon 2014; Schoen 2017), higher‐level health workers (Ayiasi 2015; Medhanyie 2015), and laboratories, programme managers, and facility staff (Barnabee 2014; Chang 2011; Huq 2014; Madon 2014; Schoen 2017): "Now for me, a PHW with a phone, I can't be like that PHW [peer health workers] without a phone. His/her information cannot [travel] as fast as mine who has a phone" (Chang 2011). Speed efficiency also related to being able to quickly contact clients or vice versa (Cherrington 2015; Coetzee 2017; Hao 2015; Jennings 2013; Messinger 2017), particularly when this meant not having to travel (Ayiasi 2015; Barnabee 2014; Cherrington 2015; Hampshire 2016). Mobile health was also perceived to be more efficient because it improved workflow (Garg 2016; Praveen 2014; Rothstein 2016; Surka 2014), provided follow‐up reminders to health workers (Jones 2012; Rothstein 2016; Schoen 2017), and allowed them to provide immediate feedback to screened clients (Lodhia 2016; Watkins 2018). In one study, health workers were concerned that the ability to work faster because of mobile health was used by management to support staff cutbacks (Valaitis 2005). There were also health workers who experienced mobile health overall, or in part, as inefficient. This included perceptions of the technology as slow, time consuming, and increasing workload: "It complicates and increases our work. One has to stop everything she is doing and concentrate when sending reports" (Mwendwa 2016). The perceived increase in workload was in part because it was experienced as more cumbersome and taking longer to complete work (van der Wal 2016), compared to standard practise (Ginsburg 2016; Hao 2015; Kolltveit 2017; Mwendwa 2016), and sometimes because the application was slow (Schoen 2017). Mobile health also increased workloads when better screening procedures resulted in detecting more clients who needed treatment (Lodhia 2016).

**Finding 11: Health workers frequently reported mobile devices as overcoming the difficulties of rural and geographically challenging contexts when it made it possible for them to provide health care without having to travel. Some reported that reducing travel time allowed them more time with their clients** (high confidence in the evidence)

Health workers in rural and geographically challenging contexts appreciated the efficiency of mobile devices in allowing them to offer a service despite these circumstances, because it saved them travelling to clients and health facilities (Chang 2011; Hampshire 2016; Henry 2016; Lodhia 2016; Messinger 2017; Mwendwa 2016; Quinn 2013; Toda 2017): "...those who are in the most remote areas who have the highest prevalence for blindness will now be linked to the health system and so people will be able to find them and treat them" (Lodhia 2016). It was also used to schedule visits in advance which avoided wasteful travelling (Hampshire 2016). Some workers pointed out that finding clients in these contexts still required being provided with detailed client information on mobile devices (Rothstein 2016). Using these devices saved travelling time in an urban setting too, which allowed health workers to spend more time with their clients (Valaitis 2005).

**Finding 12: Health workers appreciated the portability and work schedule flexibility of mobile devices** (moderate confidence in the evidence)

Health workers had positive views and experiences about the portability of mobile devices. This allowed them flexible working hours, and allowed them to be less office‐bound (Hampshire 2016; Murray 2011; Nguyen 2015; Orchard 2014; Ramirez 2017; Schoen 2017; Toda 2017; Valaitis 2005; van der Wal 2016). Being able to carry mobile phones allowed them to access clients' records when visiting them (Murray 2011; Ramirez 2017; Schoen 2017; van der Wal 2016): "Another example, let's say a community member has a question about an appointment from a few months ago, like when it was. I can just pull it up right away!" (Ramirez 2017). In another study, school nurses expressed their appreciation of not being office‐bound as follows: "I was able to forward an email that had valuable information to my student, right from my school, and right to their home and office email. Otherwise, who knows when I could have sent [it]"; and "[mobile computing ‐ MC] does allow me freedom in my work. It helps to balance [the] workload, since I have access to files more frequently, therefore allowing me to work at times that in the past I was unable to... in particular while sitting in schools during a down period" (Valaitis 2005).

**Finding 13: Through mHealth, health workers were able to use treatment and screening algorithms that were loaded onto mobile devices. Their perceptions of using these electronic algorithms ranged from finding it easy and useful, to threatening their clinical competency, and an information overload. There were also some concerns that erroneous data entry may lead to wrong treatment guidance** (high confidence in the evidence)

Several mobile health programmes comprised of treatment and screening algorithms loaded onto the devices (Ginsburg 2016; Lodhia 2016; Orchard 2014; Ramirez 2017; Rothstein 2016; Shao 2015; Surka 2014; van der Wal 2016). In other instances, mobile devices were preloaded with the information that health workers had to share during client consultations (Ilozumba 2018; Mitchell 2012). Health workers often found it easy to integrate this into routine care (Ginsburg 2016; Lodhia 2016; Tewari 2017), and that it reduced inaccurate consultation and data capturing procedures (Ginsburg 2016; Ilozumba 2018; Mitchell 2012; Mwendwa 2016; Nguyen 2015; Orchard 2014; Shao 2015; Surka 2014; van der Wal 2016): "They [health workers] trusted the results of the mPneumonia algorithm, reporting that "the machine does not tell lies, it will rather tell you the right thing to do"" (Ginsburg 2016), and useful because it guided and simplified providing care (Ginsburg 2016; Mitchell 2012; Rothstein 2016; Shao 2015; van der Wal 2016), which workers experienced as reassuring and improving their knowledge (Ginsburg 2016; Ilozumba 2018; Lodhia 2016; Mitchell 2012; Orchard 2014; Rothstein 2016; Shao 2015; van der Wal 2016). Contrary to these experiences, other health workers held negative perceptions of using algorithms, as they felt it too prescriptive, and were concerned that they may lose their clinical competencies by blindly following it (Mitchell 2012; Surka 2014): “I enjoyed using the chart [paper‐based system] because you could check by yourself and see the status of the participant but when using the phone, it works out everything, it does not tell you what is wrong with the participant" (Surka 2014), and that it was too comprehensive and time consuming (Ginsburg 2016). In one study, health workers were concerned that caregivers would not understand, and by implication be dissatisfied, when the directive of the algorithm was counter to their treatment expectations (Mitchell 2012). There were also concerns that erroneous data entry may lead to wrong service reminders (Nguyen 2015).

**Finding 14: Using mobile devices to record routine client or surveillance data was mostly perceived by health workers and their managers as helpful for decision making, and increasing community and health worker appreciation of these data** (moderate confidence in the evidence)

The use of mobile devices to record routine client (Khan 2015; Rothstein 2016; Schoen 2017), and/or surveillance data (Lodhia 2016), was perceived by health workers and their managers as helpful for the continuity of care (Khan 2015; Madon 2014; Ramirez 2017), decision making and resource planning (Lodhia 2016; Madon 2014; Rothstein 2016), information sharing (Murray 2011; Nguyen 2015), and responding to disease outbreaks (Toda 2017): "At the district level, the majority of officers we interviewed were also pleased with the NTD MIS [software on the mobile phone to collect data at point of source] as reflected in the following comment made by one officer, "I can sit in my office and make a decision based on what is in the database"" (Madon 2014). In one study, the data sharing between different services was regarded as achieving a policy goal (Murray 2011). Workers reported specific advantages to data generated through mobile devices compared to paper‐based reports, which included that it was easy to format the reports to their needs, such as viewing individual or aggregated data (Nguyen 2015; Rothstein 2016; Schoen 2017), and having visual presentations of trends (Rothstein 2016). Mobile devices also offered continued access to data, which allowed timely adjustments of services in response to data trends (Lodhia 2016; Rothstein 2016; Schoen 2017; Toda 2017). Managers described automated visual presentation of data as important where workers struggled with analytical capacity, and the "ready cooked", graphical presentation then made for easier visualisation of what they needed to know (Rothstein 2016). A further advantage of using mobile technology for data collection was that it raised awareness of the value of data for decision making amongst community members (Nguyen 2015), and lower‐level health workers (Rothstein 2016): "Geohealth [mHealth intervention] … helps me make lists of community members in my area. I can choose, for example, to see only the pregnant women in my area, or only the 2 year olds, and then I have a complete list right away" (Rothstein 2016). One programme set automatic thresholds for accuracy and completeness (Rothstein 2016). If these were not met, then reports could not be generated, and this lack of generation of reports was described as frustrating (Rothstein 2016). Some workers suggested additional feedback mechanisms to counter delays in receiving data caused by disrupted connectivity, and recommended having dedicated staff for data capturing (Rothstein 2016).

**Finding 15: In most cases, health workers perceived mobile health as more advantageous than paper. However, some continued to prefer paper** (high confidence in the evidence)

The majority of health workers mentioned advantages to their use of mobile devices, compared to using paper‐based systems (Coetzee 2017; Madon 2014; Mitchell 2012; Nguyen 2015; Surka 2014; Toda 2017; Valaitis 2005; van der Wal 2016). The advantages included convenience (Nguyen 2015; Rothstein 2016; van der Wal 2016; Vedanthan 2015), quicker recording of their work (Ginsburg 2016; Mitchell 2012; Rothstein 2016; Valaitis 2005), easier access to client data (Nguyen 2015; Schoen 2017; Surka 2014; Vedanthan 2015), reducing and easy correction of recording mistakes (Madon 2014; Schoen 2017), faster error alerts (Nguyen 2015), not having to carry heavy paper stationery (Madon 2014; Schoen 2017): "In our work we walk a lot, and our backpacks are heavy, and we end up taking off our backpacks in community members' homes and forgetting them there. Biggest difference between Geohealth [mHealth programme] and paper? My bag is lighter with Geohealth! I don’t have to carry as much paper" (Schoen 2017), ease of moving through algorithms (Mitchell 2012), ease of information transmission and sharing (Mwendwa 2016; Rothstein 2016; Toda 2017; van der Wal 2016; Watkins 2018), immediate recording of data and therefore reducing of errors (Nguyen 2015), quicker access to summaries of reported data (Nguyen 2015; Rothstein 2016; Schoen 2017; Surka 2014), and a more durable platform compared to paper‐based systems, for example with papers that can be damaged by rain (Nguyen 2015; van der Wal 2016). For some workers, these benefits of mobile devices over paper‐based systems, as well as the perceived improvement of care, led to a commitment to use these devices (van der Wal 2016). In one study, being issued with mobile devices made health workers feel more professional compared to when they used a paper‐based system (Valaitis 2005). Yet, in one study, it was found that despite the mentioned advantages, most of the health workers did not use these devices during their visits to clients (Schoen 2017). Some workers preferred paper‐based systems, as they perceived these as safer to store information, more flexible, and had concerns about malfunctioning technology (Bacchus 2016; Schoen 2017, Valaitis 2005; van der Wal 2016): "One participant looked on it as "a totally negative thing, because I’m a paper person and I trust the paper in front of me. I do not lose paper"" (Valaitis 2005). These concerns resonated with those of health workers who complained that mobile health applications were slower than their paper‐based systems (Schoen 2017), and that it was easier to correct errors on paper forms because this did not require technical knowledge (Nguyen 2015).

**Finding 16: mHealth interventions sometimes required health workers to perform tasks that were peripheral to regular service delivery, such as registering clients onto the system. These more menial tasks were sometimes regarded as undermining to professional staff** (very low confidence in the evidence)

In some instances, the use of mobile devices to deliver or support healthcare services resulted in tasks that were additional to health workers' routine care practices. These tasks included registering clients onto the mobile health system (Hirsch‐Moverman 2017; Medhanyie 2015), and being called upon by clients to respond to technical problems or assist them with service plans for their devices (Murray 2015). Some workers perceived the additional tasks as menial and not appropriate for their job level: One respondent [health worker] commented that MomConnect registration [mHealth programme] was not seen as in line with the scope of professional work of a nurse: "The way I take it, it's undermining. Professionals don't do this, really." (Wolff‐Piggott 2018), and recommended outsourcing technical support for clients with technical and service plan problems (Murray 2015).

**Finding 17: Some health workers experienced the use of mHealth as generating an extra workload when, for instance, it resulted in reaching more clients needing care, or having to maintain both a mobile health ‐ and paper system. Some workers disliked this, particularly when their superiors did not perceive their mobile health work as part of their job description. Others did not object to the additional work, yet others wanted to be remunerated** (high confidence in the evidence)

There were several ways in which health workers perceived that mobile health programmes added to their routine healthcare services (Kolltveit 2017; Murray 2015). This included adding more steps to an existing mobile intervention (Chang 2011), inputting data (Mwendwa 2016), and reaching clients who may previously have been missed, but now because of being found created more demand on themselves and the health services (Rothstein 2016; Lodhia 2016). In one instance, the underlying values of the facility of building relationships with clients, created a greater demand on health workers' time, as they felt a need to immediately respond to text messages from clients (Murray 2015). Health workers held mixed views about this increased workload. Some held negative feelings when it complicated their work by maintaining two systems (Kolltveit 2017; Mwendwa 2016; Rothstein 2016; Shao 2015). They also disliked it when the addition of the mobile health interventions to current work was not understood and appreciated by supervisors: "… there were some accounts of hostility from the ANM [supervisor]: ...she (the ANM) scolded me and said that I am giving too much importance to this work...I said I am doing both jobs...and it is my problem...but I made 1 mistake...I regretted not showing this tablet to her" (Praveen 2014), or when they themselves perceived the intervention as peripheral to their work (Wolff‐Piggott 2018). Other health workers were neutral about maintaining paper‐based and electronic records (Mwendwa 2016; Schoen 2017): "… besides having to use the electronic devices, the registers had to be filled too "…yeah, it adds more work to us, because we have to enter the data in the phone, and then write the same data in the file, or patient's card/notebook." (Shao 2015). Some health workers did not object to the additional work (Hao 2015; Murray 2015), whilst others expected to be remunerated for the additional work (Hao 2015).

#### Theme 3: mHealth led to new forms of engagement and relationships with clients and communities

**Finding 18: Through mobile devices, health workers and clients could communicate directly with each other, which health workers reported as improving care and their relationship with clients. When clients initiated the contact, health workers felt that clients took ownership of their health. Health workers felt that some clients still warrant face‐to‐face contact** (moderate confidence in the evidence)

Mobile devices facilitated two‐way communication between all categories of health workers and clients (Chang 2011; Garg 2016; Hampshire 2016; Hirsch‐Moverman 2017), and kept health workers informed about the clients' conditions (Barnabee 2014; Huq 2014). Some workers perceived this to lead to immediacy of care (Barnabee 2014; van der Wal 2016), enabling follow‐up of missing clients (Hampshire 2016), informing care options (Barnabee 2014; Huq 2014; Messinger 2017), and advice and emotional support to clients (Cherrington 2015; Hampshire 2016), when physical contact was not possible. Health workers perceived direct communication with clients as facilitating a trusting relationship with clients (Murray 2015). It also allowed clients to initiate contact with health workers (Hampshire 2016; Jennings 2013; Messinger 2017; Murray 2015): "One woman, who came for [contraceptive implant] took my number. When she does not understand anything, she calls me for clarification" (Hampshire 2016). Workers perceived this as empowering clients to take responsibility for their health (Murray 2015). Some workers allowed the clients to lead the conversation during their communication, which enabled workers to tailor their services to clients' needs (Murray 2015). Mobile devices also allowed workers to contact clients’ relatives when needed (Jennings 2013; Lodhia 2016; Schoen 2017). However, it was felt that some instances still warrant face‐to‐face contact between worker and client (Jennings 2013; Messinger 2017): "Sometimes [counselling] can be taken through phone. But there are problems [with doing so]. Will it be right to give treatment without seeing the client?" (Messinger 2017).

**Finding 19: Health workers were aware of the importance of protecting confidential client information when using mobile devices, and the confidentiality risks in cases of stolen phones and using their SIM cards in colleagues' phones. Health workers were alert to clients' concerns when they shared personal information concerning stigmatised issues, such as HIV/AIDS and interpersonal violence, and suggested ways to keep the information confidential. They emphasised building a trusting relationship with clients prior to using the devices** (high confidence in the evidence)

Health workers were conscious of protecting clients' confidential information (Garg 2016; Hirsch‐Moverman 2017; Lodhia 2016; Murray 2015; Rothstein 2016; Valaitis 2005), complying with legislation regarding the protection of client information (Garg 2016), during consultations in which mobile devices were used. Workers were also aware of clients’ anxieties as to how their information will be used during mHealth‐facilitated consultations (Coetzee 2017), particularly when the information they provided concerned stigmatised and sensitive issues, such as HIV/AIDS and interpersonal violence (Bacchus 2016; Hirsch‐Moverman 2017; Wolff‐Piggott 2018), or showed clients in a bad light because of their behaviour: "Several CHWs reported that some clients worried that the devices were being used as voice or video recorders. "I thought that they [clients] were going to think that you will record them and take pictures of them. I thought they were going to say that as we [clients] drink this way […], you are now going to record us and take pictures of us"" (Coetzee 2017). A lack of legislative clarity on data protection caused some uncertainty for health workers in how to proceed with sharing information via text messages with clients (Garg 2016). Health workers were uncertain about probing clients on sensitive issues that these clients received information about through the device, but may not have wanted to talk about further with the health worker (Bacchus 2016). Their narratives suggest that this was a dilemma to their interpersonal interaction with the client (Bacchus 2016). In this study, mobile devices were used to prompt clients about their experiences of intimate partner violence, with some health workers perceiving these devices as a barrier to engage with clients, whilst others found it helpful (Bacchus 2016). Health workers perceived that some clients did not want sensitive information sent to their personal devices, as they were concerned about the transmission of the information and having it on the device where others may accidentally see it (Hirsch‐Moverman 2017). Measures to protect client information ranged from encryption (Lodhia 2016), using shared secret code words in text messages to clients (Hirsch‐Moverman 2017; Murray 2015): “I text him [client] and say, "It is your time now. Have you remembered your food?" He already knows. I will have taught him that when I say that, I mean it’s time to take his pills" (Hirsch‐Moverman 2017), and an explanation to clients on how their information will be protected (Lodhia 2016). Some health workers preferred sending automated text messaging to clients from a non‐traceable number, rather than using their personal phones to send these messages (Murray 2015). Other health workers pointed to the importance of building a trusting relationship with clients prior to using these devices, perceiving this as a way to mitigate clients' concerns about confidentiality (Coetzee 2017). Health workers were also concerned about risks to client information in cases of stolen phones, and when using their SIM card, with client information, in colleagues' phones (Lodhia 2016; Mwendwa 2016).

**Finding 20: Health workers were concerned that concentrating too much on the mobile technology during client consultations could be to the detriment of their service and interaction with clients** (low confidence in the evidence)

Some health workers were concerned that concentrating too much on the devices during client consultations could distract them to the detriment of their service and interaction with clients, particularly when it resulted in loosing eye contact and missing non‐verbal cues from clients (Bacchus 2016; Schoen 2017; Vedanthan 2015): "The patient likes to talk to you directly but you, you just concentrate in the gadget" (Vedanthan 2015). Some experienced clients as being dissatisfied with the loss of interaction and eye contact due to the workers concentrating too much on their devices (Schoen 2017; Vedanthan 2015): "The community members, especially the older ones, complain. They say, look at me! I'm telling you a story, pay attention!" (Schoen 2017).

**Finding 21: Health workers had differing reactions to being contactable via mobile devices during and outside of working hours: some felt it was useful, some were ambivalent about it, and others objected to it. Workers suggested setting boundaries to protect themselves from this** (moderate confidence in the evidence)

Clients with access to health workers contact numbers, were able to contact them at all hours (Cherrington 2015; Hampshire 2016). Some health workers perceived this as useful in emergency cases (Chang 2011; Hampshire 2016), some were ambivalent about it (Huq 2014), and others disliked it (Hampshire 2016): "Last year a patient called me at 5 am. I was deeply asleep when she called and said she was having menstrual pains and did not know what to do" (Hampshire 2016). Some wanted to protect their privacy by setting boundaries to protect themselves from working outside of working hours (Valaitis 2005), and did not give their contact details to clients (Hampshire 2016; Schoen 2017): "I don't give my WhatsApp number to my community members because I need a barrier between personal and work life—it doesn't work out to mix them. I have to cut it off somewhere" (Schoen 2017).

**Finding 22: Health workers experienced the use of mobile technology to provide health care, as being met with both trust and skepticism from clients and the communities they served. They described how trust or skepticism in the device was translated into trust or skepticism of their service when using the device. Some found that using mobile devices raised their social status with clients, and even their families. Others were concerned that using expensive equipment would emphasise inequity between themselves and clients** (high confidence in the evidence)

Health workers reported that mHealth devices raised their social status (Coetzee 2017), increased their recognition from family and friends (Madon 2014; van der Wal 2016), and increased the trust and respect they received from clients (Ayiasi 2015; Coetzee 2017; Ilozumba 2018; Lodhia 2016; van der Wal 2016): "Now we are famous because of our work. People listen to what I tell them, they respect us because we have brought services nearer to the people" (Ayiasi 2015). This was in part due to the connotations, such as prestige, innovation, and trustworthiness, attached to the devices, (Ginsburg 2016), the applications on the devices (Ginsburg 2016), and the purpose for which the devices were used (Coetzee 2017; Ginsburg 2016). These connotations were sometimes transferred to the health workers, such as them being perceived as trustworthy as they were entrusted with expensive and modern devices (Coetzee 2017; Ilozumba 2018; Madon 2014; van der Wal 2016), or because these devices made them more thorough (Barnabee 2014; Ilozumba 2018; Madon 2014; Mitchell 2012; van der Wal 2016). Being seen as a trustworthy health worker made clients more receptive to workers' use of mobile devices (Ilozumba 2018): "I am not telling. The mobile is telling. They will hear its words. Whoever is nearby they also become silent and will here all things" (Ilozumba 2018). Some health workers believed that using mobile devices helped them build a relationship with clients, and that satisfied clients told each other about the quality of care they received through mobile devices (Jones 2012; Khan 2015). This encouraged more clients to come and see them (Khan 2015). The elevated status, trust and respect were also linked to their use of these devices to access higher‐level care (Ayiasi 2015; Khan 2015; Mwendwa 2016), perceived by some clients as health workers showing care (Ayiasi 2015; Cherrington 2015), being credible (Ayiasi 2015), and being immediate in their support (Cherrington 2015). Health workers said that some clients were attracted from outside their catchment areas because of the mobile devices they used. Some clients not having the illness condition for which the health workers used the devices for, would question why they were not treated with these mobile devices (Vedanthan 2015). In contrast, there were health workers who experienced clients and communities as less responsive if they were sceptical as to the devices and how it can be used (Lodhia 2016; Mwendwa 2016): "Some mothers still do not know Rapid SMS and do not understand how useful the system is so they are uncooperative and do not give us information" (Mwendwa 2016). Workers suggested that this skepticism be addressed through community education (Lodhia 2016). There were also concerns amongst workers that using expensive devices working in resource‐constrained communities would emphasise the social inequity between clients and health workers, and would impact negatively on connecting with underprivileged clients (Valaitis 2005).

**Finding 23: Health workers experienced clients as having an opinion not only about their use of mobile devices, but as having an opinion on the devices themselves, which influenced how they responded to care delivered with the support of these devices. Health workers ascribed clients' enthusiasm for mobile devices as due to these clients' perception of the devices as prestigious, offering trustworthy information, and providing confidentiality. They perceived clients as more receptive when these clients were familiar with the devices used. There were concerns that clients who felt that the use of these devices during care was too time consuming, and would respond negatively to its use** (moderate confidence in the evidence)

Health workers felt that some clients were enthusiastic about the use of mobile devices in support of their care, as they perceived it as prestigious and modern (Lodhia 2016; Mitchell 2012; Valaitis 2005; Vedanthan 2015), offering credible information (Ginsburg 2016; Ilozumba 2018; van der Wal 2016), and made health workers more thorough (Mitchell 2012). Some workers described how it attracted more clients (Jones 2012; Messinger 2017; Vedanthan 2015), and clients trusted the accuracy of mobile devices, with some perceiving the addition of these devices during care, to be better than standard care (Ilozumba 2018; Khan 2015; Mitchell 2012; Shao 2015). Discontinuation of the use of mobile devices caused problems with new and returning clients, who expected it to be used (Mitchell 2012). Some workers perceived clients to be more willing to report sensitive information through mobile devices, than doing so verbally or on paper (Bacchus 2016; Westergaard 2017): "So if they feel safe enough to do it on the tablet, feeling like it's a little anonymous, it starts to break down those walls and maybe next time they'll want to talk about it" (Bacchus 2016). Workers also reported clients' preference for mobile devices when these offer non‐invasive medical diagnosis, as compared to invasive diagnosis, such as drawing blood (Ginsburg 2016). They also perceived clients to be more receptive to mobile health, if they were already familiar with the type of device used by the health workers (Garg 2016; Lodhia 2016). In contrast to the positive reactions, health workers were concerned that clients who perceived using mobile devices during care, as too time consuming, would respond negatively to such devices (Ginsburg 2016; van der Wal 2016): "The few negative reactions from clients related to them being impatient with the lengthy consultations. [Health worker]: Before, we mainly asked about danger signs but now we have many questions… some patients were impatient to answer all the questions" (van der Wal 2016). Other health workers reported that clients, particularly the elderly, disliked the use of mobile devices during consultations (Schoen 2017).

**Finding 24: Some interventions required clients to have phones as well as health workers. Health workers described this as challenging for multiple reasons, including clients not having phones, changing their phone numbers regularly, not knowing how to use a phone, being a target for crime because of possession of the phone, and women being prohibited from accessing phones. Health workers suggested competitive pricing to increase clients' accesses to phones, and to issue clients with phones** (moderate confidence in the evidence)

Health workers identified that interventions that required communication between health workers and clients, might pose several challenges to the clients. These included clients who regularly changed their phone numbers without informing the health worker (Hirsch‐Moverman 2017): "For others you find that the patient has given you a certain number, in a blink of an eye he has changed it without telling you that he doesn't use that number anymore" (Hirsch‐Moverman 2017); clients who did not have phones (Chang 2011; Tewari 2017; Wolff‐Piggott 2018), or did not always have their phones with them (Tewari 2017); clients who did not have money to buy phone credit or access to electricity (Huq 2014; Wolff‐Piggott 2018); and clients who were afraid of being robbed of their phones: "some of them …. they say they can't have these telephones because they come very early." It was explained that women queuing in the dark might be targets for criminals (Wolff‐Piggott 2018). There were also clients who did not know how to use mobile phones (Tewari 2017; Wolff‐Piggott 2018). In one case, women's access to mobile phones was prohibited as a consequence of gender discrimination (Hirsch‐Moverman 2017). Competitive pricing of mobile phones and phone credit costs increased clients' accesses to phones, and thus eased health worker ‐ client communication (van der Wal 2016). In one study, health workers reported that providing clients with mobile phones, promoted these clients' general social connectedness (Murray 2015).

**Finding 25: Health workers were ambivalent about interventions that required clients to use the health workers' mobile devices during consultations. Their optimism was tempered by concern that there was a loss of meaningful engagement with clients** (low confidence in the evidence)

Health workers had mixed reactions to clients using their (health workers') mobile devices on their own during consultations. In one instance, workers expressed that giving clients the device to access surveys and health promotion material on their own during the consultation allowed clients to deal with sensitive topics in private (Bacchus 2016). Yet, these same health workers were concerned about the loss of meaningful interactions during this process, and raised concerns that they were unable to engage with the clients about the topic at hand, unless the client was willing to share the activity with them: "The challenge is how to keep it personal. If [women] answer positive on the tablet and then you just close the tablet and "oh thank you" and put it away then you've just told her, all I needed was for you to answer the questions. I'm not really here to help you. You have to say okay so this is how you answered and this is how you scored, let's talk more about that. The computer can't do that part, all it can do is take down the information and it's up to the nurse or home visitor to expand upon it and actually get her the assistance that she needs" (Bacchus 2016). In another instance, health workers felt that having material on the device which they could share with clients was useful on days when they (the health workers) were tired, and not up to interacting with the client (Coetzee 2017). However, it has to be noted that in this study, the authors pointed out that they did not agree with the health workers' interpretation that using the device to show health promotion material when they were tired, was a good thing.

**Finding 26: Health workers reported that their access to mobile devices was beneficial to clients and communities who were too poor to own mobile phones** (very low confidence in the evidence)

Health workers reported that their access to mobile devices benefited clients and communities who were too poor to own mobile phones, or to afford paying local merchants for the occasional use of a mobile phone (Chang 2011). Health workers' access to a mobile phone enabled them to access higher‐level care, on behalf of these clients: "I was saying that you can find a whole village without a phone. So, giving these PHWs phones, is helping a lot… By giving these PHWs phones I think it helped them a lot because they just go and contact their PHW, and the PHW calls us" (van der Wal 2016).

**Finding 27: Health workers felt that health promotion and educational messaging directed at clients using mobile health interventions, impacted positively on clients' health behaviours, but cautioned against repetitive showing of health promotion videos. In one instance, issuing clients with mobile phones led to increased use of healthcare services** (moderate confidence in the evidence)

Health workers perceived mobile health interventions as useful for health promotion and education (Coetzee 2017; Lodhia 2016), and that it impacted positively on clients' health behaviours (Bacchus 2016; Barnabee 2014; Chang 2011; Ginsburg 2016; Huq 2014; Ilozumba 2018; Jones 2012; Madon 2014; Praveen 2014): "…people have been motivated to take the drugs because they know that once you get to their home and you press the phone to send a message it means that you are reporting the patient that he has not taken the pills properly….Patients are motivated now to take their pills" (Chang 2011). In one study, health workers thought that clients were motivated to improve their adherence because they were aware that information about their adherence was being relayed to clinic staff by field staff in real time via the mobile devices (Chang 2011). Some workers reported that clients were more responsive to visual material than verbal messages (Coetzee 2017; Lodhia 2016). Some attributed this to a cultural preference for visual information over verbal information (Coetzee 2017). Other health workers cautioned that repetitive showing of health promotion videos would not improve uptake of the health messages (Bacchus 2016; Coetzee 2017): "My client looked at me one time, and she said "how many more times do we have to do this?" (Coetzee 2017). Workers reported that issuing phones to clients for health‐related usage led to increased use of healthcare services (Murray 2015). Health workers perceived that graphic displays on a device helped clients to better understand their condition: "It's wonderful. I got better results than I expected...If patients see the risk bar, they understand very well that they have a high risk of CVD...We gained knowledge from this percentage display too...This is 100% beneficial to the doctor" (Praveen 2014).

#### Theme 4: Health workers' use and perceptions of mHealth could be influenced by factors tied to costs, the health worker, the technology, the health system and society, poor network access, and poor access to electricity

**Finding 28: Some health workers accepted bearing the costs of mHealth interventions themselves, but were dissatisfied when phone credit to use the phones was not delivered on time. Health workers felt that clients appreciated it when health workers called them, as it saved them costs** (high confidence in the evidence)

The cost implications for health workers, of mHealth was discussed across the studies, with differing opinions as to the appropriateness and affordability of bearing costs personally (Hampshire 2016; Messinger 2017; Watkins 2018; Wolff‐Piggott 2018). Bearing the costs personally was accepted by health workers either as part of their altruism (Hampshire 2016; Messinger 2017; Watkins 2018): "I know some nurses who will never use their own phones because they have no passion for the job. But, for some of us, it is the passion for the patients and the work that makes us continue" (Hampshire 2016); or from a sense that the investment would generate a greater demand for their services and thus better income (Khan 2015; Messinger 2017). There was less satisfaction with interventions that failed to deliver promised phone credit, on time (van der Wal 2016). Health workers also felt that clients appreciated it when the health workers saved them call costs by phoning them, rather than the other way round: "Even if any client calls me then I cut the line and call back from my phone. If I do so, client will say I am kind to them" (Messinger 2017).

**Finding 29: Health workers' digital literacy impacted on their experience and perceptions of the use of mobile devices in health service delivery: being digitally literate resulted in positive experiences and perceptions, whilst low digital literacy caused concerns about job security and embarrassment when making mistakes in front of clients. For some workers, prior exposure to mobile devices did not affect their perceptions and use of mobile health. Some turned their lack of digital literacy into building a relationship with clients by asking clients to show them how to use the devices. Not using the devices often enough, resulted in loss in digital literacy** (moderate confidence in the evidence)

The level of health workers' digital literacy, that is how comfortable they were in using mobile devices, shaped their perceptions and experiences of using mobile devices in their delivery of health care services (Cherrington 2015; Mitchell 2012; Murray 2011; Nguyen 2015; Praveen 2014; Quinn 2013; van der Wal 2016). Some health workers expressed initial hesitancy, but over time and with training, became more comfortable, to the point of expressing concern that they were becoming dependent on the intervention devices (Hirsch‐Moverman 2017; Mitchell 2012; Valaitis 2005). Health workers expected the training they received to alleviate their anxiety about using the technology (Coetzee 2017). Where health workers were able to use the application well, they regarded using it in service delivery (receiving results) as simple (Hao 2015). Health workers who had no or little digital literacy, initially did not know how to use the devices properly, and felt that they needed training to compensate for their lack of computer knowledge (Coetzee 2017; Hao 2015; Kolltveit 2017; Madon 2014; Murray 2011; Nguyen 2015; Praveen 2014; Quinn 2013; Shao 2015; Surka 2014; Valaitis 2005; van der Wal 2016; Watkins 2018). Such poor understanding of the technology could lead to dual systems, paper and electronic, and poor integration into normal routines (Murray 2011). Health workers, unfamiliar with the technology, were regarded by their trainers as needing emotional reassurance (Murray 2011). This emotional response was also seen in another study where health workers claimed to feel encouraged if sending a message worked, but feeling hopelessness if it did not (Mwendwa 2016). In one study, the authors expressed that those health workers that understood the mHealth application, expressed no concerns, but for other health workers who lacked this understanding, there was concern over errors in reading laboratory results (Hao 2015). A lack of ongoing training was said to lead to insecurity, and this insecurity in turn reduced enthusiasm for the intervention and hindered use of the device (Kolltveit 2017). Health workers across several studies suggested training as means to overcome poor digital literacy and unfamiliarity (Coetzee 2017; Kolltveit 2017; Murray 2011; Nguyen 2015; Praveen 2014). Accounts across several studies suggest unfamiliarity as the source of poor digital literacy, and that with familiarity came greater ease of use (Bacchus 2016; Coetzee 2017; Ilozumba 2018; Mitchell 2012; Quinn 2013; van der Wal 2016). In one study, where nurses already used the Internet or web applications on their phones for personal use, they were also more interested in gaining computer skills for their work, than those with no prior experience of using the Internet (Watkins 2018). It was not always clear where the difficulty stemmed from, faulty technology or unfamiliarity with the technology, but what was clear in one case, was that those who struggled, were the lowest educated (Ilozumba 2018). Poor digital literacy was not limited to health workers, but also to those with whom they had to engage, such as village leaders who expressed that they needed training because they were not able to understand reports and information given on the mobile phone (Madon 2014). One study suggested that irrespective of computer and smartphone literacy levels, that poor uptake was related to difficulty with typing, with health workers suggesting difficulty in use as a result (Shao 2015). Some health workers turned their digital illiteracy into an opportunity to build relationships with clients by asking clients to show them how to use the devices: "One of the older home visitors revealed that she used her own lack of experience with technology as a way of encouraging young women to open up to her with the computer tablet (it's like I'm saying you're really tech savvy with this and it's sort of like a prop you know. Like we're going to talk about this, but you get to use this tablet)" (Bacchus 2016). Others asked their children to assist them, which they viewed as appropriate given that there was no confidential information on the device (Coetzee 2017). It was reported that health workers with low client caseloads used the devices infrequently, and thus may forget how to use them (van der Wal 2016). In some instances, workers felt that poor competency with mobile devices threatened their job security (Madon 2014; Murray 2011): "At the same time, some VHWs were discriminated against for being unable to adapt to new ways of working as a result of the technology. For example, in one of the villages with low prior phone possession, some VHWs with visual impairments who had become used to entering health information onto paper registers were declared as being unfit to work because they could not easily operate the mobiles" (Madon 2014), and felt embarrassed when making mistakes whilst being with clients (Coetzee 2017). In one study while all doctors reported using the Internet at least once a week, the same was only true for a quarter of the nurses (Watkins 2018).

**Finding 30: Health workers expressed a need for training and familiarity with mobile devices to overcome their initial anxiety in using the devices. Peer training from technologically proficient colleagues was experienced as valuable. In several cases, health workers wanted refresher training and pointed to the importance of training replacement staff. Not having mentors who used mobile devices, impacted negatively on lower‐level workers' ability to learn how to use these devices** (high confidence in the evidence)

Some health workers experienced anxiety in understanding and using mobile devices, and felt that training and familiarity with these devices were needed to overcome this anxiety (Coetzee 2017; Ginsburg 2016; Hirsch‐Moverman 2017; Ilozumba 2018; Kolltveit 2017; Lodhia 2016; Mitchell 2012; Murray 2011; Mwendwa 2016; Nguyen 2015; Praveen 2014; Tewari 2017; Toda 2017; van der Wal 2016; Vedanthan 2015). In one study (Ilozumba 2018), younger health workers preferred group training and the older ones, individual training. In another study, the preference was for small group training (van der Wal 2016), and in yet another study, workers referred to the usefulness of peer training from colleagues who were proficient mobile health users: "... there are those who are sharp with the tablet they can show some of us things...So maybe during break you find someone they explain to you" (Vedanthan 2015). Health workers raised concerns about inadequate and too short training (Coetzee 2017; Mwendwa 2016; Toda 2017), which would lead to mistakes in using mobile devices (Coetzee 2017). In one instance, they reported receiving a detailed training manual in addition to in‐person training (Rothstein 2016). Some health workers recommended refresher training (Madon 2014; van der Wal 2016; Vedanthan 2015), and training replacement staff: "Ok if you look at a facility where we have only one nurse, and she was the one that was trained and she is being transferred, who else would. . . still be there to really continue?" (Toda 2017). Lacking refresher training made some health workers feel insecure and less inclined to use mobile devices (Kolltveit 2017). Some health workers felt hampered in learning to use mobile devices when it was not also used by their clinical mentors (Vedanthan 2015).

**Finding 31: All categories of health workers required technical support to solve user problems. At times, face‐to‐face support was provided, but technical support from proficient colleagues was useful too. Having technical problems solved through real‐time improvements worked well for some health workers, while others suggested a help function be added to the devices** (high confidence in the evidence)

Health workers, lay and professional, required technical support, face‐to‐face or telephonically when having difficulties in navigating mobile devices (Hao 2015; Ilozumba 2018; Kolltveit 2017; Lodhia 2016; Mwendwa 2016; Toda 2017; van der Wal 2016): "He [the supervisor] goes to their home, and asks them to sit on the chair and then he tells them to type to the people. He tells us [CHWs] to type like you do in front of me. Whatever you have a problem I am here for it. You do the typing" (Ilozumba 2018). The absence of technical support, created problems and frustration (Murray 2011; Rothstein 2016, van der Wal 2016). Health workers reported that they had technical problems solved through real‐time improvements and regular meetings (Cherrington 2015). In some instances, higher‐level staff provided technical support, and in other instances, peer‐to‐peer support (Garg 2016; Hao 2015; Madon 2014; van der Wal 2016), helped to solve technical problems. Workers felt that a help function on the devices would be useful to solve technical problems: [Health worker] "The error message is just a text. I can't call anyone for troubleshooting… it would be better to have a troubleshooting function in the application that suggests what to do when you receive an error message" (van der Wal 2016).

**Finding 32: Health workers highlighted that mobile technology applications should be user‐friendly, easy to learn, and improve the quality of their care. When the applications were not easy to use, health workers became frustrated and reluctant users of mobile devices** (high confidence in the evidence)

It was important to health workers that the software and applications on the mobile devices were easy to use (Ginsburg 2016; Khan 2015; Kolltveit 2017; Lodhia 2016; Praveen 2014; Rothstein 2016). In one study they appreciated multiple applications on their devices (Lodhia 2016). When the applications were easy to use, little training was required (Ginsburg 2016; Kolltveit 2017): "The simple design was "not difficult to use. . . [Y]ou just read and follow the instructions." Almost 80% of the HCPs said it took them a day or less to be familiar with the device and application when they used it for the first time during the training sessions" (Ginsburg 2016). Health workers also pointed to the importance of these devices improving the quality of their care (Ginsburg 2016; Khan 2015). Applications and software that were not user‐friendly, frustrated health workers and made some reluctant to use mobile devices (Kolltveit 2017; Ramirez 2017; Rothstein 2016; Schoen 2017). Some workers complained about slow, in one instance "painfully slow" (Ramirez 2017), and unreliable applications (Mwendwa 2016; Ramirez 2017).

**Finding 33: Health workers held mixed views on choosing between tablets and smartphones. Some felt that the type of content on the device was more important than the device itself. However, other health workers preferred tablets over smartphones, mainly because the bigger size of the screen was perceived as easier for client engagement** (very low confidence in the evidence)

In one study, half of the health workers were given smartphones and the other half were given tablets (Shao 2015). Their opinion about the two different tools were reported as being similar, showing more concern for the algorithm loaded on the devices, than the devices themselves (Shao 2015). In contrast, workers in another study who were issued with mobile phones, thought that tablets would have been better, because it would have made it easier to show clients what they were doing on the devices, and that it looked more professional (Schoen 2017).

**Finding 34: Some health workers felt that sustainable, at scale mHealth programmes required approval and stewardship from political leaders, such as ministries of health. Leadership interest in mHealth interventions was described as motivating to health workers. Health workers suggested that such leaders should be engaged early and continuously throughout the programme, and be provided with evidence of effectiveness, so as to secure their support. The lack of high‐level stewardship impacted negatively on the mHealth programme** (low confidence in the evidence)

Some health workers felt motivated when political interest was shown in mobile health programmes: "Even where healthcare professionals had to organize and manage the telemedicine use on their own, they could still feel some support from leaders when they experienced their attitudes toward telemedicine to be positive. [Health worker]: "There is an interest for this intervention by the leaders, but they are not so interested in knowing more about the intervention. My leader is pleased with the fact that I am handling it all" (Kolltveit 2017). They perceived that sustainable, at scale use of mobile devices required approval and stewardship from higher‐level leaders, including decision makers at national ministries of health (Ginsburg 2016; Kolltveit 2017; Lodhia 2016; van der Wal 2016). They felt that early and continued engagement with these leaders facilitated their support (Ginsburg 2016). Workers also reported that not including higher‐level professionals in programmes, affected intervention uptake (Kolltveit 2017). Receiving mobile devices and the means to use it, was perceived by some health workers as an acknowledgement of their work (van der Wal 2016). They also mentioned that evidence of effectiveness and cost‐effectiveness were important to ensure higher‐level support for mobile health programmes (Lodhia 2016). When there was lack of higher‐level stewardship, health workers felt it impacted negatively on the mobile health programme (Kolltveit 2017).

**Finding 35: Health worker accounts pointed to the strong influence of the health systems and social context in which the intervention was embedded. Contextual and systems issues, such as difference in language use between clients and health workers, gender discrimination, discomfort with professional hierarchies, poverty, resource constraints, staff attrition, and more, all of which were external to the technology and the physical device, influenced how health workers experienced mHealth and the use of mobile devices for service delivery, in their different contexts** (moderate confidence in the evidence)

Health workers' accounts showed that the systems in which mobile health programmes were implemented, and contextual issues external to the devices itself, shaped their experiences and perceptions regarding their use of the devices (Wolff‐Piggott 2018). These included language differences between workers and clients, which made communication difficult irrespective of using mobile devices or not (Khan 2015); cultural practices, such as gender discrimination against female use of mobile phones (Huq 2014); and educational and professional differences which caused strained relationships between lower‐ and higher‐level workers: "Two village doctors reported that the lack of comfort with the call centre doctors resulted in their reluctance to use the intervention. As one village doctor said, "When I talk with my patient I feel like we are brothers during our conversation and the patient feels comfortable (about sharing problems). But when I consult with call centre sometimes I didn't feel that warmth probably because they are from another place and we never met [sic]"" (Khan 2015. Furthermore, health workers felt that client poverty made the uptake of mobile health services challenging for clients if it required that they access a mobile device themselves, (Huq 2014; Tewari 2017). Staff attrition and shortages were often reported as main barriers to optimal implementation of the intervention, and uptake of the use of the devices as an additional activity, on top of existing staffing problems (Chang 2011; Kolltveit 2017; Lodhia 2016; Rothstein 2016; Shao 2015; Toda 2017): "In non‐urban settings, like this place, staff attrition is very high. . . . For the past about three years, we've been receiving an average of about 20 community health nurses every year. . . . These are new people. So they don't know anything about MOTECH (mHealth programme]*.* They have to be trained" (Rothstein 2016). The same applied to workers who reported that unsupportive higher‐level professionals made them less enthusiastic about mobile health (Praveen 2014; van der Wal 2016). Workers also found it challenging to learn how to use the new devices amidst the rush and unpredictable workday routines in primary healthcare facilities (Rothstein 2016). They also pointed out that other health systems' problems, such as when drugs were out of stock (Shao 2015), overstretched laboratory services (Shao 2015), and health workers not knowing pre‐mHealth reporting guidelines (Toda 2017), made it difficult to use mobile devices. In one disease surveillance programme which used mobile devices to record and report the surveillance data, staff felt that face‐to‐face supervision was an important enabler for high‐quality data, but resource constraints and poor road conditions made such supervision impossible, and that this lack of face‐to‐face supervision, not the technology, impacted negatively on the quality of the fieldwork staff (Toda 2017).

**Finding 36: It was important for health workers that mobile health interventions be integrated with other existing electronic health information systems. This interoperability made it more likely that mobile devices would be integrated into standard care practices, while the absence of integration frustrated health workers** (moderate confidence in the evidence)

Health workers felt it important to integrate mobile health interventions with other existing electronic health information systems (Garg 2016; Ginsburg 2016; Lodhia 2016). They appreciated it when integration worked well (Garg 2016; Rothstein 2016): "The cancellation that the patient is not [coming to their visit] – it's connected to the EHR or EPM [mHealth application] side of [this], so that automatically releases an appointment. Nobody has to do anything…which is gold. So that's automated" (Garg 2016), but struggled in the absence thereof (Lodhia 2016; Rothstein 2016). In one instance, where integration only happened at the district level, health workers suggested national government support for national level integration (Lodhia 2016). Health workers perceived having access to client information across different care points within the health system as a benefit of integration (Garg 2016; Rothstein 2016), and this made them enthusiastic to integrate their use of mobile devices into their routine workflow (Rothstein 2016).

**Finding 37: Health workers offered programmatic and implementation recommendations to improve mobile health interventions. The most cited of these was that the interventions be expanded to other settings and services, beyond what they were using it for as described in the studies. Other recommendations included raising community awareness about mHealth programmes, being involved in developing programmes, and appointing a 'mobile health champion'. Workers also suggested that those collecting surveillance data, must be informed of how the data are used** (moderate confidence in the evidence)

Health workers felt that mobile health interventions could be improved, and offered a range of recommendations related to programmatic and implementation issues. They suggested that mobile health programmes be implemented at scale, and expanded to other settings, services, and illnesses beyond what they were using it for, as described in the studies (Bacchus 2016; Medhanyie 2015; Mitchell 2012; Rothstein 2016; Toda 2017; van der Wal 2016). Across several studies, health workers suggested improving the social marketing of these programmes, including advertising and sharing information with users, clients, and communities, as this may enhance the acceptability and uptake of these programmes (Ginsburg 2016; Khan 2015; Lodhia 2016; Mwendwa 2016). They also recommended that those using the devices be consulted as end users during the planning and implementation of the intervention: "I [supervisor] think there needs to be more involvement of other people (users), to know, what do they think, how can this be done better, what are their inputs rather than pushing it down and ask them just to use the system" (Hao 2015); be included in decisions about system changes to be introduced because of the mobile health programme (Hao 2015); be given money for phone credit (Barnabee 2014); and that all paper‐based stationery, not just certain documents, made available on the mobile devices (Schoen 2017). Some recommended an automated response service for when health workers are in emergency situations (Mwendwa 2016). Workers advocated for the appointment of a 'champion' who could provide technical and intervention assistance (Kolltveit 2017; Murray 2015): "The importance of having a colleague who could champion this intervention was described as a prominent success condition: "Well, we have some among us who facilitate it all. They have encouraged us, and when some of us think this is too much work, they have been there with their enthusiasm. This enthusiasm has been valuable to us"" (Kolltveit 2017). There were recommendations that those collecting routine data using mobile devices, should be informed as to how the data are put to use, and that not getting this feedback made them feel like students who sat an exam without getting their results (Madon 2014).

**Finding 38: Health workers had several technical recommendations to improve mobile health devices, for instance solar panels to counter poor electricity access and using photos to track clients' recovery from illness. Other recommendations included using sturdier devices, bigger screens, and having common applications, such as work scheduling on the devices** (moderate confidence in the evidence)

Health workers suggested ways to improve the technical aspects of mobile health interventions. Password protection was proposed to keep client information confidential, installing tracking software to mitigate loss or theft of a device, and having theft alert protocols put in place (Coetzee 2017). Issuing solar panels (Henry 2016), backing‐up data (Ramirez 2017), and using battery‐powered systems (Lodhia 2016), were offered as possible solutions to the problem of access to electricity and power shortages. Some workers thought that taking photos of clients' improvement or participation in health promotion activities, could serve as encouragement to clients: "We [health workers] could also use it to motivate community members by showing them how they are improving. For example, if a community member [client] was taking good care of a wound but didn't think it was healing, we could show them a photo from last week and say "look here! It is better" (Schoen 2017). Other workers suggested sturdier devices: "… and it [mobile phone] [needs to be] a much tougher thing, it's going to be slapped around from my dressing table in the car, the houses, anywhere, the iPhones tends to be a lot more delicate …" (Quinn 2013), and features for easier user interface on the devices (Praveen 2014; Rothstein 2016). Applications commonly found in some studies, such as work scheduling, receiving reminders, and recording work done, were also recommended by those who did not have access to these features (Schoen 2017). There were also health workers who recommended using a stylus, i.e. a small pen‐shaped instrument, when typing on a tablet (Schoen 2017). In one instance workers thought that tablets, with their bigger screens, would make it easier for clients to see what the workers were doing on their devices, compared to small screen mobile phones (Schoen 2017).

**Finding 39: The main challenges health workers experienced in using mobile devices, were poor network connectivity, access to electricity, and the costs to recharge devices. Solutions offered, included using solar panels, using the powered‐up phone of a colleague, or reverting back to the paper‐based system. Sometimes poor connectivity resulted in client dissatisfaction because it created delays in receiving health care. Health workers' commitment to their clients motivated them to cope with these and other challenges** (high confidence in the evidence)

The most cited challenges for health workers using mobile devices were poor network connectivity (Hampshire 2016; Ilozumba 2018; Khan 2015; Lodhia 2016; Madon 2014; Nguyen 2015; Praveen 2014; Quinn 2013; Rothstein 2016; Schoen 2017; Toda 2017; van der Wal 2016), no easy access to electricity to charge their devices (Chang 2011; Ginsburg 2016; Hampshire 2016; Lodhia 2016; Madon 2014; Mwendwa 2016; Nguyen 2015; Schoen 2017; Toda 2017), and the costs to have their mobile devices charged (Hampshire 2016; Mwendwa 2016; Toda 2017). The challenge of charging was not only inconvenient, it could also impact on fully implementing the intervention. In one study it served as a deterrent to downloading the intervention software application (Ginsburg 2016). Poor connectivity impacted on clients, who became impatient in waiting to connect to a doctor or waiting to get a text message prescription (Khan 2015). To circumvent these challenges, workers used private solar panels (van der Wal 2016), though some complained that it took a long time to charge their devices (van der Wal 2016). In other instances, health workers solved poor connectivity by uploading their work at times when connectivity was good, but that may have been inconvenient to them (van der Wal 2016), or by looking for places with good connectivity: "We also have problems with network connectivity. For example, so the uploading may be a challenge. And sometimes they're typed, but it doesn't go through. Sometimes it doesn't go through at all. You have to go and climb a tree" (Rothstein 2016). Some had to walk long distances in search of good reception and/or electricity (Ginsburg 2016; Madon 2014; Nguyen 2015; Rothstein 2016). Some workers used their own resources to ensure connectivity (Quinn 2013; Watkins 2018), whilst others reverted back to the paper‐based systems when the mobile devices did not work (Nguyen 2015; Schoen 2017). Given problematic access to electricity, workers at times had to use the powered‐up phone of a colleague or otherwise put their SIM card into a colleague's powered‐up phone (Mwendwa 2016). In one case they used informal business networks to have their phones charged at a low cost (Chang 2011). Poor connectivity did not just challenge health workers, it challenged the success of the intervention being conducted as intended. It resulted in delays in providing health care (Quinn 2013), which upset some clients (Khan 2015), it caused complaints from workers that work was lost (Schoen 2017), devices 'froze' whilst they were working (Schoen 2017), and resulted in slow transmission and receiving of information (Schoen 2017; van der Wal 2016). However, health workers' commitment to their clients motivated them to cope with these and other challenges: " … [despite] feeling discouraged because of lack of Internet connectivity, they [health workers] kept stressing that the mHealth tools were in their community's best interest, good for their health system, and therefore felt a professional duty to accept and use the application. [Health worker]: "Our priority is mother and child care, so it is important that we fully succeed, isn't it?"" (van der Wal 2016).

**Finding 40: Health workers expressed dissatisfaction with mobile devices when technology changes were too rapid, showed a dislike for typing, and were concerned that mHealth impersonalised their interaction with clients. Since these dissatisfactions were only infrequently raised within the data set, it is unclear if these perceptions reflect wider experience** (low confidence in the evidence)

Some studies offered challenges raised by health workers that were not commonly discussed across the included studies. Health workers expressed dissatisfaction with aspects of mobile devices, which included when technology changes were too rapidly introduced (Valaitis 2005), or when their expectations of the devices were not met, for example when they anticipated it would make manual data capturing unnecessary, yet they still had to do it (Hao 2015), or not being able to record two visits simultaneously, particularly because it took too long to load the application for each new visit (Schoen 2017). There were also health workers who did not like typing on a mobile device (Schoen 2017). Other health workers felt that the devices at times impersonalised their interactions with clients when they required emotional support: [Home visitor]: "It's [tablet] cold…it’s just her interacting with a machine. So there's no sympathy, there's no condolences. There's no, I want to say loving interaction. No, um, it’s like no comfort, no support you know" (Schoen 2017). The infrequency with which these were raised, makes it unclear if these are widely‐held perceptions, reflecting a more general or broader experience.

**Finding 41: Health workers discussed challenges, beyond network and electricity issues, that sometimes were just an annoyance or a concern, but at other times also impeded their mHealth activities, and their ability to provide a service assisted by the use of mobile devices. These included damaged devices, loss and theft of devices, having to carry two devices, not being able to readily buy phone credit when needed, not being able to send long messages because of character limitations, and the limitations of the language capabilities of their devices** (moderate confidence in the evidence)

Apart from challenges with network connectivity and electricity issues, health workers listed a number of other challenges they faced when using mobile devices. These challenges included lost and damaged devices (Murray 2015; Schoen 2017), working in high‐crime areas and subsequently facing personal safety risks and risks of stolen devices (Chang 2011; Coetzee 2017; Schoen 2017; Toda 2017; Valaitis 2005). CHWs agreed that they were concerned about the safety and use of tablets in some of the communities and households they visited: "I was also afraid because of the places that I go to. The places that I go to criminals will be looking at me while they did not mind me before" (Coetzee 2017); workers' low proficiency with English and the unavailability of local language characters (Ilozumba 2018; Medhanyie 2015; Mwendwa 2016); character limitations for text messages (Hao 2015); small screens and keypads (Rothstein 2016; Schoen 2017; Valaitis 2005); old devices (Mwendwa 2016), problems with short‐life batteries (Praveen 2014; Quinn 2013; Schoen 2017; van der Wal 2016): "And the battery time was a bugbear with me. I hadn't got the time to be plugging it in everyday, I hadn't always got an office to plug it in" (Quinn 2013). It was also a concern when there were not enough devices (Lodhia 2016), and when they had to carry both a personal and work phone: "Many of the health workers complained about carrying two phones, the smartphone we gave them and their private phone. As a result, many of them stopped carrying their private phone and started using the smartphone as their primary phone" (Medhanyie 2015). Some workers complained when they lost work because it was not backed‐up (Cherrington 2015). In one instance, not having a vendor to purchase phone credit from, was perceived as a barrier to use mobile devices (Hampshire 2016), and in another, not having someone to repair their devices (Murray 2015; Schoen 2017). When health workers in one intervention uploaded photos, videos and music (sometimes private), it slowed the technology and disabled the mHealth application that they had to use (van der Wal 2016). These challenges were not simply an annoyance or irritation, they also impeded health workers in their service delivery. For example, one health worker explained that she/he could not call an ambulance for a violent psychiatric patient because she/he did not have credit on their phone and all shops were closed, so no credit could be purchased (Hampshire 2016). Character limitations in text messages prevented some health workers from sending required lab results (Hao 2015). Device shortage in combination with network and Internet coverage made for difficulties in sending images, client information, and for seeking advice (Lodhia 2016).

**Finding 42: Health workers complained when the tasks asked of them in mHealth interventions were felt to be beyond their clinical capacity, and when support from higher‐level workers was absent** (very low confidence in the evidence)

Health workers found it difficult to communicate or explain information to the client provided to them via their mobile devices when this information was beyond their clinical capacity. Examples of this included receptionists who had to screen clients for cardiovascular illnesses and relay the results to the clients: "Receptionists were unsure how to respond to patients' questions and generally felt this duty was not part of their role. They did not see the relevance of screening for stroke prevention. Patients would say, "Is this my heart rate?" and I would say, "I don't really know"" (Orchard 2014). Another example was lay health workers who preferred to refer screened clients to a doctor to receive their screening results (Praveen 2014). This difficulty was also expressed when there was an absence of higher‐level support in following up on the screening results (Praveen 2014).

### Results of linking the review findings to the Cochrane intervention reviews

As described in the Methods section, our review was used alongside several Cochrane intervention Reviews on the effectiveness of mHealth interventions, commissioned by the WHO to inform their guidelines on digital interventions for health system strengthening (WHO 2019). These reviews assessed the effectiveness of diverse types of mobile devices used by health workers to improve their delivery of care. These included mobile devices for birth and death notification (Vasudevan 2018), stock notification (Agarwal 2018a), client to provider and provider to provider telemedicine (Gonçalves‐Bradley 2018a; Gonçalves‐Bradley 2018b, tracking of clients' health status (Agarwal 2018b), and decision support (Agarwal 2018c). The findings from our qualitative evidence synthesis were used as a source of information about intervention acceptability and feasibility, which the WHO's guideline panel used as a basis for their recommendations. The GRADE evidence‐to‐decision tables where this evidence is presented alongside evidence from the relevant Cochrane Reviews of effectiveness are available in the Guidelines appendices (www.who.int/reproductivehealth/publications/digital‐interventions‐health‐system‐strengthening).

In addition, we also planned to develop hypotheses about why some mHealth technologies are more effective than others. After an assessment of the results from the reviews of effectiveness, as well as of our own review findings, we chose, however, not to develop specific hypotheses about individual mHealth technologies. One reason for this was that the preliminary findings of the reviews of effectiveness (apps.who.int/iris/bitstream/handle/10665/311980/WHO‐RHR‐19.10), showed large evidence gaps, while the evidence that was found was often of low or very low certainty. While there was some evidence of impacts on health outcomes, the available evidence suggested that these types of interventions may make little or no difference to the outcomes that were measured. While we do not currently have a clear picture of whether some mHealth technologies are more effective than others, the findings from our own review suggest that it is unrealistic to expect consistent positive outcomes in mobile health programmes. This includes outcomes tied to the health of clients, to service delivery and to the organisation of care. The qualitative evidence in our own review illustrates how these programmes are comprised of many interlinking and at times complex, components, for instance, health system arrangements whereby the programme is being implemented. Each of these components offer different issues that may contribute to positive outcomes, but also pose definite challenges that render mHealth susceptible to poor outcomes. These facilitators and barriers relate to, amongst others, (i) the devices and technology itself, for example, short‐life batteries and user‐friendly software (Findings 32, 41); (ii) health workers' aptitude for mobile devices and their digital literacy (Findings 15, 24, 29); and (iii) upstream issues such as health system arrangements and high‐level stewardship (Findings 34, 35). Therefore, we consider the following two key hypotheses based on our findings, may help explain and understand the effectiveness of mHealth programmes in delivering and supporting primary healthcare services.

There are self‐evident benefits to mHealth, for example linking people living in rural areas to forms of health care that they would never otherwise have had access to (Findings 1, 2, 22), it eliminates inhibiting transport challenges (Finding 11), access to real‐time data (Findings 5, 10, 14), and its portability allows health workers to access healthcare information at the point‐of‐care (Findings 9, 12). However, seeking and delivering health care, regardless of the mode, remains a relational transaction between a client and a health worker. Our findings show that all of the usual relationship issues that make health worker‐client relationships successful or challenging, issues such as being trustworthy, being seen to offer high‐quality care, being seen to be knowledgeable about the condition being treated, remain. However, with the addition of mHealth, this becomes complicated when for example, a health worker's trustworthiness may be determined by the sense of trustworthiness of the information they relay from the mobile device, or a health worker's status is elevated because they are seen as being worthy enough of being entrusted by their employer with an expensive device. The existing relationship complexities, therefore interact with the new relationship complexities brought by mHealth. The use of a mobile device as part of the transactional relationship between health workers and clients is therefore, but one element of a complex relationship, and understanding the eventual health outcome achieved, requires acknowledgement of this complexity, and acceptance that there may be factors which cannot be controlled for or easily explained. However, a health worker who is already regarded as trustworthy, and is able to show extra competence through use of a mobile device, is likely to be even more highly regarded, and the message they deliver through the use of the device may be regarded as more credible. The opposite however, is equally likely, a previously well‐regarded health worker, using a faulty device, may lose face with clients, and so may begin to lose credibility, and struggle to convince clients to adopt the behaviour that they are suggesting. The outcome therefore is sensitive to the interaction between these multilayered, complex factors and interactions.

No matter the sophistication of the mobile health technology or programme, if it is implemented in a health system that has challenges, then the mHealth programme is also likely to be challenged. In those instances, it is the dysfunctional system, rather than the device, technology, or programme, which becomes the barrier to positive effectiveness outcomes. Mobile health programmes are embedded in larger systems, and therefore impacted by contextual issues external to the technology itself, and how health workers use mobile devices. This was evident in health workers' reporting how health system and contextual factors impacted on their optimal use of the devices (Findings 3, 16, 34, 35, 39). Examples of these factors include network connectivity, access to electricity, staff shortages, unresponsive emergency services, inadequate supervision, and strained relationships between lower‐ and higher‐level health workers. It is our contention that the reverse also holds true: mHealth can have a positive effect when used within an already functional system and can sometimes close a specific gap in a system that is not fully functional.

In summary, the complexities of healthcare delivery and of human interactions defy drafting simplistic hypotheses that can predict the effectiveness of mHealth programmes to provide and support primary healthcare services. The effectiveness of these programmes results from the interplay between technology, context, and the human attributes of clients and health workers. Detailed programmatic and process description information and realist evaluations (Pawson 2001), alongside effectiveness studies will be a starting point to unravel this interplay and formulate hypotheses regarding the effectiveness of mobile health. Below, we offer a few recommendations regarding implementation practices that may improve the likelihood of positive outcomes when using mobile devices to provide and support the delivery of primary healthcare services.

### Review author reflexivity

We described our initial positioning earlier (see 'Review author reflexivity' in the 'Methods' section above). Our views remained the same during the review, though our continued team discussions led to more nuanced definitions regarding mobile health, whether it is used to deliver or support healthcare services, and primary health care. Whilst writing the 'Discussion' and the 'Conclusions', we were particularly aware of the risk of overlooking data that refuted our own experiences that mobile health intervention outcomes are usually a mix of having positive and no effects.

## Discussion

### Summary of main findings

For a summary of the main findings, please see the 'Plain Language Summary'.

### Overall completeness and applicability of the evidence

The majority of studies (74%) on which our findings are based, were conducted in low‐ or middle‐income countries. Most of these were from Africa (71%) while only one study was from Latin America (Brazil). Of the 12 studies conducted in high‐income countries, five were conducted in the USA, two in Canada, and one each in Australia, Ireland, Norway and Scotland. We downgraded our confidence in several of our findings because of the limited range of settings that the data were from.

Regarding the health issues addressed in the mobile health programmes, our data are a fair representation of the main services offered in primary health care in the study countries. Seventeen studies focused on maternal, neonatal and child health, four addressed HIV/AIDS, and two, malaria and tropical diseases. Other health issues included, amongst others, cardiovascular diseases, intimate partner violence, eye care, hypertension, wound care, and mental health. Similarly, there was an even spread of studies reporting on lay and professional health workers' experiences and perceptions: 10 studies reported on lay health workers, 12 on health professionals, and in 17, both categories were reported on. The health worker category was unclear in the remaining four studies.

We would also argue that the included studies mirror by and large, current device and application use, with tablets and personal digital assistants (PDAs) less used compared to mobile phones, most likely due to costs and convenience. Tablets and PDAs were used in only 11 studies (26%), and mobile phones, identified as iPhones or smartphones, in 31 studies (72%). In one study, both tablets and mobile phones were used. Device applications ranged from text messaging, screening and diagnostic algorithms, and pre‐loaded health promotion materials, to the recording of surveillance data and work scheduling.

Studies exploring healthcare workers’ use of digital health strategies are now published increasingly often. We have listed 85 studies under “Studies awaiting classification” that appear relevant for our review and that have been published since we finalised our analysis. In our next update of this review, we will sample from these studies, focusing in particular on those findings that we have downgraded using the GRADE‐CERQual approach.

### Comparison with other studies or reviews and implications for the field

A mixed‐methods review by Konttila and colleagues (Konttila 2019), on key competencies required from healthcare professionals to use digital technology, and the organisational factors that shape their use of it, resonates with several of our findings. They reported that using digital devices impacted both positively and negatively on the health worker‐client relationship (Findings 18, 22); led to concerns amongst health workers that they might lose their clinical competencies (Finding 13); were influenced by the users' level of digital literacy (Finding 29); and required refresher training and technical support (Findings 30, 31). Importantly, these authors echoed our views that digital technology is embedded within larger systems that can either advance or undermine the effectiveness of mobile health programmes (Konttila 2019).

We concur with Dunn and colleagues, that primary research is needed to understand the pathways of how mobile health effects change (Dunn 2018). It is clear from our findings that the effectiveness of mobile devices cannot be separated from those who use it to deliver health care. Mobile health interventions are a combination of device and health worker, each with unique attributes, but also jointly, that impact clients' health behaviours, and how primary health care is supported and delivered. Though our review reported on health workers perceptions of how clients perceived them and the devices they use, asking clients themselves may further our understanding of the pathways of effect. We are in agreement with Lee et al (Lee 2016), that clearer descriptions of the mobile health interventions may help to better understand its impact. Contrary to Marcolino's review of effectiveness reviews (Marcolino 2018), where it was found that the majority of studies were conducted in high‐income countries, 74% of the studies in our review were conducted in low‐ or middle‐income countries. No obvious reason for the difference was found, but it does suggest a need for a more even spread of studies conducted in low‐, middle‐ and high‐income countries.

It appears from this review and others, that mobile health programmes are increasingly maturing from their 'pilotitis' status (Huang 2017), in so far as pilot studies that usually have small client participant numbers. Across the included studies, four had programmes serving between 101 to 1000 clients, 22 were implemented across multiple facilities, communities, and subdistricts, and seven of these were implemented in 20 or more sites. One study reported on a national mobile health programme. However, programmes remain relatively small in terms of health worker participants: only three studies had more than 100 worker participants, and 26 studies had between 11 and 100 workers. Mobile health programmes suffer from 'pilotitis' with respect to sustaining it over time. Only two have been running for six years or longer, three between two and three years, and two studies were implemented between one and two years. The implementation period of the remaining studies was either unclear or shorter than one year. From our full‐text screening, it is also clear that the number of qualitative studies reporting on mobile health programmes is increasing.

Our findings resonate with results from primary studies that did not meet our inclusion criteria, but which reported benefits and challenges similar to what we found, when using mobile devices to deliver or support primary healthcare services. This includes benefits, such as reducing travel time, and its related expenses, for health workers and clients alike (Hurt 2016), real‐time communication (Wang 2018), and sourcing information from the Internet (Anderson‐Lewis 2018). Challenges listed in these primary studies include similar issues found in our review, for instance poor network connectivity, costs, and protecting client information (Barron 2018; Boonstra 2018; McCloud 2016; Siedner 2012).

Our synthesis identified issues that have been less reported in peer‐reviewed literature. These include: health workers advocating for political stewardship from ministries of health; workers reporting that it is more likely to turn intervention care into routine care when the mobile platform is integrated with other routine electronic systems; an acknowledgement that there is still a need for face‐to‐face interaction with colleagues and clients, and workers who often use their personal devices without remuneration for its associated costs. Though not surprising, there were health workers who perceived some clients to be more responsive and taking ownership for their health, because their consultations included the use of mobile devices. The reasons offered for this ranged from clients being concerned about the immediacy with which workers could report when they defaulted on treatment, to clients perceiving the devices as more trustworthy than standard care.

### Limitations of the review

As we no longer had access to the Global Health Ovid database for the 2018 search, this could be considered a limitation of the review. The 2015 search yielded only 870 records from that database, and it could be expected to have yielded less in 2018, given the shorter time period. However, it remains that some eligible studies may have been missed.

Through our sampling, we excluded 10 studies because of serious methodological limitations, and it is possible that some of these may have contained data that could have added nuances to our findings and/or resulted in new findings. However, this concern is to an extent balanced out by the fact these additional studies would likely not have increased the confidence in existing findings because of their methodological limitations.

## Authors' conclusions

### Implications for practice

Below are a set of questions that are drawn from the high‐ and moderate‐confidence findings in this review, and that may help implementation agencies, ministries of health, programme managers, and other stakeholders to plan, implement, or manage mobile health programmes.

#### Health systems questions

1. Will health workers be part of the planning, implementation, and evaluation processes of mobile health programmes? Will their views be sought, and their perspectives taken at each stage of the programme?
2. To what extent is political buy‐in from health ministries required, and achieved, for the successful implementation of the mobile health programme?
3. Has a proper assessment been made on whether health workers' use of mobile devices is adding to or alleviating their workload? How will the extra workload that may occur, be accommodated for?
4. If your intervention is intended to improve efficiency and co‐ordination, is the health system in which it is set prepared for the extra demands that this may imply? For example, if a health worker calls for an ambulance or for professional backup, will such support be available; if an mHealth screening intervention results in increased clients at facilities, will the existing capacity of facilities be able to handle the increased workload? If no preparation is in place for extra demands on the health system, have you engaged with those who may be required to provide additional services, so they can make preparations?
5. Do higher‐level health workers have the time and means to respond when lower‐level workers send them requests via mobile devices, and have lower‐level workers' use of mobile devices been properly explained to all higher‐level workers with whom they interact in delivering health care?
6. Does your intervention require health workers at the same or different levels of hierarchy, to interact with each other? If so, are these health workers prepared for, and willing for the changes that may arise as a result of this interaction, such as new forms of supervision and accountability, immediacy of contact, and telephonic request for advice? What needs to be done to better prepare these relationships for the anticipated changes in expectations on all parties as a result of mHealth?

#### Technical and infrastructural questions

1. Does your setting have the necessary infrastructural and technological capacity to support the level of sophistication intended by the intervention, for example: is there sufficient electricity supply and electricity coverage, network capacity, technical support, and vendors to purchase phone credit or data for the level of intervention that you intend to implement? Have you considered how these might vary by region?
2. Are the devices being used in the intervention sufficiently sophisticated for the level of intervention being planned, and are these devices replaceable or repairable within your setting? Have you considered who will repair them, and who will cover the costs?
3. When planning mHealth programmes, has the number of staff and clients who have access to mobile devices been taken into account? Are there strategies in place when clients change their mobile phone numbers?
4. Has adequate provision been made for health workers to have sufficient phone credit and data, without having to use their own resources?
5. Is there a strategy to integrate the mobile health platform within existing electronic health information systems? Have you considered the requirements to ensure interoperability?

#### Questions about health worker training and skills

1. Has the programme management budgeted for adequate training of initial staff, refresher training and in‐service training for new staff members?
2. What is the level of digital literacy amongst those health workers who will implement the intervention, as well as managers and supervisors who will support them? What further interventions are needed to ensure adequate skill levels are present at the beginning of the intervention and maintained over the course of the intervention?
3. Has the programme management identified 'champions' amongst the workers whom they can call upon to assist those struggling with the devices?
4. When the device allows the health worker to screen and diagnose clients, are they clinically equipped to respond appropriately to the results of the screening and diagnosing? Are they able to explain the results to the patient?
5. Is there a system in place to allow staff who dislike, or who are not sufficiently digitally literate to use mobile devices, to continue with standard practice, such as a paper‐based system for recording work?

#### Questions about sociocultural acceptance and equity

1. Has enough been done to raise community‐ and client‐level awareness of the mobile health programme, and its implications for the services delivered by it?
2. What is the level of cultural acceptability of mHealth in the proposed setting? What is the existing level of trust between healthcare workers and the community? Have you considered that low levels of trust may be exacerbated by mHealth, for example fears about personal data?
3. What other interventions are needed to increase trust, enhance acceptability of mHealth, and reduce skepticism amongst recipient communities?
4. Are there specific social or geographical barriers which may interact with the intervention, such as women not being allowed access to phones? How might these be addressed in advance?
5. Have you considered how barriers to mHealth use may further increase inequity, and what other interventions are required to reduce these inequities?

### Implications for future research

1. More studies are needed from high‐income countries and low‐ and middle‐income countries outside of Africa.
2. In general, researchers should aim for better reporting of their studies. This includes providing detailed information on:
  - the contexts in which the mobile devices are used, as this is likely to shape the acceptability, feasibility, and effectiveness of using mobile devices;
  - their methods of sampling, data collection and analysis; and
  - reflections on how the researchers' views and positions may have influenced the results.
3. Suggestions regarding how to report mHealth interventions can be found in the 'mHealth evidence reporting and assessment (mERA) checklist' (Agarwal 2016).
4. Researchers should give prominence to participants' voices in their studies, and present rich data, where important for a proper understanding of the phenomenon.
5. More qualitative research should be conducted alongside effectiveness studies to explore the results of effectiveness studies. We suggest that detailed programmatic and realist evaluations (Pawson 2001), become part of effectiveness studies.
6. Though individual mobile health programmes may be implemented at scale regarding client participants, and to a lesser extent, health worker participants, more longitudinal research of these programmes are needed to assess the sustainable integration of mHealth into standard care.

## Appendices

### Appendix 1. Cochrane mobile health intervention reviews not included in the WHO guidelines

1. Atherton H, Sawmynaden P, Sheikh A, Majeed A, Car J. Email for clinical communication between patients/caregivers and healthcare professionals. *Cochrane Database of Systematic Reviews* 2012a; Issue 11. www.cochranelibrary.com/cdsr/doi/10.1002/14651858.CD007978.pub2.
2. Atherton H, Sawmynaden P, Meyer B, Car J. Email for the coordination of healthcare appointments and attendance reminders. *Cochrane Database of Systematic Reviews* 2012b; Issue 8. www.cochranelibrary.com/cdsr/doi/10.1002/14651858.CD007981.pub2.
3. Chuchu N, Dinnes J, Takwoingi Y, Matin RN, Bayliss SE, Davenport C, et al. Teledermatology for diagnosing skin cancer in adults. *Cochrane Database of Systematic Reviews* 2018; Issue 12. www.cochranelibrary.com/cdsr/doi/10.1002/14651858.CD013193/full.
4. Chuchu N, Takwoingi Y, Dinnes J, Matin RN, Bassett O, Moreau JF, et al. Smartphone applications for triaging adults with skin lesions that are suspicious for melanoma. *Cochrane Database of Systematic Reviews* 2018; Issue 12. www.cochranelibrary.com/cdsr/doi/10.1002/14651858.CD013192/full.
5. de Jongh T, Gurol‐Urganci I, Vodopivec‐Jamsek V, Car J, Atun R. Mobile phone messaging for facilitating self‐management of long‐term illnesses. *Cochrane Database of Systematic Reviews* 2012; Issue 12. www.cochranelibrary.com/cdsr/doi/10.1002/14651858.CD007459.pub2.
6. Fiander M, McGowan J, Grad R, Pluye P, Hannes K, Labrecque M, et al. Interventions to increase the use of electronic health information by healthcare practitioners to improve clinical practice and patient outcomes. *Cochrane Database of Systematic Reviews* 2015; Issue 3. www.cochranelibrary.com/es/cdsr/doi/10.1002/14651858.CD004749.pub3/full.
7. Gurol‐Urganci I, de Jongh T, Vodopivec‐Jamsek V, Car J, Atun R. Mobile phone messaging for communicating results of medical investigations*. Cochrane Database of Systematic Reviews* 2012; Issue 6. www.cochranelibrary.com/cdsr/doi/10.1002/14651858.CD007456.pub2/full.
8. Gurol‐Urganci I, de Jongh T, Vodopivec‐Jamsek V, Atun R, Car J. Mobile phone messaging reminders for attendance at healthcare appointments. *Cochrane Database of Systematic Reviews* 2013; Issue 12. www.cochranelibrary.com/cdsr/doi/10.1002/14651858.CD007458.pub3/full.
9. Horvath T, Azman H, Kennedy GE, Rutherford GW. Mobile phone text messaging for promoting adherence to antiretroviral therapy in patients with HIV infection. *Cochrane Database of Systematic Reviews* 2012; Issue 3. www.cochranelibrary.com/cdsr/doi/10.1002/14651858.CD009756/full.
10. Kaner EF, Beyer FR, Garnett C, Crane D, Brown J, Muirhead C, et al. Personalised digital interventions for reducing hazardous and harmful alcohol consumption in community‐dwelling populations. *Cochrane Database of Systematic Reviews* 2017; Issue 9. www.cochranelibrary.com/cdsr/doi/10.1002/14651858.CD011479.pub2/full.
11. Kauppi K, Valimaki M, Hatonen HM, Kuosmanen LM, Warwick‐Smith K, Adams CE. Information and communication technology based prompting for treatment compliance for people with serious mental illness. *Cochrane Database of Systematic Reviews* 2014; Issue 6. www.cochranelibrary.com/cdsr/doi/10.1002/14651858.CD009960.pub2/full.
12. Khan F, Amatya B, Kesselring J, Galea M. Telerehabilitation for persons with multiple sclerosis. *Cochrane Database of Systematic Reviews* 2015; Issue 4. www.cochranelibrary.com/cdsr/doi/10.1002/14651858.CD010508.pub2/full.
13. Kuster AT, Dalsbo TK, Luong Thanh BY, Agarwal A, Durand‐Moreau QV, Kirkehei I. Computer‐based versus in‐person interventions for preventing and reducing stress in workers. *Cochrane Database of Systematic Reviews* 2017; Issue 8. www.cochranelibrary.com/cdsr/doi/10.1002/14651858.CD011899.pub2/full.
14. Lavender T, Richens Y, Milan SJ, Smyth RMD, Dowswell T. Telephone support for women during pregnancy and the first six weeks postpartum. *Cochrane Database of Systematic Reviews* 2013; Issue 7. www.cochranelibrary.com/cdsr/doi/10.1002/14651858.CD009338.pub2/full.
15. McCabe C, McCann M, Brady AM. Computer and mobile technology interventions for self‐management in chronic obstructive pulmonary disease. *Cochrane Database of Systematic Reviews* 2017; Issue 5. www.cochranelibrary.com/cdsr/doi/10.1002/14651858.CD011425.pub2/full.
16. Marcano Belisario JS, Huckvale K, Greenfield G, Car J, Gunn LH. Smartphone and tablet self management apps for asthma. *Cochrane Database of Systematic Reviews* 2013; Issue 11. www.cochranelibrary.com/cdsr/doi/10.1002/14651858.CD010013.pub2/full.
17. Meyer B, Atherton H, Sawmynaden P, Car J. Email for communicating results of diagnostic medical investigations to patients. *Cochrane Database of Systematic Reviews* 2008; Issue 8. www.cochranelibrary.com/cdsr/doi/10.1002/14651858.CD007980.pub2/full.
18. Goyder C, Atherton H, Car M, Heneghan CJ, Car J. Email for clinical communication between healthcare professionals. *Cochrane Database of Systematic Reviews* 2015; Issue 2. www.cochranelibrary.com/cdsr/doi/10.1002/14651858.CD007979.pub3/full.
19. Sawmynaden P, Atherton H, Majeed A, Car J. Email for the provision of information on disease prevention and health promotion. *Cochrane Database of Systematic Reviews* 2012;Issue 11. www.cochranelibrary.com/cdsr/doi/10.1002/14651858.CD007982.pub2/full.
20. Smith C, Gold J, Ngo TD, Sumpter C, Free C. Mobile phone‐based interventions for improving contraception use. *Cochrane Database of Systematic Reviews* 2015; Issue 6. www.cochranelibrary.com/cdsr/doi/10.1002/14651858.CD011159.pub2/full.
21. Thabrew H, Stasiak K, Hetrick SE, Wong S, Huss JH, Merry SN. eHealth interventions for anxiety and depression in children and adolescents with long‐term physical conditions. *Cochrane Database of Systematic Reviews* 2017; Issue 1 www.cochranelibrary.com/cdsr/doi/10.1002/14651858.CD012489.pub2/full.
22. Thomas R, Barker L, Rubin G, Dahlmann‐Noor A. Assistive technology for children and young people with low vision. *Cochrane Database of Systematic Reviews* 2015; Issue 6. www.cochranelibrary.com/cdsr/doi/10.1002/14651858.CD011350.pub2/full.
23. Van der Roest HG, Wenborn J, Pastink C, Dröes RM, Orrell M. Assistive technology for memory support in dementia. *Cochrane Database of Systematic Reviews* 2017; Issue 6. www.cochranelibrary.com/cdsr/doi/10.1002/14651858.CD009627.pub2/full.
24. Vodopivec‐Jamsek V, de Jongh T, Gurol‐Urganci I, Atun R, Car J. Mobile phone messaging for preventive health care. *Cochrane Database of Systematic Reviews* 2012; Issue 12. www.cochranelibrary.com/cdsr/doi/10.1002/14651858.CD007457.pub2/full.
25. Wei I, Pappas Y, Car J, Sheikh A, Majeed A. Computer‐assisted versus oral‐and‐written dietary history taking for diabetes mellitus. *Cochrane Database of Systematic Reviews* 2011; Issue 12. www.cochranelibrary.com/cdsr/doi/10.1002/14651858.CD008488.pub2/full.

### Appendix 2. mHealth technologies (source: *Wikipedia*)

#### Electronic mail (Email)

Email is a method of exchanging digital messages from an author to one or more recipients. Modern email operates across the Internet or other computer networks.

#### Personal digital assistant (PDA)

Also known as a handheld personal computer or personal data assistant, a PDA is a mobile device that functions as a personal information manager. Nearly all PDAs have the ability to connect to the Internet. A PDA has an electronic visual display, enabling it to include a web browser. It also has audio capabilities enabling use as a portable media player, and also allowing most to be used as mobile phones. Most PDAs can access the Internet, intranets or extranets via Wi‐Fi or Wireless Wide Area Networks. Most PDAs employ touch screen technology.

#### Portable media player

Also known as MP3 and MP4 players, it is a portable digital consumer electronics device capable of storing and playing digital media such as audio, images, and video files. The data are typically stored on a CD/DVD, flash memory, micro drive, or hard drive.

#### Text messaging

Also known as texting, is the act of composing and sending brief, electronic messages between two or more mobile phones, or fixed or portable devices over a phone network. The term originally referred to messages sent using the Short Message Service (SMS). It has grown to include messages containing image, video, and sound content Multimedia Message Service (MMS). BlackBerry Messenger (BBM) is a proprietary Instant Messenger application available for BlackBerry and Android mobile phones.

#### Web application

It is any software that runs in a web browser.

#### Web browser

Commonly referred to as a browser, it is a software application for retrieving, presenting and traversing information resources on the World Wide Web.

#### WhatsApp Messenger

It is an instant messaging app for smartphones that operates under a subscription business model. The proprietary, cross‐platform app enables users of select feature phones to use the Internet to communicate.

#### Other technologies

Handheld video game consoles, e.g. Playstation Portable, Nintendo DS; and handheld computers, e.g. tablets, Ipad and Smartbooks.

### Appendix 3. Search strategies

**MEDLINE Epub Ahead of Print, In‐Process & Other Non‐Indexed Citations, MEDLINE Daily and MEDLINE 1946 to Present, Ovid and Embase 1974 to 2018 January 11, Ovid (searched 12 January 2018)**

**Table:**

**#**	**Searches**	**Results**
1	Telemedicine/	36076
2	Smartphone/	5904
3	Cell Phones/	19935
4	"Cell Phone Use"/	9
5	Text Messaging/	5112
6	Electronic Mail/	18265
7	Computers, Handheld/	4540
8	MP3‐Player/	346
9	Mobile Applications/	7190
10	Medical Informatics Applications/	20613
11	Health Information Exchange/	10178
12	Health Smart Cards/	249
13	(mobile health* or mobile care or mhealth* or m health*).ti,ab,kf.	7124
14	(electronic health* or electronic care or ehealth* or e health*).ti,ab,kf.	33692
15	(telemedicine or tele medicine or telehealth or tele health or telecare or tele care or telemonitoring or tele monitoring).ti,ab,kf.	29955
16	(mobile device? or mobile electronic device?).ti,ab,kf.	4762
17	(mobile adj (phone* or telephone*)).ti,ab,kf.	14543
18	(wireless adj (phone* or telephone*)).ti,ab,kf.	249
19	(cell* phone* or cellphone*).ti,ab,kf.	7059
20	((mobile or cellular) adj (technology or technologies)).ti,ab,kf.	3135
21	((mobile or phone or telephone) adj (app? or application?)).ti,ab,kf.	5575
22	(portable electronic adj (app? or application?)).ti,ab,kf.	6
23	(smartphone* or smart phone*).ti,ab,kf.	14737
24	mobile communication.ti,ab,kf.	947
25	mobile telecommunicat*.ti,ab,kf.	381
26	personal digital assistant?.ti,ab,kf.	2185
27	patient monitor* device?.ti,ab,kf.	68
28	smart card*.ti,ab,kf.	724
29	text messag*.ti,ab,kf.	6390
30	(electronic mail? or email? or e mail?).ti,ab,kf.	34566
31	short messag* service?.ti,ab,kf.	1812
32	(sms adj (messag* or service*)).ti,ab,kf.	425
33	((multi media or multimedia) adj messag* service?).ti,ab,kf.	91
34	(mms adj (messag* or service*)).ti,ab,kf.	29
35	web messag*.ti,ab,kf.	51
36	(whatsapp or whats app).ti,ab,kf.	361
37	(instant messaging or instant messenger).ti,ab,kf.	463
38	((handheld or hand held) adj computer?).ti,ab,kf.	1452
39	(computer tablet? or pc tablet? or palmtop computer? or palm top computer? or pda computer? or pocket pc? or pda phone or blackberry or palm pilot? or pilot palm?).ti,ab,kf.	1949
40	((handheld or hand held) adj3 console?).ti,ab,kf.	27
41	(mp3player? or mp3 player? or mp4player? or mp4 player?).ti,ab,kf.	276
42	(ipod or ipods or i pod or i pods).ti,ab,kf.	866
43	portable media player?.ti,ab,kf.	36
44	pager?.ti,ab,kf.	901
45	global position? system?.ti,ab,kf.	108
46	((3G or 4G) adj system?).ti,ab,kf.	26
47	(bluetooth technolog* or blue tooth technolog*).ti,ab,kf.	90
48	(videoconsult* or video consult*).ti,ab,kf.	403
49	or/1‐48	198819
50	(qualitative or themes).mp.	514385
51	49 and 50	9970
52	51 use ppez	4357
53	remove duplicates from 52	3882
54	telehealth/	22014
55	exp telemedicine/	54001
56	mobile phone/ or mobile phones/	21671
57	cell phone/ or cell phones/	21816
58	"cell phone use"/	9
59	smartphone/ or smartphones/ or smart phone/	5932
60	text messaging/	5112
61	e‐mail/	18265
62	microcomputer/	29741
63	mp3 player/	346
64	mobile application/	8062
65	medical informatics/	29303
66	medical information system/	18336
67	smart card/	249
68	(mobile health* or mobile care or mhealth* or m health*).ti,ab,kw.	7930
69	(electronic health* or electronic care or ehealth* or e health*).ti,ab,kw.	35444
70	(telemedicine or tele medicine or telehealth or tele health or telecare or tele care or telemonitoring or tele monitoring).ti,ab,kw.	31930
71	(mobile device? or mobile electronic device?).ti,ab,kw.	4821
72	(mobile adj (phone* or telephone*)).ti,ab,kw.	14601
73	(wireless adj (phone* or telephone*)).ti,ab,kw.	253
74	(cell* phone* or cellphone*).ti,ab,kw.	7375
75	((mobile or cellular) adj (technology or technologies)).ti,ab,kw.	3151
76	((mobile or phone or telephone) adj (app? or application?)).ti,ab,kw.	5489
77	(portable electronic adj (app? or application?)).ti,ab,kw.	4
78	(smartphone* or smart phone*).ti,ab,kw.	14921
79	mobile communication.ti,ab,kw.	966
80	mobile telecommunicat*.ti,ab,kw.	385
81	personal digital assistant?.ti,ab,kw.	2206
82	patient monitor* device?.ti,ab,kw.	69
83	smart card*.ti,ab,kw.	753
84	text messag*.ti,ab,kw.	6459
85	(electronic mail? or email? or e mail?).ti,ab,kw.	34652
86	short messag* service?.ti,ab,kw.	1845
87	(sms adj (messag* or service*)).ti,ab,kw.	428
88	((multi media or multimedia) adj messag* service?).ti,ab,kw.	94
89	(mms adj (messag* or service*)).ti,ab,kw.	29
90	web messag*.ti,ab,kw.	51
91	(whatsapp or whats app).ti,ab,kw.	363
92	(instant messaging or instant messenger).ti,ab,kw.	466
93	((handheld or hand held) adj computer?).ti,ab,kw.	1503
94	(computer tablet? or pc tablet? or palmtop computer? or palm top computer? or pda computer? or pocket pc? or pda phone or blackberry or palm pilot? or pilot palm?).ti,ab,kw.	1977
95	((handheld or hand held) adj3 console?).ti,ab,kw.	27
96	(mp3player? or mp3 player? or mp4player? or mp4 player?).ti,ab,kw.	286
97	(ipod or ipods or i pod or i pods).ti,ab,kw.	879
98	portable media player?.ti,ab,kw.	36
99	pager?.ti,ab,kw.	902
100	global position? system?.ti,ab,kw.	109
101	((3G or 4G) adj system?).ti,ab,kw.	26
102	(bluetooth technolog* or blue tooth technolog*).ti,ab,kw.	90
103	(videoconsult* or video consult*).ti,ab,kw.	412
104	or/54‐103	253880
105	qualitative.mp.	455465
106	104 and 105	9450
107	limit 106 to (conference abstract or conference paper or conference proceeding or "conference review") [Limit not valid in Ovid MEDLINE(R),Ovid MEDLINE(R) Daily Update,Ovid MEDLINE(R) In‐Process,Ovid MEDLINE(R) Publisher; records were retained]	5375
108	107 use oemezd	1388
109	remove duplicates from 108	1382
110	53 or 109	5264
111	remove duplicates from 110	5232
112	111 use oemezd [Embase]	1369
113	111 use ppez [MEDLINE]	3863

**CINAHL 1981 to present, EbscoHost (searched 11 January 2018)**

**Table:**

**#**	**Searches**	**Results**
S47	S44 OR S45 Limiters ‐ Exclude MEDLINE records	870
S46	S44 OR S45	1,788
S45	S1 OR S2 OR S3 OR S4 OR S5 OR S6 OR S7 OR S8 OR S9 OR S10 OR S11 OR S12 OR S13 OR S14 OR S15 OR S16 OR S17 OR S18 OR S19 OR S20 OR S21 OR S22 OR S23 OR S24 OR S25 OR S26 OR S27 OR S28 OR S29 OR S30 OR S31 OR S32 OR S33 OR S34 OR S35 OR S36 OR S37 OR S38 OR S39 OR S40 OR S41 OR S42 OR S43 Limiters ‐ Clinical Queries: Qualitative ‐ High Specificity	768
S44	(S1 OR S2 OR S3 OR S4 OR S5 OR S6 OR S7 OR S8 OR S9 OR S10 OR S11 OR S12 OR S13 OR S14 OR S15 OR S16 OR S17 OR S18 OR S19 OR S20 OR S21 OR S22 OR S23 OR S24 OR S25 OR S26 OR S27 OR S28 OR S29 OR S30 OR S31 OR S32 OR S33 OR S34 OR S35 OR S36 OR S37 OR S38 OR S39 OR S40 OR S41 OR S42 OR S43) and qualitative	1,468
S43	TI ( videoconsult* or video W0 consult* ) OR AB ( videoconsult* or video W0 consult* )	28
S42	TI ( bluetooth W0 technolog* or "blue tooth" W0 technolog* ) OR AB ( bluetooth W0 technolog* or "blue tooth" W0 technolog* )	10
S41	TI ( (3G or 4G) W0 system* ) OR AB ( (3G or 4G) W0 system* )	133
S40	TI global W0 position* W0 system* OR AB global W0 position* W0 system*	382
S39	TI ( pager or pagers ) OR AB ( pager or pagers )	183
S38	TI ( "portable media player" or "portable media players" ) OR AB ( "portable media player" or "portable media players" )	7
S37	TI ( ipod or ipods or "i pod" or "i pods" ) OR AB ( ipod or ipods or "i pod" or "i pods" )	151
S36	TI ( mp3player* or mp3 W0 player* or mp4player* or mp4 W0 player* ) OR AB ( mp3player* or mp3 W0 player* or mp4player* or mp4 W0 player* )	63
S35	( (handheld or "hand held") N3 console* ) OR ( (handheld or "hand held") N3 console* )	3
S34	TI ( "computer tablet" or "computer tablets" or "pc tablet" or "pc tablets" or "palmtop computer" or "palmtop computers" or "palm top computer" or "palm top computers" or "pda computer" or "pda computers" or "pocket pc" or "pocket pcs" or "pda phone" or "pda phones" or blackberry or "palm pilot" or "palm pilots" or "pilot palm" or "pilot palms" ) OR AB ( "computer tablet" or "computer tablets" or "pc tablet" or "pc tablets" or "palmtop computer" or "palmtop computers" or "palm top computer" or "palm top computers" or "pda computer" or "pda computers" or "pocket pc" or "pocket pcs" or "pda phone" or "pda phones" or blackberry or "palm pilot" or "palm pilots" or "pilot palm" or "pilot palms" )	172
S33	( (handheld or "hand held") W0 computer* ) OR ( (handheld or "hand held") W0 computer* )	278
S32	TI ( "instant messaging" or "instant messenger" ) OR AB ( "instant messaging" or "instant messenger" )	98
S31	TI ( whatsapp or "whats app" ) OR AB ( whatsapp or "whats app" )	33
S30	TI "web messag*" OR AB "web messag*"	10
S29	TI ( mms W0 (messag* or service*) ) OR AB ( mms W0 (messag* or service*) )	0
S28	TI ( ("multi media" or multimedia) W0 (messag* W0 service*) ) OR AB ( ("multi media" or multimedia) W0 (messag* W0 service*) )	11
S27	TI ( sms W0 (messag* or service*) ) OR AB ( sms W0 (messag* or service*) )	34
S26	TI short W0 messag* W0 service* OR AB short W0 messag* W0 service*	195
S25	TI ( electronic W0 mail* or email* or e W0 mail* ) OR AB ( electronic W0 mail* or email* or e W0 mail* )	5,126
S24	TI text W0 messag* OR AB text W0 messag*	887
S23	TI (patient W0 monitor* W0 device* or smart W0 card*) OR AB (patient W0 monitor* W0 device* or smart W0 card*)	68
S22	TI ( "personal digital assistant" or "personal digital assistants" ) OR AB ( "personal digital assistant" or "personal digital assistants" )	442
S21	TI mobile W0 telecommunicat* OR AB mobile W0 telecommunicat*	18
S20	TI (mobile W0 communication* or wireless W0 communication*) OR AB (mobile W0 communication* or wireless W0 communication*)	152
S19	TI ( smartphone* or smart W0 phone* ) OR AB ( smartphone* or smart W0 phone* )	1,620
S18	TI ( "portable electronic" W0 (app or apps or application*) ) OR AB ( "portable electronic" W0 (app or apps or application*) )	615
S17	TI ( (mobile or phone or telephone) W0 (app or apps or application*) ) OR AB ( (mobile or phone or telephone) W0 (app or apps or application*) )	758
S16	TI ( (mobile or cellular) W0 (technology or technologies) ) OR AB ( (mobile or cellular) W0 (technology or technologies) )	424
S15	TI (cell* W0 phone* or cellphone*) OR AB (cell* W0 phone* or cellphone*)	938
S14	TI ( mobile W0 phone* or mobile W0 telephone* or wireless W0 phone* or wireless W0 telephone* ) OR AB ( mobile W0 phone* or mobile W0 telephone* or wireless W0 phone* or wireless W0 telephone* )	1,147
S13	TI (mobile W1 device* or wireless W1 device*) OR AB (mobile W1 device* or wireless W1 device*)	741
S12	TI ( telemedicine or "tele medicine" or telehealth or "tele health" or telecare or "tele care" or telemonitoring or "tele monitoring" ) OR AB ( telemedicine or "tele medicine" or telehealth or "tele health" or telecare or "tele care" or telemonitoring or "tele monitoring" )	3,925
S11	TI ( "electronic health*" or "electronic care" or ehealth* or "e health*" ) OR AB ( "electronic health*" or "electronic care" or ehealth* or "e health*" )	5,185
S10	TI ( "mobile health*" or "mobile care" or mhealth* or "m health*" ) OR AB ( "mobile health*" or "mobile care" or mhealth* or "m health*" )	728
S9	(MH "Wireless Communications")	6,949
S8	(MH "Health Informatics+") or (MH "Smart Cards")	6,886
S7	(MH "Mobile Applications")	1,847
S6	(MH "Computers, Hand‐Held")	2,698
S5	(MH "Electronic Mail")	21
S4	(MH "Text Messaging")	787
S3	(MH "Smartphone")	786
S2	(MH "Cellular Phone")	495
S1	(MH "Telehealth+")	10,296

**Science Citation Index and Social Sciences Citation Index 1987‐present, and Emerging Sources Citation Index 2015‐present, Web of Science (searched 12 January 2018)**

**Table:**

**#**	**Searches**
#1	TS=("mobile health*" or "mobile care" or mhealth* or "m health*")
#2	TS=(qualitative)
#3	#1 and #2

**Global Health 1973 to 2015 Week 48, Ovid (searched 08 December 2015)**

**Table:**

**#**	**Searches**	**Results**
1	(mobile health or mobile care or mhealth* or m health*).mp.	287
2	(electronic health or electronic care or ehealth or e health).mp.	971
3	(telemedicine or tele medicine or telehealth or tele health or telecare or tele care or telemonitoring or tele monitoring).mp.	896
4	(mobile device? or mobile electronic device? or wireless device*).mp.	107
5	(mobile adj (phone* or telephone*)).mp.	1501
6	cell* phone*.mp.	549
7	((mobile or cellular) adj (technology or technologies)).mp.	127
8	((mobile or phone or telephone) adj (app? or application?)).mp.	110
9	(portable electronic adj (app? or application?)).mp.	0
10	(smartphone* or smart phone?).mp.	256
11	(mobile communication? or wireless communication?).mp.	115
12	mobile telecommunicat*.mp.	35
13	personal digital assistant?.mp.	129
14	patient monitor* device?.mp.	1
15	text messag*.mp.	518
16	(electronic mail? or email? or e mail?).mp.	1213
17	short messag* service?.mp.	145
18	(sms adj (messag* or service*)).mp.	34
19	((multi media or multimedia) adj messag* service?).mp.	2
20	(mms adj (messag* or service*)).mp.	0
21	web messaging.mp.	0
22	(whatsapp or whats app).mp.	2
23	(instant messaging or instant messenger).mp.	18
24	((handheld or hand held) adj computer?).mp.	82
25	(computer tablet? or pc tablet? or palmtop computer? or palm top computer? or pda computer? or pocket pc? or pda phone or blackberry or palm pilot? or pilot palm?).mp.	311
26	((handheld or hand held) adj3 console?).mp.	0
27	(mp3player? or mp3 player? or mp4player? or mp4 player?).mp.	12
28	(ipod or ipods or i pod or i pods).mp.	23
29	portable media player?.mp.	1
30	pager?.mp.	19
31	global position? system?.mp.	12
32	((3G or 4G) adj system?).mp.	0
33	(bluetooth technolog* or blue tooth technolog*).mp.	1
34	(videoconsult* or video consult*).mp.	9
35	or/1‐34	5634
36	qualitative.mp.	28243
37	35 and 36	271

#### Other resources

##### For related systematic reviews:

**Cochrane library:**http://www.cochranelibrary.com (searched 21 February 2018)

· mHealth AND healthcare worker

· mHealth AND health provider

· mHealth AND perceptions

· mobile health

**PDQ:**http://www.pdq‐evidence.org (searched 21 February 2018)

· mHealth AND healthcare worker AND perceptions

· mHealth AND healthcare worker

· mHealth AND healthcare worker

· mHealth

· mobile devices

· mHealth with 2015‐2018 as time range

· mHealth with last 5 years filter and primary studies

**McMaster Health Evidence:**https://www.healthevidence.org

*2018 search*

· mHealth AND primary health care provider with 2015‐2018 as time range

· mobile health AND primary health care provider with 2015‐2018 as time range

· mobile phone AND health care provider with 2015‐2018 as time range

· mobile phone based AND health care provider with 2015‐2018 as time range

##### Grey literature:

**Eldis:**www.eldis.org (searched 16 February 2018)

· Topic: Digital development with search term: mobile health

· Topic: Digital development with search term: mobile phone

· Topic: Digital development with search term: health workers

· Topic: Health with search term: mHealth

· Topic: Health system with search term: mHealth

**Google Scholar:**https://scholar.google.co.za (searched 21 February 2018)

· "mHealth" OR "mobile health" OR "mobile phones" AND "health workers" OR "healthcare worker"

· "mHealth" OR "mobile health" OR "mobile phones" AND "health workers" OR "health worker" AND "perceptions" OR "experiences"

· For the 2020 search: some strategy with time limit between 2018‐2020

**mHealth Database:**www.africanstrategies4health.org/mhealth‐database.html (searched 05 March 2018)

*2018 search*

· Screen the 2016 compendium

**mHealth Evidence:**www.mhealthevidence.org (searched 21 February 2018)

*2018 search*

· qualitative study

**mHealth Knowledge:**http://mhealthknowledge.org (searched 05 March 2018)

*2018 search*

· Searched mHealth Alliance link

· Searched Communities of practice link

· Searched Capacity and learning link

· Searched Project repository > Examples from LMIC link

· Searched Project repository > Group inventory link

**mPowering:**https://partnerships.usaid.gov/partnership/mpowering‐frontline‐health‐workers (searched 05 March 2018 )

*2018 search*

· Searched 'Resources'

· Searched Resources > External links

· Searched 'mHealth'

**OpenGrey:**www.opengrey.eu (searched 16 February 2018)

· "mobile health"

· "mobile health" > "health" > "2011"

· "mHealth"

· "mobile health" AND "health workers"

**The Grey Literature Report:**www.greylit.org (searched 21 February 2018)

· mHealth

· mobile health

· mobile health with adding 'health workers' in refining criteria

### Appendix 4. GRADE‐CERQual evidence profiles

**Table:**

**Finding 1** Through being connected to other health workers and across various healthcare services, health workers appreciated that mobile devices allowed them to better co‐ordinate the delivery of care.	Barnabee 2014; Chang 2011; Hampshire 2016; Henry 2016; Huq 2014; Khan 2015; Lodhia 2016; Madon 2014; Messinger 2017; Murray 2011; Mwendwa 2016; Quinn 2013; Ramirez 2017; Rothstein 2016; Schoen 2017; Toda 2017; van der Wal 2016; Watkins 2018	Minor concerns regarding methodological limitations because the majority of studies had no to minor methodological limitations	No or very minor concerns regarding coherence	Minor concerns regarding adequacy because of thin data found in the studies	Moderate concerns regarding relevance because of the limited range of contexts in which the studies were conducted	Moderate confidence	Due to no/very minor concerns regarding coherence, minor concerns regarding adequacy, and methodological limitations, and moderate concerns regarding relevance
**Finding 2** Lower‐level health workers valued being able to reach higher‐level health workers via mobile devices, and perceived the advice and support they received as improving their care and as satisfying to clients. When higher‐level professionals responded in anger, it made lower‐level health workers reluctant to call them.	Ayiasi 2015; Chang 2011; Cherrington 2015; Hampshire 2016; Huq 2014; Khan 2015; Lodhia 2016; Madon 2014; Messinger 2017; Mwendwa 2016; Quinn 2013; Toda 2017; van der Wal 2016; Watkins 2018	Moderate concerns regarding the methodological limitations because the majority of studies had insufficient information on the data collection and analysis methods, and author reflexivity	No/very minor concerns regarding coherence	No/very minor concerns regarding adequacy	No/very minor concerns regarding relevance	Moderate confidence	Due to no/very minor concerns regarding coherence, relevance, and adequacy, but moderate concerns regarding methodological limitations
**Finding 3** When higher‐level health workers failed to respond and support lower‐level workers through mobile devices, lower‐level staff had negative perceptions of these devices. One study emphasised the importance of having health professionals' buy‐in with mobile health to ensure that mobile devices were optimally used to support lay health workers.	Cherrington 2015; Huq 2014; Mwendwa 2016; Quinn 2013; Toda 2017; van der Wal 2016	Minor concerns regarding the methodological limitations because a few studies had insufficient information on the data collection and analysis methods	No/very minor concerns regarding coherence	Moderate concerns regarding adequacy because it is supported by only a few studies with thin data	No/very minor concerns regarding relevance	Moderate confidence	Due to no/very minor concerns regarding coherence and relevance, minor concerns regarding methodological limitations, but moderate concerns regarding adequacy
**Finding 4** The use of mobile devices allowed some health workers to feel connected to their peers within their own organisations. However, others preferred face‐to‐face communication with their peers.	Barnabee 2014; Hampshire 2016; Henry 2016; Jennings 2013; Madon 2014; Valaitis 2005; van der Wal 2016; Watkins 2018	Minor concerns regarding methodological limitations due to inconsistency regarding methodological reporting in a few studies	No or very minor concerns regarding coherence	Moderate concerns regarding adequacy because the richness of the data are inconsistent across the studies	Minor concerns regarding relevance because data from four studies are pilot studies with its associated focused support to participants which is not true to real life	Moderate confidence	Due to no/very minor concerns regarding coherence, minor concerns regarding methodological limitations and relevance, and moderate concerns regarding adequacy
**Finding 5** Some health workers relayed that mobile devices improved their reporting to supervisors and encouraged them to report more truthfully. Others compared mobile device‐facilitated supervision to "big brother watching". Some supervisors thought that mobile devices allowed them to better identify staff who needed support.	Barnabee 2014; Chang 2011; Jennings 2013; Madon 2014; Medhanyie 2015; Mwendwa 2016; Schoen 2017; Toda 2017; Valaitis 2005; van der Wal 2016	Moderate concerns regarding the methodological limitations because of inconsistent support of the included data for this finding	No/very minor concerns regarding coherence	Minor concerns regarding adequacy because some of the data are supported by one study only	Minor concerns regarding relevance because the perceptions are mostly from lay health workers	Moderate confidence	Due to no/very minor concerns regarding coherence, minor concerns regarding relevance and adequacy, and moderate concerns regarding methodological limitations
**Finding 6** Health workers had positive experiences with using instant messaging through WhatsApp. This application was seen as cheap and suitable for a range of activities, such as communicating with peers and posting photos as evidence of work done.	Hampshire 2016; Henry 2016; Schoen 2017	Serious concerns regarding the methodological limitations because the study contributing most to the finding had a poor description of the context, sampling of participants, data collection and analysis.	No/very minor concerns regarding coherence	Serious concerns regarding adequacy because the finding is supported by only three studies	Moderate concerns regarding relevance because the finding is based on only three studies, with two of them being from Sub‐Saharan Africa	Very low confidence	Due to serious concerns regarding methodological limitations and adequacy, moderate concerns regarding relevance, and no/very minor concerns regarding coherence
**Finding 7** Even when health workers received messages that were automated, rather than sent directly from a manager or supervisor, this was still experienced and responded to, as a kind of supervision. Some lower‐level health workers experienced it as supportive to their work, while others felt guilty for not providing correct care as per these messages.	Cherrington 2015; Ilozumba 2018; Jones 2012; Mwendwa 2016	Minor concerns regarding methodological limitations because though all research components are presented it is not described in sufficient detail	No/very minor concerns regarding coherence	Moderate concerns regarding adequacy because it is thin data	Moderate concerns regarding relevance because most of the data relate to lay health workers only, mostly from one study	Low confidence	Due to moderate concerns regarding relevance and adequacy, minor concerns regarding methodological limitations, and no/very minor concerns regarding coherence
**Finding 8** The task optimisation enabled through mHealth interventions was widely valued by health workers.	Barnabee 2014; Chang 2011; Ilozumba 2018; Khan 2015; Kolltveit 2017; Lodhia 2016; Praveen 2014	Moderate concerns regarding the methodological limitations because in two supporting studies it was not clear how participants were selected, poor data collection description, and limited researcher reflexivity. In addition, we have only positive perceptions, which raises a concern about possible bias	No/very minor concerns regarding coherence	No/very minor concerns regarding adequacy	No/very minor concerns regarding relevance	Moderate confidence	Due to no/very minor concerns regarding coherence, relevance, and adequacy, but moderate concerns regarding methodological limitations
**Finding 9** At times, health workers used their mobile devices to access the Internet for health information, and found it useful when they were with clients who needed the information. This interaction also included health workers providing clients with additional information beyond the healthcare intervention. But, if the only way that health workers could access online information, required them to use their own money to purchase data, then this could be prohibitive to them accessing such information.	Bacchus 2016; Hampshire 2016; Schoen 2017; Watkins 2018	Minor concerns regarding methodological limitations as the majority of studies had no to minor methodological limitations	No/very minor concerns regarding coherence	Serious concerns regarding adequacy because of very thin data	Minor concerns regarding relevance due to a limited number of countries in which the studies were conducted	Low confidence	Due to no/very minor concerns regarding coherence, minor concerns regarding methodological limitations and relevance, and serious concerns regarding adequacy
**Finding 10** mHealth held the promise of increasing service efficiency for many health workers, but the experience of whether this promise was borne out in practice, varied in the accounts of health workers. It was experienced as efficient if it improved feedback, speed and workflow, but inefficient when the technology was slow and time consuming. Some were concerned that if mHealth was too efficient, making work faster, that this may justify staff cutbacks.	Ayiasi 2015; Barnabee 2014; Chang 2011; Cherrington 2015; Coetzee 2017; Garg 2016; Ginsburg 2016; Hampshire 2016; Hao 2015; Huq 2014; Jennings 2013; Jones 2012; Kolltveit 2017; Lodhia 2016; Madon 2014; Medhanyie 2015; Messinger 2017; Mwendwa 2016; Praveen 2014; Ramirez 2017; Rothstein 2016; Schoen 2017Schoen 2017; Toda 2017; Valaitis 2005; van der Wal 2016; Watkins 2018	Minor concerns regarding methodological limitations because 10 of the studies were pilot studies, which could bias the perceptions given the intensified support that is standard in pilot studies	Minor concerns regarding coherence given there were only two studies reporting negative perceptions	No/very minor concerns regarding adequacy	No/very minor concerns regarding relevance	High confidence	Due to no/very minor concerns relevance and adequacy, and minor concerns regarding methodological limitations and coherence
**Finding 11** Health workers frequently reported mobile devices as overcoming the difficulties of rural and geographically challenging contexts when it made it possible for them to provide health care without having to travel. Some reported that reducing travel time allowed them more time with their clients.	Chang 2011; Hampshire 2016; Hirsch‐Moverman 2017; Lodhia 2016; Messinger 2017; Mwendwa 2016; Quinn 2013; Rothstein 2016; Toda 2017; Valaitis 2005	Minor concerns regarding methodological limitations because more than half of the studies had poorly described data collection and analysis methods	No/very minor concerns regarding coherence	No/very minor concerns regarding adequacy	Minor concerns regarding relevance as the finding is primarily applicable to only rural and geographically challenging contexts	High confidence	Due to no/very minor concerns regarding coherence and adequacy, and minor concerns regarding methodological limitations and relevance
**Finding 12** Health workers appreciated the portability and work schedule flexibility of mobile devices.	Hampshire 2016; Murray 2011; Nguyen 2015; Orchard 2014; Ramirez 2017; Schoen 2017; Toda 2017; Valaitis 2005; van der Wal 2016	Moderate concerns regarding the methodological limitations because the majority of studies had insufficient, poorly described methods and data collection; the data in one study was hand recorded, and no ethical approval was described in another	No or very minor concerns regarding adequacy	No or very minor concerns regarding adequacy	No or very minor concerns regarding relevance	Moderate confidence	Due to no/very minor concerns regarding coherence, relevance, adequacy, but moderate concerns regarding methodological limitations
**Finding 13** Through mHealth, health workers were able to use treatment and screening algorithms that were loaded onto mobile devices. Their perceptions of using these electronic algorithms ranged from finding it easy and useful, to threatening their clinical competency, and an information overload. There were also some concerns that erroneous data entry may lead to wrong treatment guidance.	Ginsburg 2016; Ilozumba 2018; Lodhia 2016; Mitchell 2012; Mwendwa 2016; Nguyen 2015; Orchard 2014; Ramirez 2017; Rothstein 2016; Shao 2015; Surka 2014; Tewari 2017; van der Wal 2016	Minor concerns regarding methodological limitations given that the majority of studies had no or minor limitations	No/very minor concerns regarding coherence	No/very minor concerns regarding adequacy	No/very minor concerns regarding relevance	High confidence	Due to no/very minor concerns regarding coherence, relevance, and adequacy, and minor concerns regarding methodological limitations
**Finding 14** Using mobile devices to record routine client or surveillance data, was mostly perceived by health workers and their managers as helpful for decision making, and increasing community and health worker appreciation of these data.	Khan 2015; Lodhia 2016; Madon 2014; Murray 2011; Nguyen 2015; Ramirez 2017; Rothstein 2016; Schoen 2017; Toda 2017	Moderate concerns regarding methodological limitations because half of the studies had insufficient information on the context, data collection and analysis methods, and potential bias because of how the data were collected	No or very minor concerns regarding coherence	No or very minor concerns regarding adequacy	No or very minor concerns regarding relevance	Moderate confidence	Due to no/very minor concerns regarding coherence, relevance, and adequacy, but moderate concerns regarding methodological limitations
**Finding 15** In most cases health workers perceived mobile health as more advantageous than paper. However, some continued to prefer paper.	Bacchus 2016; Coetzee 2017; Ginsburg 2016; Madon 2014; Mitchell 2012; Mwendwa 2016; Nguyen 2015; Rothstein 2016; Schoen 2017; Surka 2014; Toda 2017; Valaitis 2005; van der Wal 2016; Vedanthan 2015; Watkins 2018	Minor concerns regarding methodological limitations given that the majority of studies had no or minor limitations	No/very minor concerns regarding coherence	No/very minor concerns regarding adequacy	No/very minor concerns regarding relevance	High confidence	Due to no/very minor concerns regarding coherence, relevance, and adequacy, and minor concerns regarding methodological limitations
**Finding 16** mHealth interventions sometimes required health workers to perform tasks that were peripheral to regular service delivery, such as registering clients onto the system. These more menial tasks were sometimes regarded as undermining to professional staff.	Hirsch‐Moverman 2017; Medhanyie 2015; Murray 2015; Wolff‐Piggott 2018	Serious concerns regarding the methodological limitations because two of the included studies had poor descriptions of the context, data collection and analysis methods	Moderate concerns regarding coherence because part of the finding is not coherent across the supporting studies	Serious concerns regarding adequacy because of a limited number of studies with very thin data	Moderate concerns regarding relevance because of the limited number of settings in which the studies were conducted	Very low confidence	Due to serious concerns regarding methodological limitations and adequacy, and moderate concerns regarding coherence and relevance
**Finding 17** Some health workers experienced the use of mHealth as generating an extra workload when, for instance, it resulted in reaching more clients needing care, or having to maintain both a mobile health and paper system. Some workers disliked this, particularly when their superiors did not perceive their mobile health work as part of their job description. Others did not object to the additional work, yet others wanted to be remunerated.	Chang 2011; Hao 2015; Kolltveit 2017; Lodhia 2016; Murray 2015; Mwendwa 2016; Praveen 2014; Rothstein 2016; Shao 2015; Wolff‐Piggott 2018	Minor considerations regarding methodological limitations as the majority of studies had no to minor methodological limitations	No/very minor concerns regarding coherence	No/very minor concerns regarding adequacy	No/very minor concerns regarding relevance	High confidence	Due to no/very minor concerns regarding coherence, relevance and adequacy, and minor concerns regarding methodological limitations
**Finding 18** Through mobile devices, health workers and clients could communicate directly with each other, which health workers reported as improving care and their relationship with clients. When clients initiated the contact, health workers felt that clients took ownership of their health. Health workers felt that some clients still warrant face‐to‐face contact.	Barnabee 2014; Chang 2011; Cherrington 2015; Garg 2016; Hirsch‐Moverman 2017; Huq 2014; Jennings 2013; Lodhia 2016; Messinger 2017; Schoen 2017; van der Wal 2016; Watkins 2018	Minor concerns regarding methodological limitations as only one of the studies had serious methodological limitations because of poorly described methods and study context	No/very minor concerns regarding coherence	Moderate concerns regarding adequacy because evidence on clients appreciation and perceived impact on health worker‐client relationship was limited	No or very minor concerns regarding relevance	Moderate confidence	Due to no/very minor concerns regarding coherence and relevance, minor concerns regarding methodological limitations, and moderate concerns regarding adequacy
**Finding 19** Health workers were aware of the importance of protecting confidential client information when using mobile devices, and the confidentiality risks in cases of stolen phones and using their SIM cards in colleagues' phones. Health workers were alert to clients' concerns when they shared personal information concerning stigmatised issues, such as HIV/AIDS and interpersonal violence, and suggested ways to keep the information confidential. They emphasised building a trusting relationship with clients prior to using the devices.	Bachus 2016; Coetzee 2017; Garg 2016; Hirsch‐Moverman 2017; Lodhia 2016; Murray 2015; Mwendwa 2016; Rothstein 2016; Valaitis 2005; Wolff‐Piggott 2018	No/very minor concerns regarding methodological limitations as most of the studies had no to minor methodological limitations	No/very minor concerns regarding coherence	No/very minor concerns regarding adequacy	No/very minor concerns regarding relevance	High confidence	Due to no/minor concerns regarding methodological limitations, coherence, relevance, and adequacy
**Finding 20** Health workers were concerned that concentrating too much on the mobile technology during client consultations could be to the detriment of their service and interaction with clients.	Bacchus 2016; Schoen 2017; Vedanthan 2015	Minor concerns regarding methodological limitations because two of the three studies had no to minor methodological limitations	No/very minor concerns regarding coherence	Serious concerns regarding adequacy because the finding is based on only three studies	Moderate concerns regarding relevance: though there is a range from contexts where the studies were conducted, the finding is based on only three studies, representing limited settings	Low confidence	Due to serious concerns about adequacy, moderate concerns regarding relevance, minor concerns regarding methodological limitations, and no/very minor concerns regarding coherence
**Finding 21** Health workers had differing reactions to being contactable via mobile devices during and outside of working hours: some felt it was useful, some were ambivalent about it, and others objected to it. Workers suggested setting boundaries to protect themselves from this.	Chang 2011; Cherrington 2015; Hampshire 2016; Huq 2014; Jennings 2013; Schoen 2017; Valaitis 2005	No or very minor concerns regarding methodological limitations	No/very minor concerns regarding coherence	Moderate concerns regarding adequacy because of a limited number of studies and thin data	Minor concerns regarding relevance because the supporting data are limited in the range of specific health issues	Moderate confidence	Due to no/very minor concerns regarding methodological limitations and coherence, minor concerns regarding relevance, and moderate concerns regarding adequacy
**Finding 22** Health workers experienced the use of mobile technology to provide health care, as being met with both trust and skepticism from clients and the communities they served. They described how trust or skepticism in the device was translated into trust or skepticism of their service when using the device. Some found that using mobile devices raised their social status with clients, and even their families. Others were concerned that using expensive equipment would emphasise inequity between themselves and clients.	Ayiasi 2015; Barnabee 2014; Cherrington 2015; Coetzee 2017; Ginsburg 2016; Ilozumba 2018; Jones 2012; Khan 2015; Lodhia 2016; Madon 2014; Mitchell 2012; Mwendwa 2016; Valaitis 2005; van der Wal 2016	Minor concerns regarding methodological limitations as only a few studies had insufficient information on author reflexivity, participant selection, study limitations, and ethical considerations	No/very minor concerns regarding coherence	No/very minor concerns regarding adequacy	No/very minor concerns regarding relevance	High confidence	Due to no/very minor concerns regarding coherence, relevance, and adequacy, and minor concerns regarding methodological limitations
**Finding 23** Health workers experienced clients as having an opinion not only about their use of mobile devices, but as having an opinion on the devices themselves, which influenced how they responded to care delivered with the support of these devices. Health workers ascribed clients' enthusiasm for mobile devices as due to these clients' perception of the devices as prestigious, offering trustworthy information, and providing confidentiality. They perceived clients as more receptive when these clients were familiar with the devices used. There were concerns that clients who felt that the use of these devices during care was too time consuming, and would respond negatively to its use.	Bacchus 2016; Garg 2016; Ginsburg 2016; Ilozumba 2018; Jones 2012; Khan 2015; Messinger 2017; Mitchell 2012; Schoen 2017; Shao 2015; Valaitis 2005; van der Wal 2016; Vedanthan 2015; Westergaard 2017	Moderate concerns regarding the methodological limitations, as the majority of studies had no or very minor methodological concerns, however substantial supporting data came from a study with serious methodological concerns	No/very minor concerns regarding coherence	No/very minor concerns regarding adequacy	No/very minor concerns regarding relevance	Moderate confidence	Due to no/very minor concerns regarding coherence, relevance and adequacy, but moderate concerns regarding methodological limitations
**Finding 24** Some interventions required clients to have phones as well as health workers. Health workers described this as challenging for multiple reasons, including clients not having phones, changing their phone numbers regularly, not knowing how to use a phone, being a target of crime because of possession of the phone, and women being prohibited from accessing phones. Health workers suggested competitive pricing to increase clients' access to phones, and to issue clients with phones.	Chang 2011; Hirsch‐Moverman 2017; Huq 2014; Murray 2015; Tewari 2017; van der Wal 2016; Wolff‐Piggott 2018	Minor concerns regarding methodological limitations, as only one study had serious methodological limitations with inadequate descriptions of context, data collection and analysis methods	No/very minor concerns regarding coherence	Minor concerns regarding adequacy because of the limited number of studies and thin data	Moderate concerns regarding relevance because of the limited range of settings and health worker categories	Moderate confidence	Due to no/very minor concerns regarding coherence, minor concerns regarding methodological limitations and adequacy, and moderate concerns regarding relevance
**Finding 25** Health workers were ambivalent about interventions that required clients to use the health workers' mobile devices during consultations. Their optimism was tempered by concern that there was a loss of meaningful engagement with clients.	Bacchus 2016; Coetzee 2017	No/very minor concerns regarding methodological limitations	No/very minor concerns regarding coherence	Serious concerns regarding adequacy because the finding is based on only two studies	Moderate concerns regarding relevance as none of the two studies were conducted in low‐ and lower‐middle‐income countries, and only reported lay health workers' perceptions	Low confidence	Due to serious concerns regarding adequacy, moderate concerns regarding relevance, and no/very minor concerns regarding methodological limitations and coherence
**Finding 26** Health workers reported that their access to mobile devices was beneficial to clients and communities who were too poor to own mobile phones.	Chang 2011; van der Wal 2016	Moderate concerns regarding the methodological limitations because of poorly described data collection and analysis methods in the one study contributing most of the data	No or very minor concerns regarding coherence	Serious concerns regarding adequacy because the finding is based on very thin data	Serious concerns regarding relevance because the finding is based on only two studies, which also limits the study contexts	Very low confidence	Due to serious concerns regarding relevance and adequacy, moderate concerns regarding methodological limitations, and no/very minor concerns regarding coherence
**Finding 27** Health workers felt that health promotion and educational messaging directed at clients using mobile health interventions, impacted positively on clients' health behaviours, but cautioned against repetitive showing of health promotion videos. In one instance, issuing clients with mobile phones led to increased use of healthcare services.	Bacchus 2016; Barnabee 2014; Chang 2011; Coetzee 2017; Ginsburg 2016; Huq 2014; Ilozumba 2018; Jones 2012; Lodhia 2016; Madon 2014; Murray 2011; Praveen 2014; van der Wal 2016	No/very minor concerns regarding the methodological limitations because the majority of studies had no to minor methodological limitations	No/very minor concerns regarding coherence	Moderate concerns regarding adequacy as some studies had thin data with limited discussion of health worker perceptions	No/very minor concerns regarding relevance	Moderate confidence	Due to no/very minor concerns regarding methodological limitations, coherence, and relevance, but moderate concerns regarding adequacy
**Finding 28** Some health workers accepted bearing the costs of mHealth interventions themselves, but were dissatisfied when phone credit to use the phones was not paid on time. Health workers felt that clients appreciated it when health workers called them, as it saved them costs.	Hampshire 2016; Khan 2015; Messinger 2017; Quinn 2013; van der Wal 2016; Watkins 2018; Wolff‐Piggott 2018	Minor concerns regarding methodological limitations because most of the studies has no or minor concerns	No/very minor concerns regarding coherence	No/very minor concerns regarding adequacy	No/very minor concerns regarding relevance	High confidence	Due to no/very minor concerns regarding relevance and adequacy, and minor concerns regarding methodological limitations and coherence
**Finding 29** Health workers' digital literacy impacted on their experience and perceptions of the use of mobile devices in health service delivery: being digitally literate resulted in positive experiences and perceptions, whilst low digital literacy caused concerns about job security and embarrassment when making mistakes in front of clients. For some workers, prior exposure to mobile devices did not affect their perceptions and use of mobile health. Some turned their lack of digital literacy into building a relationship with clients by asking clients to show them how to use the devices. Not using the devices often enough, resulted in loss in digital literacy	Bacchus 2016; Cherrington 2015; Coetzee 2017; Ginsburg 2016; Hao 2015; Hirsch‐Moverman 2017; Ilozumba 2018; Kolltveit 2017; Madon 2014; Mitchell 2012; Murray 2011; Mwendwa 2016; Nguyen 2015; Praveen 2014; Quinn 2013; Shao 2015; Surka 2014; Valaitis 2005; van der Wal 2016; Watkins 2018	Moderate concerns regarding methodological limitations because half of the studies had moderate concerns of which three had serious methodological limitations. The limitations included poorly described data collection and analysis methods, and in one study there were concerns that the data collection method could have biased participant responses	No/very minor concerns regarding coherence	No/very minor concerns regarding adequacy	No/very minor concerns regarding relevance	Moderate confidence	Due to no/very minor concerns regarding coherence, relevance, and adequacy, but moderate concerns regarding methodological limitations
**Finding 30** Health workers expressed a need for training and familiarity with mobile devices to overcome their initial anxiety in using the devices. Peer training from technologically proficient colleagues was experienced as valuable. In several cases, health workers wanted refresher training and pointed to the importance of training replacement staff. Not having mentors who used mobile devices, impacted negatively on lower‐level workers' ability to learn how to use these devices.	Coetzee 2017; Ginsburg 2016; Ilozumba 2018; Kolltveit 2017; Lodhia 2016; Madon 2014; Murray 2011; Mwendwa 2016; Nguyen 2015; Praveen 2014; Rothstein 2016; Tewari 2017; Toda 2017; van der Wal 2016; Vedanthan 2015	Minor concerns regarding methodological limitations because the majority of studies had no or minor methodological limitations	No/very minor concerns regarding coherence	No/very minor concerns regarding adequacy	No/very minor concerns regarding relevance	High confidence	Due to no/very minor concerns regarding coherence, relevance, and adequacy, and minor concerns regarding methodological limitations
**Finding 31** All categories of health workers required technical support to solve user problems. At times, face‐to‐face support was provided, but technical support from proficient colleagues was useful too. Having technical problems solved through real‐time improvements worked well for some health workers, while others suggested a help function be added to the devices.	Cherrington 2015; Garg 2016; Hao 2015; Ilozumba 2018; Kolltveit 2017; Lodhia 2016; Madon 2014; Murray 2011; Mwendwa 2016; Rothstein 2016; Toda 2017; van der Wal 2016	Minor concerns regarding methodological limitations as most studies had no to minor methodological limitations	No/very minor concerns regarding coherence	No/very minor concerns regarding adequacy	No/very minor concerns regarding relevance	High confidence	Due to no/very minor concerns regarding coherence, relevance, and adequacy, and minor concerns regarding methodological limitations
**Finding 32** Health workers highlighted that mobile technology applications should be user‐friendly, easy to learn, and improve the quality of their care. When the applications were not easy to use, health workers became frustrated and reluctant users of mobile devices.	Ginsburg 2016; Khan 2015; Kolltveit 2017; Lodhia 2016; Mwendwa 2016; Praveen 2014; Ramirez 2017; Rothstein 2016; Schoen 2017; Toda 2017; van der Wal 2016	Minor concerns regarding methodological limitations because only a few studies had insufficient information about the data collection and analysis methods, and author reflexivity	No/very minor concerns regarding coherence	No very minor concerns regarding adequacy	No/very minor concerns regarding relevance	High confidence	Due to no/very minor concerns regarding coherence, relevance, and adequacy, and minor concerns regarding methodological limitations
**Finding 33** Health workers held mixed views on choosing between tablets and smartphones. Some felt that the type of content on the device was more important than the device itself. However, other health workers preferred tablets over smartphones, mainly because the bigger size of the screen was perceived as easier for client engagement.	Schoen 2017; Shao 2015	Minor concerns regarding the methodological limitations because of limited description of the data analysis	No/very minor concerns regarding coherence	Serious concerns regarding adequacy because the finding is based on only two studies	Serious concerns regarding relevance because the finding is based on only two contexts	Very low confidence	Due to serious concerns regarding relevance and adequacy, minor concerns regarding methodological limitations, and no/very minor concerns regarding coherence
**Finding 34** Some health workers felt that sustainable, at scale mHealth programmes required approval and stewardship from political leaders, such as ministries of health. Leadership interest in mHealth interventions was described as motivating to health workers. Health workers suggested that such leaders should be engaged early and continuously throughout the programme, and be provided with evidence of effectiveness, so as to secure their support. The lack of high‐level stewardship impacted negatively on the mHealth programme.	Ginsburg 2016; Kolltveit 2017; Lodhia 2016	No/very minor concerns regarding methodological limitations	No/very minor concerns regarding coherence	Serious concerns regarding adequacy because the finding is based on only four studies	No/very minor concerns regarding relevance	Low confidence	Due to serious concerns regarding adequacy, and no/very minor concerns regarding methodological limitations, coherence and relevance
**Finding 35** Health worker accounts pointed to the strong influence of the health systems and social context in which the intervention was embedded. Contextual and systems issues such as difference in language use between clients and health workers, gender discrimination, discomfort with professional hierarchies, poverty, resource constraints, staff attrition, and more, all of which were external to the technology and the physical device, influenced how health workers experienced mHealth and the use of mobile devices for service delivery, in their different contexts.	Chang 2011; Huq 2014; Khan 2015; Kolltveit 2017; Lodhia 2016; Praveen 2014; Rothstein 2016; Shao 2015; Tewari 2017; Toda 2017; van der Wal 2016; Wolff‐Piggott 2018	No/very minor concerns regarding methodological limitations	Moderate concerns regarding coherence because we may be over‐interpreting health workers' perceptions	Moderate concerns regarding adequacy because some of the supporting quotes speak indirectly to the finding	No/minor concerns regarding relevance	Moderate confidence	Due to no/very minor concerns regarding methodological limitations and relevance, but moderate concerns regarding coherence and adequacy
**Finding 36** It was important for health workers that mobile health interventions be integrated with other existing electronic health information systems. This interoperability made it more likely that mobile devices would be integrated into standard care practices, while the absence of integration frustrated health workers.	Garg 2016; Ginsburg 2016; Lodhia 2016; Rothstein 2016	No/minor concerns regarding methodological limitations	No/very minor concerns regarding coherence	Moderate concerns regarding adequacy because one study had thin data, and the finding is based on only four studies	Moderate concerns regarding relevance because two of the four studies were conducted in the same country	Moderate confidence	Due to no/very minor concerns regarding methodological limitations and coherence, but moderate concerns regarding relevance and adequacy
**Finding 37** Health workers offered programmatic and implementation recommendations to improve mobile health interventions. The most cited of these was that the interventions be expanded to other settings and services, beyond what they were using it for as described in the studies. Other recommendations included raising community awareness about mHealth programmes, being involved in developing programmes, and appointing a 'mobile health champion'. Workers also suggested that those collecting surveillance data, must be informed of how the data are used.	Bacchus 2016; Barnabee 2014; Ginsburg 2016; Hao 2015; Khan 2015; Kolltveit 2017; Lodhia 2016; Madon 2014; Medhanyie 2015; Mitchell 2012; Murray 2015; Mwendwa 2016; Rothstein 2016; Schoen 2017; Toda 2017; van der Wal 2016	Moderate concerns regarding methodological limitations: though the majority of studies had no to minor methodological limitations, there were three studies with moderate limitations and two with serious limitations	No/very minor concerns regarding coherence	No/very minor concerns regarding adequacy	No/very minor concerns regarding relevance	High confidence	Due to no/very minor concerns regarding, coherence, relevance, and adequacy, and moderate concerns regarding methodological limitations
**Finding 38** Health workers had several technical recommendations to improve mobile health devices, for instance solar panels to counter poor electricity access and using photos to track clients' recovery from illness. Other recommendations included using sturdier devices, bigger screens, and having common applications, such as work scheduling on the devices.	Coetzee 2017; Henry 2016; Lodhia 2016; Praveen 2014; Quinn 2013; Schoen 2017	Minor concerns regarding methodological limitations because the majority of studies had no to minor methodological limitations	No/very minor concerns regarding coherence	Minor concerns regarding adequacy because of thin data found in the studies	Moderate concerns regarding relevance because of the limited range of contexts in which the studies were conducted	Moderate confidence	Due to no/very minor concerns regarding coherence, minor concerns regarding adequacy, and methodological limitations, and moderate concerns regarding relevance
**Finding 39** The main challenges health workers experienced in using mobile devices, were poor network connectivity, access to electricity, and the costs to recharge devices. Solutions offered, included using solar panels, using the powered‐up phone of a colleague, or reverting back to the paper‐based system. Sometimes poor connectivity resulted in client dissatisfaction because it created delays in receiving health care. Health workers' commitment to their clients motivated them to cope with these and other challenges.	Chang 2011; Ginsburg 2016; Hampshire 2016; Ilozumba 2018; Khan 2015; Lodhia 2016; Madon 2014; Mwendwa 2016; Nguyen 2015; Praveen 2014; Quinn 2013; Schoen 2017; Toda 2017; van der Wal 2016; Watkins 2018	Minor concerns regarding methodological limitations as the majority of papers had no to minor methodological limitations	No/very minor concerns regarding coherence	No/very minor concerns regarding adequacy	No/very minor concerns regarding relevance	High confidence	Due to no/very minor concerns regarding coherence, relevance, and adequacy, and minor concerns regarding methodological limitation
**Finding 40** Health workers expressed dissatisfaction with mobile devices when technology changes were too rapid, showed a dislike for typing, and were concerned that mHealth impersonalised their interaction with clients. Since these dissatisfactions were only infrequently raised within the data set, it is unclear if these perceptions reflect wider experience.	Bacchus 2016; Hao 2015; Schoen 2017; Valaitis 2005	Minor concerns regarding the methodological limitations because only one study had poorly described methods	No / very minor concerns regarding coherence	Serious concerns regarding adequacy because of a small number of studies and very thin data. In addition, each reason for dissatisfaction was only reported in one study	Moderate concerns regarding relevance because the supporting data come from a limited number of contexts	Low confidence	Due to serious concerns regarding adequacy, moderate concerns regarding relevance, minor concerns regarding methodological concerns, and no/very minor concerns regarding coherence
**Finding 41** Health workers discussed challenges, beyond network and electricity issues, that sometimes were just an annoyance or a concern, but at other times also impeded their mHealth activities, and their ability to provide a service assisted by the use mobile devices. These included damaged devices, loss and theft of devices, having to carry two devices, not being able to readily buy phone credit when needed, not being able to send long messages because of character limitations, and the limitations of the language capabilities of their devices.	Chang 2011; Cherrington 2015; Coetzee 2017; Hampshire 2016; Hao 2015; Ilozumba 2018; Lodhia 2016; Medhanyie 2015; Murray 2015; Mwendwa 2016; Praveen 2014; Quinn 2013; Rothstein 2016; Toda 2017; Valaitis 2005; van der Wal 2016	Minor concerns regarding the methodological limitations because a few studies had poor descriptions of the data collection and analysis methods	No/very minor concerns regarding coherence	Moderate concerns regarding adequacy because some studies lack depth in the discussion, and had very thin data	No/very minor concerns regarding relevance	Moderate confidence	Due to no/very minor concerns regarding coherence and relevance, minor concerns regarding methodological limitations, and moderate concerns regarding adequacy
**Finding 42** Health workers complained when the tasks asked of them in mHealth interventions were felt to be beyond their clinical capacity, and when support from higher‐level workers was absent.	Orchard 2014; Praveen 2014	Moderate concerns regarding the methodological limitations because one of the supporting studies had serious concerns because the methods were poorly described	No/very minor concerns regarding coherence	Serious concerns regarding adequacy because of the limited number of studies and thin data in one study	Serious concerns regarding relevance as the data contributing to the findings were conducted in a high‐ and a middle‐ income country, respectively. In one study, the participants were receptionists, whom we do not assume to act as health workers in general	Very low confidence	Due to serious concerns regarding relevance and adequacy, moderate concerns regarding methodological limitations, and no/very minor concerns regarding coherence

## Characteristics of studies

### Characteristics of included studies [ordered by study ID]

**Table:** Ayiasi 2015

Country	Uganda	
Resources	Low‐income country	
Data collection	Interviews and focus group discussions	
Health worker category	Lay health workers (LHWs) and healthcare professionals at health facilities	
Health issue	Maternal and child health	
mHealth system	The LHWs used mobile phones, issued to them, to consult with health professionals for advice on providing health care to their clients.	
WHO taxonomy	**2.5 Healthcare provider communication** 2.5.1 Communication from healthcare provider(s) to supervisor	
Notes	The implementation date of the mHealth programme was not reported. The qualitative study was conducted between December 2013 and March 2014.

**Table:** Bacchus 2016

Country	USA	
Resources	High‐income country	
Data collection	Interviews and non‐participant observations	
Health worker category	Home visitors	
Health issue	Intimate partner violence (IPV)	
mHealth system	Home visitors used the tablet, issued to them, to screen clients’ risk for IPV and clients watched a video on IPV on the device.	
WHO taxonomy	**2.3 Healthcare provider decision support** 2.3.3 Screen clients by risk or other health status	
Notes	The implementation date of the mHealth programme was not reported. The qualitative study was conducted between November 2013 and August 2014.

**Table:** Bardosh 2017

Country	Canada and Kenya	
Resources	High‐income and lower middle‐income countries	
Data collection	Interviews	
Health worker category	Health administers/managers and clinicians	
Health issue	Client adherence to medication and their engagement in care	
mHealth system	A two‐way short message service (SMS) system was used, in which clients received a weekly text message regarding treatment adherence. They could send text messages to the health workers, who in turn contacted clients if needed.	
WHO taxonomy	**1.1 Targeted client communication** 1.1.2 Transmit targeted health information to client based on health status or demographics	
Notes	The implementation date of the mHealth programme was not reported. The qualitative study was conducted between February and April 2016.

**Table:** Barnabee 2014

Country	Timor‐Leste, a half‐island nation, South‐East Asia	
Resources	Lower middle‐income country	
Data collection	Interviews, observations, and fieldnotes	
Health worker category	Professionally licensed midwives	
Health issue	Maternal and child health	
mHealth system	Midwives registered pregnant women on smart phones, which was issued to them. The phones were used to (i) receive notifications to follow‐up with their clients at key milestones; (ii) access a list of clients due to give birth in the following week; (iii) send batch short message service (SMS) messages to clients; and (iv) to consult with their clients. Clients received a free SMS service to request a consultation with their midwife over the phone.	
WHO taxonomy	**2.1 Client identification and registration** 2.1.2 Enrol client for health services/clinical care plan	
Notes	The mHealth study was conducted in 2013.

**Table:** Braun 2016

Country	Tanzania	
Resources	Low‐income country	
Data collection	Mixed methods: survey and interviews	
Health worker category	Lay health workers (LHWs)	
Health issue	Maternal health	
mHealth system	The LHWs received mobile phones, loaded with software to support their counseling, screening, service provision and referrals, with mobile forms for client and service data, and text message reporting and reminders.	
WHO taxonomy	**2.3 Healthcare provider decision support** 2.3.2 Provide checklist according to protocol 2.3.3 Screen clients by risk or other health status	
Notes	The mHealth study was conducted in January 2013.

**Table:** Chang 2011

Country	Uganda	
Resources	Low‐income country	
Data collection	Interviews and focus group discussions	
Health worker category	Peer health workers (PHWs) and facility staff	
Health issue	HIV/AIDS	
mHealth system	PHWs used mobile phones, issued to them, to send a text message reporting antiretroviral treatment adherence and clinical data back to a centralised database immediately after or during each home visit. They could also call a mobile phone hotline with questions or concerns.	
WHO taxonomy	**1.1 Targeted client communication** 1.1.3 Transmit targeted alerts and reminders to client(s) **2.1 Client identification and registration** 2.1.2 Enrol client for health services/clinical care plan	
Notes	The mHealth study was nested within a larger study, which was conducted from 2006 to 2008.

**Table:** Cherrington 2015

Country	USA	
Resources	High‐income country	
Data collection	Focus group discussions	
Health worker category	Lay health workers (LHWs)	
Health issue	Diabetes	
mHealth system	The LHWs used the tablets, issued to them, to send messages or questions to a healthcare professional regarding their client care.	
WHO taxonomy	**2.5 Healthcare provider communication** 2.5.1 Communication from healthcare provider(s) to supervisor	
Notes	The implementation date of the mHealth programme was not reported. The qualitative study was conducted between November 2013 and August 2014.

**Table:** Coetzee 2017

Country	South Africa	
Resources	Upper middle‐income country	
Data collection	Focus group discussions	
Health worker category	Lay health workers (LHWs)	
Health issue	Maternal and child health	
mHealth system	There were illustrative videos on the tablets, issued to them, about a range of maternal, newborn, and early childhood topics, which the LHWs played to their clients during home visits.	
WHO taxonomy	**1.1 Targeted client communication** No subclassifcation identified	
Notes	The implementation date of the mHealth programme was not reported. The qualitative study was conducted between November 2015 and May 2016.

**Table:** Garg 2016

Country	USA	
Resources	High‐income country	
Data collection	Interviews	
Health worker category	Lay health workers (LHWs), programme managers, administrative staff, and a doctor	
Health issue	General primary healthcare services	
mHealth system	The health workers operated within a routine ‘safety net’ health system, which provided health care to uninsured and low‐income clients. The workers sent mobile text messages, manually typed or automated, to clients. It is unclear if the phones were issued to the health workers.	
WHO taxonomy	**1.1 Targeted client communication** 1.1.3 Transmit targeted alerts and reminders to client(s)	
Notes	The mHealth study was conducted in 2014.

**Table:** Ginsburg 2016

Country	Ghana	
Resources	Lower middle‐income country	
Data collection	Interviews	
Health worker category	Health administrators and healthcare providers (HCPs); HCPs included healthcare assistants, community health officers, midwives, and community health nurses	
Health issue	Childhood pneumonia	
mHealth system	The HCPs used the tablet, issued to them, to screen young children. The tablet had a pulse oximetry feature and an algorithm loaded onto it.	
WHO taxonomy	**2.3 Healthcare provider decision support** 2.3.3 Screen clients by risk or other health status	
Notes	The implementation date of the mHealth programme was not reported. The qualitative study was conducted between July and September 2014.

**Table:** Hamoy 2016

Country	Philippines	
Resources	Upper middle‐income country	
Data collection	Mixed methods: surveys, focus group discussions, participant observation and review of project reports	
Health worker category	Health professionals and health managers	
Health issue	Routine services provided at rural health facilities	
mHealth system	The health workers used mobile phones to record point‐of‐care specific data on services routinely provided at the rural health facilities. This data was aggregated and presented in a dashboard for use by programme managers and policy makers.	
WHO taxonomy	**2.5 Healthcare provider communication** 2.5.2 Communication and performance feedback to healthcare provider	
Notes	The implementation date of the mHealth programme was not reported. The survey was conducted between March and May 2013.

**Table:** Hampshire 2016

Country	Ghana and Malawi	
Resources	Ghana: lower middle‐income country Malawi: low‐income country	
Data collection	Interviews	
Health worker category	Community health nurses (CHWs)	
Health issue	General primary healthcare services	
mHealth system	There was no mHealth platform developed. The health workers used their personal mobile phones for a wide range of healthcare services.	
WHO taxonomy	No classification identified	
Notes	The mHealth study was conducted between 2012 and 2015.

**Table:** Hao 2015

Country	Swaziland	
Resources	Lower middle‐income country	
Data collection	Interviews	
Health worker category	Health workers in remote clinics responsible for receiving tests results from the national laboratory	
Health issue	Laboratory tests	
mHealth system	A mobile phone with a SIM card was issued to the clinics, on which they received clients’ test results from the laboratory.	
WHO taxonomy	**2.10 Laboratory and diagnostics imaging management** 2.10.1 Transmit diagnostic result to healthcare provider	
Notes	The implementation date of the mHealth programme was not reported. The qualitative study was conducted between July and August 2012.

**Table:** Henry 2016

Country	Kenya	
Resources	Lower middle‐income country	
Data collection	Interviews and WhatsApp messages	
Health worker category	Lay health workers (LHWs) and their supervisors	
Health issue	Unclear	
mHealth system	LHWs used WhatsApp text messaging to inform their supervisors about their work, and the supervisors in turn used WhatsApp to communicate with the LHWs. It is unclear if the phones were issued to the health workers.	
WHO taxonomy	**2.5 Healthcare provider communication** 2.5.1 Communication from healthcare provider(s) to supervisor **3.1 Human resource management** 3.1.2 Monitor performance of healthcare provider(s)	
Notes	The mHealth study was conducted between August 2014 and March 2015.

**Table:** Hirsch‐Moverman 2017

Country	Lesotho	
Resources	Lower middle‐income country	
Data collection	Interviews	
Health worker category	Lay health workers (LHWs)	
Health issue	Unclear	
mHealth system	The community‐based LHWs received automated medication and appointment reminders of their clients, on their personal mobile phones. They also sent text messages to clients, and received monthly phone credit. Facility‐based LHWs used a mobile phone, issued to them, to communicate with the community‐based LHWs. Clients with access to a mobile phone, received monthly airtime to communicate with the LHWs and facility staff.	
WHO taxonomy	**1.2 Targeted client communication** 1.1.3 Transmit targeted alerts and reminders to client(s)	
Notes	The mHealth study was conducted between April 2013 and August 2015.

**Table:** Huq 2014

Country	Bangladesh	
Resources	Lower middle‐income country	
Data collection	Interviews and focus group discussions	
Health worker category	Community skilled birth attendants (CSBAs)	
Health issue	Maternal and child health	
mHealth system	CSBAs used mobile phones, issued to them, to communicate with their clients. The clients and their families could call CSBAs, free of charge, for maternal and neonatal health and emergencies. CSBAs also used the phones to call medical experts at the Solution Link Group. Both the CSBAs and Solution Link Group members could call back the mothers or others if necessary in the course of managing a condition. All these calls were either free of charge or reimbursed.	
WHO taxonomy	**2.1 Client identification and registration** 2.1.2 Enrol client for health services/clinical care plan **2.5 Healthcare provider communication** No subclassifcation identified	
Notes	The mHealth study was conducted between September 2009 and March 2011.

**Table:** Ilozumba 2018

Country	India	
Resources	Lower middle‐income country	
Data collection	Interviews and focus group discussions	
Health worker category	Lay health workers (LHWs)	
Health issue	Prevention of mother‐to‐child transmission of HIV/AIDS	
mHealth system	LHWs used mobile phones, issued to them, to register their clients on the phones. They collected key health information on the phones during client visits, and also received educational materials regarding maternal health care on the phones.	
WHO taxonomy	**2.1 Client identification and registration** 2.1.2 Enrol client for health services/clinical care plan **2.8 Healthcare provider training** 2.8.1 Provide training content to healthcare provider(s)	
Notes	The mHealth study was initiated in 2011. The qualitative study was conducted between April 2015 and November 2016.

**Table:** Jalloh‐Vos 2013

Country	Sierra Leone	
Resources	Low‐income country	
Data collection	Mixed methods: a survey and interviews	
Health worker category	Health professionals, paraprofessionals and health managers	
Health issue	Maternal and newborn health	
mHealth system	The health workers received a mobile phone that allowed them to call numbers in the closed user group network for free. The network aimed to improve health worker to health worker communication.	
WHO taxonomy	**2.5 Healthcare provider communication** 2.5.5 Peer group for healthcare providers	
Notes	The mHealth study was conducted between August 2012 and July 2013.

**Table:** Jennings 2013

Country	Kenya	
Resources	Lower middle‐income country	
Data collection	Interviews and focus group discussions	
Health worker category	Lay health workers (LHWs) and nurses	
Health issue	General primary healthcare services	
mHealth system	There was no mHealth platform developed. The health workers used their personal mobile phones to provide healthcare services related to the prevention of mother‐to‐child transmission.	
WHO taxonomy	**2.1 Client identification and registration** 2.1.2 Enrol client for health services/clinical care plan	
Notes	It is unclear when the mHealth and qualitative studies were conducted.

**Table:** Jones 2012

Country	Kenya	
Resources	Lower middle ‐income country	
Data collection	Interviews	
Health worker category	Lay health workers (LHWs)	
Health issue	Malaria	
mHealth system	LHWs received automated treatment messages for paediatric malaria case management, with motivational quotes, on their personal mobile phones.	
WHO taxonomy	**2.5 Healthcare provider communication** 2.5.4 Transmit non‐routine health event alerts to healthcare provider(s)	
Notes	The mHealth study was conducted between May and October 2009.

**Table:** Kabakyenga 2016

Country	Uganda	
Resources	Low‐income country	
Data collection	Mixed methods: household surveys, focus group discussions, and interviews	
Health worker category	Lay health workers (LHWs)	
Health issue	Children under five years old	
mHealth system	LHWs entered client information on their mobile phones following integrated community case management (iCCM) algorithm prompts during home visits.	
WHO taxonomy	**2.3 Healthcare provider decision support** 2.3.1 Provide prompts and alerts according to protocol	
Notes	It is unclear when the mHealth study was implemented. The qualitative study was conducted at the end of 2012.

**Table:** Khan 2015

Country	Bangladesh	
Resources	Lower middle‐income country	
Data collection	Interviews	
Health worker category	Lay village doctors	
Health issue	General primary healthcare services	
mHealth system	The lay village doctors used mobile phones to call trained doctors for consultation and medication prescription regarding their clients. It is unclear if the phones were issued to the health workers.	
WHO taxonomy	**2.9 Prescription and medication management** 2.9.1 Transmit or track prescription orders	
Notes	The mHealth study was conducted between 2011 and 2013.

**Table:** Knoble 2015

Country	Nepal	
Resources	Low‐income country	
Data collection	Mixed methods	
Health worker category	Mid‐level health workers	
Health issue	General health complaints from clients visiting the health facility	
mHealth system	The software loaded onto tables allowed electronic diagnostic applications in aid of clinical diagnosis. The application required the health workers to input client data and answer specific questions.	
WHO taxonomy	**2.3 Healthcare provider decision support** 2.3.1 Provide prompts and alerts according to protocol	
Notes	It is unclear when the mHealth study was implemented.

**Table:** Kolltveit 2017

Country	Norway	
Resources	High‐income country	
Data collection	Focus group discussions	
Health worker category	Nurses	
Health issue	Diabetic foot ulcers	
mHealth system	The nurses used smart phones, issued to them, to send wound assessment images and text to specialist health workers. The specialists in turn, contacted the nurses who then performed wound care in accordance with the specialist’s assessment.	
WHO taxonomy	**2.4 Telemedicine** 2.4.4 Consultations for case management between healthcare provider(s)	
Notes	The implementation date of the mHealth programme was not reported. The qualitative study was conducted between January 2014 and June 2015.

**Table:** Lodhia 2016

Country	Kenya	
Resources	Lower middle‐income country	
Data collection	Interviews	
Health worker category	Health care providers (HCPs) and decision makers in ophthalmic service provision	
Health issue	Eye care	
mHealth system	The HCPs used smart phones to perform comprehensive eye examinations.	
WHO taxonomy	**2.3 Healthcare provider decision support** 2.3.3 Screen clients by risk or other health status	
Notes	It is unclear when the mHealth and qualitative studies were conducted.

**Table:** Madon 2014

Country	Tanzania	
Resources	Low‐income country	
Data collection	Interviews, focus group discussions, and observations	
Health worker category	Lay health workers (LHWs)	
Health issue	Neglected tropical diseases	
mHealth system	LHWs used smart phones, issued to them, to record household information for disease management.	
WHO taxonomy	**4.3 Location mapping** 4.3.3 Map location of clients and households	
Notes	The mHealth study was conducted in 2011.

**Table:** Medhanyie 2015

Country	Ethiopia	
Resources	Low‐income country	
Data collection	Interviews and field notes	
Health worker category	Lay health workers (LHWs)	
Health issue	Maternal and child health	
mHealth system	LHWs used smart phones, issued to them, to record their healthcare services to clients.	
WHO taxonomy	**2.5 Healthcare provider communication** 2.5.1 Communication from healthcare provider(s) to supervisor	
Notes	It is unclear when the mHealth and qualitative studies were conducted.

**Table:** Messinger 2017

Country	Bangladesh	
Resources	Lower middle‐income country	
Data collection	Interviews	
Health worker category	Healthcare professionals (doctors, paramedics), programme staff, and lay health workers (LHWs; traditional healers, drug store sellers, village doctors, traditional birth attendants)	
Health issue	Menstrual regulation	
mHealth system	There was no mHealth platform developed. The health workers used their personal mobile phones to access healthcare information and communicate with their clients.	
WHO taxonomy	No classification identified	
Notes	The implementation date of the mHealth programme was not reported. The qualitative study was conducted between July and September 2013.

**Table:** Missal 2016

Country	India	
Resources	Low middle‐income country	
Data collection	Mixed methods: survey and interviews	
Health worker category	Lay health workers (LHWs)	
Health issue	Maternal, newborn and child health	
mHealth system	The LHWs had mobile devices onto which the software was loaded. It included voice messages and videos on issues regarding maternal, newborn, and child health that could be played during the counselling sessions with pregnant mothers.	
WHO taxonomy	**2.3 Healthcare provider decision support** 2.3.1 Provide prompts and alerts according to protocol	
Notes	It is unclear when the mHealth study was conducted.

**Table:** Mitchell 2012

Country	Tanzania	
Resources	Low‐income country	
Data collection	Interviews	
Health worker category	Health care providers (HCPs)	
Health issue	Integrated management of childhood illness (IMCI)	
mHealth system	HCPs used personal digital assistants, issued to them, to follow the IMCI protocol during client visits.	
WHO taxonomy	**2.3 Healthcare provider decision support** 2.3.2 Provide checklist according to protocol	
Notes	The mHealth study was conducted between May 2008 and December 2009.

**Table:** Modi 2015

Country	India	
Resources	Low middle‐income country	
Data collection	Interviews	
Health worker category	Lay health workers (LHWs)	
Health issue	Maternal, newborn and child health	
mHealth system	The software loaded onto mobile phones served as a job aid for the LHWs. It included visit scheduling, diagnosis and patient management, electronic decision support, as well as supervisor features.	
WHO taxonomy	**2.2 Client health records** 2.1 Longitudinal tracking of client’s health status and services received **2.3 Healthcare provider decision support** 2.3.2 Provide checklist according to protocol **2.5 Healthcare provider communication** 2.5.1 Communication from healthcare provider to supervisor	
Notes	The programme was implemented over 7 months in 2013.

**Table:** Murray 2011

Country	Scotland	
Resources	High‐income country	
Data collection	Interviews	
Health worker category	Nurses	
Health issue	Unclear	
mHealth system	Nurses used personal digital assistants (PDAs), issued to them, to record clinical assessment information while visiting clients. The platform also had decision support in the form of assessment tools with associated healthcare algorithms.	
WHO taxonomy	**2.1 Client identification and registration** 2.1.2 Enrol client for health services/clinical care plan **2.3 Healthcare provider decision support** 2.3.2 Provide checklist according to protocol	
Notes	The mHealth study was conducted between 2004 and 2006.

**Table:** Murray 2015

Country	Canada	
Resources	High‐income country	
Data collection	Interviews	
Health worker category	Professional health workers (doctors, nurses, pharmacist), administrative staff, and social worker	
Health issue	HIV/AIDS	
mHealth system	The health workers, administrative staff, and social worker, used mobile phones for bi‐directional SMS messaging with clients. It is unclear if the phones were issued to the health workers.	
WHO taxonomy	No classification identified	
Notes	The mHealth study was conducted in 2012.

**Table:** Mwendwa 2016

Country	Rwanda	
Resources	Low‐income country	
Data collection	Focus group discussions	
Health worker category	Lay health workers (LHWs)	
Health issue	Maternal and child health	
mHealth system	LHWs used mobile phones, issued to them, to record routine health care to pregnant mothers. They received automated reminders on their clients’ appointment dates. They could also send emergency alerts and receive advice on immediate action.	
WHO taxonomy	**2.1 Client identification and registration** 2.1.2 Enrol client for health services/clinical care plan **2.3 Healthcare provider decision support** 2.3.1 Provide prompts and alerts according to protocol **2.6 Referral co‐ordination** 2.6.1 Co‐ordinate emergency response and transport	
Notes	The implementation date of the mHealth programme was not reported. The qualitative study was conducted between June and July 2013.

**Table:** Nguyen 2015

Country	India	
Resources	Lower middle‐income country	
Data collection	Focus group discussions	
Health worker category	Lay health workers (LHWs), community leaders, state officials	
Health issue	Maternal and child health	
mHealth system	LHWs used mobile phones to record their healthcare services. Their data were immediately displayed in online reports consisting of data tables and dashboards. It is unclear if the phones were issued to the health workers.	
WHO taxonomy	**2.1 Client identification and registration** 2.1.2 Enrol client for health services/clinical care plan **2.5 Healthcare provider communication** 2.5.1 Communication from healthcare provider(s) to supervisor	
Notes	The implementation date of the mHealth programme was not reported. The qualitative study was conducted between October and November 2013.

**Table:** Orchard 2014

Country	Australia	
Resources	High‐income country	
Data collection	Interviews	
Health worker category	Receptionists and nurses	
Health issue	Atrial fibrillation	
mHealth system	Receptionists and nurses used iPhones, issued to them, to screen clients before their consultation with a doctor.	
WHO taxonomy	**2.3 Healthcare provider decision support** 2.3.3 Screen clients by risk or other health status	
Notes	The mHealth study was conducted between May and October 2013.

**Table:** Praveen 2014

Country	India	
Resources	Lower middle‐income country	
Data collection	Interviews	
Health worker category	Lay health workers (LHWs) and doctors	
Health issue	Cardiovascular diseases	
mHealth system	LHWs used tablets, issued to them, to screen and provide treatment advice to clients.	
WHO taxonomy	**2.3 Healthcare provider decision support** 2.3.3 Screen clients by risk or other health status	
Notes	It is unclear when the mHealth and qualitative studies were conducted.

**Table:** Quinn 2013

Country	Ireland	
Resources	High‐income country	
Data collection	Interviews and focus group discussions	
Health worker category	Public health nurses (PHNs)	
Health issue	Chronic venous ulcers	
mHealth system	The PHNs used iPhones, issued to them, to provide wound care to clients. They sent images and notes of the wound to a doctor at tertiary level. The doctor then advised the PHN on how to treat the wound.	
WHO taxonomy	**2.4 Telemedicine** 2.4.4 Consultations for case management between healthcare provider(s)	
Notes	The mHealth study was conducted between October and December 2011.

**Table:** Ramirez 2017

Country	USA	
Resources	High‐income country	
Data collection	Interviews	
Health worker category	Lay health workers (LHWs), care managers and doctor	
Health issue	Stroke survivors	
mHealth system	The LHWs used the tablets, issued to them, to screen clients during home visits. They also captured other health information that was used for treatment planning.	
WHO taxonomy	**2.3 Healthcare provider decision support** 2.3.3 Screen clients by risk or other health status	
Notes	The implementation date of the mHealth programme was not reported. The qualitative study was conducted between April and June 2015.

**Table:** Rothstein 2016

Country	Ghana	
Resources	Lower middle‐income country	
Data collection	Interviews and focus group discussions	
Health worker category	Community health nurses (CHNs), district health information officers, and district health directors	
Health issue	Maternal and child health	
mHealth system	The CHNs used mobile phones, issued to them, to support their client care. They were able to track pregnant women and mother‐infant pairs, and received reminders of upcoming and missed appointments of their clients.	
WHO taxonomy	**2.1 Client identification and registration** 2.1.2 Enrol client for health services/clinical care plan **2.3 Healthcare provider decision support** 2.3.1 Provide prompts and alerts according to protocol	
Notes	The mHealth study was conducted in 2011 and 2013.

**Table:** Schoen 2017

Country	Brazil	
Resources	Upper middle‐income country	
Data collection	Interviews and focus group discussions	
Health worker category	Lay health workers (LHWs)	
Health issue	Routine health information	
mHealth system	LHWs used mobile phones to collect health and demographic data of community members that could be used for healthcare planning. It is unclear if the phones were issued to the health workers.	
WHO taxonomy	**4.3 Location mapping** 4.3.3 Map location of clients and households	
Notes	The mHealth study was conducted between 2009 and 2015.

**Table:** Shao 2015

Country	Tanzania	
Resources	Low‐income country	
Data collection	Interviews and focus group discussions	
Health worker category	Primary health workers (PHWs)	
Health issue	Child health	
mHealth system	The PHWs used smartphones and tablets, issued to them, to collect client clinical data. This was used to follow‐up clients with problems.	
WHO taxonomy	**4.3 Location mapping** 4.3.3 Map location of clients and households	
Notes	The implementation date of the mHealth programme was not reported. The qualitative study was conducted February ‐ October 2012.

**Table:** Shieshia 2014

Country	Malawi	
Resources	Low middle‐income country	
Data collection	Mixed methods: questionnaires, inventory assessment forms, and interviews	
Health worker category	Lay health workers (LHWs)	
Health issue	Supply chain	
mHealth system	The intervention (cStock) was an SMS and web–based reporting and resupply system that was used by the LHWs to report stock data via SMS through their personal mobile phones.	
WHO taxonomy	**3.2 Supply chain management** 3.2.2 Notify stock levels of health commodities	
Notes	Baseline and follow‐up evaluations were done in May 2010 and February 2013.

**Table:** Surka 2014

Country	South Africa	
Resources	Upper middle‐income country	
Data collection	Focus group discussions	
Health worker category	Lay health workers (LHWs)	
Health issue	Cardiovascular diseases	
mHealth system	LHWs used mobile phone, issued to them, to screen clients.	
WHO taxonomy	**2.3 Healthcare provider decision support** 2.3.3 Screen clients by risk or other health status	
Notes	It is unclear when the mHealth and qualitative studies were conducted.

**Table:** Tewari 2017

Country	India	
Resources	Lower middle‐income country	
Data collection	Interviews and focus group discussions	
Health worker category	Lay health workers (LHWs) and care doctors	
Health issue	Common mental disorders	
mHealth system	LHWs and doctors use smart phones and tablets to screen, diagnose and manage clients.	
WHO taxonomy	**2.1 Client identification and registration** 2.1.2 Enrol client for health services/clinical care plan **2.3 Healthcare provider decision support** 2.3.3 Screen clients by risk or other health status	
Notes	The mHealth study was conducted between May 2014 and April 2016.

**Table:** Toda 2017

Country	Kenya	
Resources	Lower middle‐income country	
Data collection	Interviews	
Health worker category	Subcounty disease co‐ordinators, who were the first level responders for disease surveillance activities, and health workers responsible for disease surveillance reporting tasks	
Health issue	Notifiable diseases	
mHealth system	The health workers used mobile phones (issued to them), to report disease outbreaks.	
WHO taxonomy	**4.1 Data collection, management, and use** 4.1.1 Non‐routine data collection and management	
Notes	The mHealth study was conducted between 2013 and 2014.

**Table:** Valaitis 2005

Country	Canada	
Resources	High‐income country	
Data collection	Focus group discussions	
Health worker category	Public health nurses (PHNs)	
Health issue	School health	
mHealth system	PHNs use personal digital assistants (PDAs) and tablet computers (issued to them), support calendaring, contact lists, and electronic documentation to provide health care in schools.	
WHO taxonomy	**2.1 Client identification and registration** 2.1.2 Enrol client for health services/clinical care plan **2.3 Healthcare provider decision support** 2.3.1 Provide prompts and alerts according to protocol	
Notes	The mHealth study was conducted in 2002.

**Table:** van der Wal 2016

Country	Myanmar	
Resources	Lower middle‐income country	
Data collection	Interviews and focus group discussions	
Health worker category	Auxiliary midwives (AMWs) and their supervisory midwives	
Health issue	Maternal and child health	
mHealth system	The AMWs and supervisory midwives used smartphones, issued to them, for client registration and clinical decision support.	
WHO taxonomy	**2.1 Client identification and registration** 2.1.2 Enrol client for health services/clinical care plan **2.3 Healthcare provider decision support** 2.3.1 Provide prompts and alerts according to protocol	
Notes	The mHealth study was conducted in 2014.

**Table:** van Heerden 2017

Country	Kenya	
Resources	Lower middle‐income country	
Data collection	Focus group discussions	
Health worker category	Lay health workers (LHWs)	
Health issue	Maternal and child health	
mHealth system	Software was loaded onto the LHW's mobile phones, which include a child growth and development assessment function. The recorded data were stored on a central server to inform training and supervision needs, and adjustments to programme implementation.	
WHO taxonomy	**2.3 Healthcare provider decision support** 2.3.2 Provide checklist according to protocol	
Notes	The study was conducted between May and September 2014.

**Table:** Vedanthan 2015

Country	Kenya	
Resources	Lower middle‐income country	
Data collection	Focus group discussions	
Health worker category	Nurses and doctors	
Health issue	Hypertension	
mHealth system	The health workers used tablets, issued to them, to provide health care to clients. An algorithm was loaded on the tablets with appropriate prompts and reminders to guide the workers’ health care.	
WHO taxonomy	**2.1 Client identification and registration** 2.1.2 Enrol client for health services/clinical care plan **2.3 Healthcare provider decision support** 2.3.1 Provide prompts and alerts according to protocol **2.7 Health worker activity planning and scheduling** 2.7.2 Schedule healthcare provider's activities	
Notes	It is unclear when the mHealth and qualitative studies were conducted.

**Table:** Watkins 2018

Country	South Africa	
Resources	Upper middle‐income country	
Data collection	Interviews	
Health worker category	Nurses and doctors	
Health issue	Chronic illnesses, maternal and child health	
mHealth system	There was no mHealth platform developed. The health workers used their personal mobile phones to access healthcare information and to communicate with colleagues.	
WHO taxonomy	**2.5 Healthcare provider communication** 2.5.5 Peer group for healthcare providers	
Notes	The implementation date of the mHealth programme was not reported. The qualitative study was conducted between January and June 2014.

**Table:** Westergaard 2017

Country	USA	
Resources	High‐income country	
Data collection	Interviews	
Health worker category	Lay health workers (LHWs)	
Health issue	HIV/AIDS and substance use	
mHealth system	The LHWs used smart phones, issued to them, to screen clients for barriers to care and medication adherence.	
WHO taxonomy	**2.1 Client identification and registration** 2.1.2 Enrol client for health services/clinical care plan **2.3 Healthcare provider decision support** 2.3.3 Screen clients by risk or other health status	
Notes	The mHealth study was conducted between September 2013 and November 2014.

**Table:** Wolff‐Piggott 2018

Country	South Africa	
Resources	Upper middle‐income country	
Data collection	Interviews and observations	
Health worker category	Facility managers, nurses, any other staff	
Health issue	Maternal and child health	
mHealth system	Nurses used their personal mobile phones to register pregnant women and mothers with infants. Once they were registered, these women received healthcare information on their personal phones.	
WHO taxonomy	**1.1 Targeted client communication** 1.1.1 Transmit health event alerts to specific population group(s)	
Notes	The mHealth study began in August 2014 and is ongoing.	

### Characteristics of excluded studies [ordered by study ID]

**Table:**

Study	Reason for exclusion
Adokiya 2015	Wrong technology
Beauregard 2017	Mobile devices not used to deliver primary healthcare services
Beisel 2016	Wrong technology
Boddy 2009	Wrong technology
Bouskill 2018	Wrong technology
Cai 2017	Wrong participants
Campbell 2014	Not primary health care
Crilly 2019	Wrong technology
Dev 2019	Wrong participants
Diese 2018	Hypothetical use of mobile devices
Donald 2016	Wrong technology
Doyle 2012	Wrong study type
Duclos 2017	Hypothetical use of mobile devices
Duggal 2018	Hypothetical use of mobile devices
English 2016	Not primary health care
Fairbrother 2013	Wrong participants
Farrell 2016	Not primary health care
Garrett 2008	Hypothetical use of mobile devices
Golsteijn 2017	Wrong participants
Griffiths 2017	Not primary health care
Gururajan 2014	Wrong study type
Haimi 2018	Wrong technology
Halje 2016	Not primary health care
Hallberg 2018	Wrong participants
Hanna 2013	Wrong participants
Harle 2014	Wrong technology
Harris 2013	Wrong participants
Holzman 2018	Wrong technology
Johansson 2010	Not primary health care
Kuiper 2010	Wrong study type
Larsen 2012	Wrong technology
Mares 2016	Not primary health care
Mather 2015	Not primary health care
McAlearney 2004	Not primary health care
Mehta 2018	Wrong technology
Melby 2014	Wrong technology
Mirsky 2016	Wrong technology
Mukasa 2017	Wrong technology
Neville 2006	Wrong technology
Ng 2017	Wrong participants
Nygren 2014	Wrong study type
Pahwa 2018	Not primary health care
Pimmer 2014	Not primary health care
Popeski 2015	Wrong technology
Salbach 2011	Wrong technology
Sampson 2016	Wrong technology
Sanabria 2012	Wrong study type
Seward 2018	Not primary health care
Smillie 2014	Hypothetical use of mobile devices
Sureshkumar 2016	Wrong participants
Varsi 2015	Not primary health care
Verwey 2012	Wrong participants
Willcox 2015	Wrong participants
Wittmann‐Price 2012	Not primary health care
Wong 2013	Wrong technology
Wools‐Kaloustian 2009	Wrong study type

### Characteristics of studies awaiting assessment [ordered by study ID]

**Table:** Abejirinde 2018

Notes	This study explored how using mHealth for non‐invasive screening of pre‐eclampsia, gestational diabetes and anaemia at the point‐of‐care, impacted health worker‐client relationships. The study was conducted in Ghana.

**Table:** Abejirinde 2018a

Notes	This study explored the experiences and perceptions of health workers of using an mHealth integrated diagnostic kit to support antenatal care service delivery in Ghana.

**Table:** Abidi 2018

Notes	This study explored health workers' perceptions and experiences in developing and evaluating an mHealth decision support platform used by health workers to support diabetes clients. The study was conducted in Canada.

**Table:** Alam 2019

Notes	This study explored health workers' perceptions and experiences when using mHealth to provide weekly voice or text messages to pregnant women, new mothers, and family members on various aspects of maternal, neonatal, and infant health. The study was conducted in Bangladesh.

**Table:** Amoakoh 2019

Notes	This study explored the 'how and why' of an mHealth intervention to support clinical decision making by health workers of maternal and neonatal healthcare services in Ghana.

**Table:** Anastasiadou 2019

Notes	This study explored health workers' and mHealth experts' attitudes toward mHealth tools in general, and the acceptability and feasibility of a specific mHealth tool for clients with an eating disorder in Spain.

**Table:** Asgary 2019

Notes	This study explored the acceptability and feasibility of smartphone‐based training of low‐ to mid‐level health workers in cervical cancer screening. The study was conducted in Ghana.

**Table:** Austin 2020

Notes	This study explored health workers' perceptions and experiences when using mHealth to collect daily patient‐generated health data in the UK.

**Table:** Barsky 2019

Notes	This study explored health workers' perceptions and experiences when using an SMS text messaging‐based system for blood pressure and hypertension management in Canada and Tanzania.

**Table:** Bauer 2018

Notes	This study explored health workers' perceptions and experiences when using mHealth to facilitate collaborative care to support clients with mental health problems. The study was conducted in the USA.

**Table:** Bennett‐Levy 2017

Notes	This study explored health workers' perceptions and experiences when using mHealth to provide mental health services in Australia.

**Table:** Bessat 2019

Notes	This study explored health workers' perceptions and experiences when using mHealth‐based clinical decision algorithms for client management to improve the quality of care and rational use of antimicrobials. The study was conducted in India.

**Table:** Bhatt 2018

Notes	This study explored health workers' perceptions and experiences when using mHealth‐supported screening intervention for servical cancer in India.

**Table:** Bolan 2018

Notes	This study explored the feasibility, acceptability, and potential effect of mHealth on health workers' practices in basic emergency obstetric and newborn care. The study was conducted in the Democratic Republic of the Congo.

**Table:** Bonnell 2018

Notes	This study explored the feasibility of lay health workers to use mHealth for improving the identification of pregnancy‐related complications and access to care for hard‐to‐reach pregnant women. The study was conducted in the Dominican Republic.

**Table:** Boyce 2019

Notes	This study explored health workers' perceptions and experiences when using mHealth to deliver lifesaving services for children in Malawi.

**Table:** Chirambo 2019

Notes	This study explored health workers' perceptions and experiences of factors that affected the sustainability of the use of mHealth decision‐making tools in delivering healthcare services to children in Malawi.

**Table:** de Vries 2017

Notes	This study explored health workers' perceptions and experiences of factors that may influence them and clients to use mHealth for two‐way risk communication. The study was conducted in Spain, the Netherlands, and UK.

**Table:** Dharmayat 2019

Notes	This study explored the barriers and facilitators for the wider implementation of an mHealth intervention for community case management of childhood illnesses in Malawi.

**Table:** DiCarlo 2018

Notes	This study explored health workers' perceptions and experiences of a combination of interventions, which included mHealth, to decrease the attrition among HIV‐positive women who initiated prevention of mother‐to‐child transmission (PMTCT) services, and their infants, through to 6 months postpartum. The study was conducted in Kenya.

**Table:** Dodson 2019

Notes	This study explored health workers' perceptions and experiences when using mHealth‐based clinical decision support tools.

**Table:** Donaghy 2019

Notes	This study explored health workers' perceptions and experiences when using mHealth to facilitate remote consultation between health workers in Scotland.

**Table:** Dusabe‐Richards 2016

Notes	This study explored health workers' perceptions and experiences when using mHealth to provide primary healthcare services in Ethiopia.

**Table:** Ferrari 2016

Notes	This study explored health workers' perceptions and experiences when using mHealth for self‐assessment completed by clients while waiting for consultation with a health worker. The study was conducted in Canada.

**Table:** Fischer 2019

Notes	This study explored health workers' perceptions and experiences when using mHealth to provide maternal and child healthcare services in South Africa.

**Table:** Gopalakrishnan 2020

Notes	This study explored health workers' perceptions and experiences when using mHealth to record and report their daily activities in providing maternal and child healthcare services in India.

**Table:** Grant 2019

Notes	This study explored health workers' perceptions and experiences when using mHealth for blood pressure measurement in the UK.

**Table:** Hackett 2018

Notes	This study explored health workers' perceptions and experiences regarding data security and privacy in using mHealth to improve women's uptake of maternal health services in Tanzania.

**Table:** Hackett 2019

Notes	This study explored the performance and quality of care of health workers when using mHealth to provide maternal and child healthcare services in Tanzania.

**Table:** Hans 2017

Notes	This study explored health workers' perceptions and experiences when using mHealth to capture client‐reported measures to support self‐management in Canada.

**Table:** Henkemans 2018

Notes	This study explored health workers' perceptions and experiences when using mHealth for conversational support, tailored education and information on social services, to parents in the Netherlands.

**Table:** Hutchinson 2017

Notes	This study explored health workers' perceptions and experiences of using mHealth to incorporate a rapid diagnostic test for malaria, into routine practice in Tanzania.

**Table:** Ide 2019

Notes	This study explored health workers' acceptance of mHealth to provide child healthcare services in Malawi.

**Table:** Ismail 2019

Notes	This study explored health workers' perceptions and experiences when using their personal mHealth devices in delivering healthcare services in India.

**Table:** Johnson 2019

Notes	This study explored medical students' perceptions and experiences when using mHealth during a clinical practicum in rural areas in the USA.

**Table:** Kapoor 2019

Notes	This study explored health workers' perceptions and experiences when using mHealth for substance use screening and interventions in the USA.

**Table:** Kaunda‐Khangamwa 2018

Notes	This study explored health workers' perceptions and experiences when using mHealth to improve their adherence to case management guidelines for malaria and other diseases in Malawi.

**Table:** Kawakyu 2019

Notes	This study explored issues to improve health workers' experiences with using mHealth to identify cascade inefficiencies and solutions to prevent mother‐to‐child HIV transmission in Kenya and Mozambique.

**Table:** Klocek 2019

Notes	This study explored health workers' perceptions and experiences when using mHealth to provide healthcare services in the Czech Republic.

**Table:** Kolltveit 2018

Notes	This study explored health workers' perceptions and experiences when using mHealth for remote diabetes foot ulcer care in Norway.

**Table:** Kurumop 2013

Notes	This study explored the feasibility and acceptability of a text message reminder service that supported health workers to adhere to a revised malaria treatment protocol. The study was conducted in Papua New Guinea.

**Table:** Laar 2019

Notes	This study explored the feasibility of health workers' use of mHealth for the provision of maternal and child health services in rural settings in Ghana.

**Table:** Larsen‐Cooper 2015

Notes	This study explored the benefits and limitations associated with intermediaries to provide access to, and increase utilisation of, an mHealth intervention amongst people without personal phones. The study was conducted in Malawi.

**Table:** Lemay 2012

Notes	This study explored health workers' perceptions and experiences when using mHealth to improve their use and knowledge of reproductive health and HIV/AIDS. The study was conducted in Malawi.

**Table:** Lindberg 2019

Notes	This study explored health workers' perceptions and experiences of integrating mHealth into delivering home care in rural areas in South Africa.

**Table:** MacDonald 2019

Notes	This study explored contextual factors that improved the use of mHealth among healthcare workers to enhance maternal healthcare in rural Senegal.

**Table:** Maraba 2018

Notes	This study explored the feasibility, acceptability, and potential of an mHealth application to reduce the initial loss to follow‐up in a tuberculosis programme. The study was conducted in South Africa.

**Table:** Martinez‐Brockman 2019

Notes	This study explored health workers' perceptions and experiences when using mHealth in a breastfeeding peer counselling intervention in the USA.

**Table:** Mather 2019

Notes	This study explored health workers' perceptions and experiences when integrating mHealth into their daily workflow at point‐of‐care in Australia.

**Table:** McBride 2018

Notes	This study explored health workers' perceptions and experiences when using mHealth to improve access to maternal, newborn and child health services in Vietnam.

**Table:** McHenry 2018

Notes	This study explored health workers' perceptions and experiences when using mHealth for disclosure‐related counselling with HIV‐infected children and their caregivers in Kenya.

**Table:** Mengesha 2018

Notes	This study explored health workers' perceptions and experiences when using mHealth to improve the timeliness, quality, and use of health data in Ethiopia.

**Table:** Musabyimana 2018

Notes	This study explored health workers' perceptions and experiences when using mHealth to provide maternal and child health services in Rwanda.

**Table:** Nes 2018

Notes	This study explored health workers' perceptions and experiences when using mHealth to support diabetes type 2 clients.

**Table:** Ngor 2018

Notes	This study explored health workers' perceptions and experiences when using mHealth for the surveillance and early treatment of malaria in Cambodia.

**Table:** Nichols 2019

Notes	This study explored health workers' perceptions and experiences when using mHealth to improve blood pressure monitoring and facilitate adherence to antihypertensive medication. The study was conducted in Ghana.

**Table:** Orchard 2019

Notes	This study explored the perceptions and experiences of health workers who used mHealth in an atrial fibrillation screening and management programme in Australia.

**Table:** Palazuelos 2013a

Notes	This study explored health workers' perceptions and experiences when using mHealth for medicine dosing in Mexico and Guatemala.

**Table:** Patel 2018

Notes	This study explored health workers' perceptions and experiences when using mHealth to support pregnant women in Kenya.

**Table:** Pimmer 2017

Notes	This study explored health workers' perceptions and experiences when using mHealth to improve healthcare services in rural Malawi.

**Table:** Pimmer 2018

Notes	This study explored health workers' perceptions and experiences when using mHealth to access counselling information during health education sessions with clients in Malawi.

**Table:** Ramukumba 2019

Notes	This study explored health workers' perceptions and experiences when integrating mHealth into their healthcare services in South Africa.

**Table:** Rassi 2018

Notes	This study explored the feasibility, acceptability and outcomes of an mHealth intervention to increase the coverage of intermittent preventive treatment of malaria in pregnancy. The study was conducted in Uganda.

**Table:** Rose 2019

Notes	This study explored health workers' perceptions and experiences when using mHealth for the proactive assessment of obesity risks during infancy in England.

**Table:** Saleem 2018

Notes	This study explored health workers' needs when integrating mHealth with client electronic health records. The study was conducted in the USA.

**Table:** Steege 2018

Notes	This study explored health workers' perceptions and experiences when using mHealth to provide TB and maternal healthcare services in Ethiopia.

**Table:** Stålberg 2018

Notes	This study explored health workers' perceptions and experiences when using mHealth as a facilitator in healthcare situations with children.

**Table:** Terio 2019

Notes	This study explored health workers' perceptions and experiences when using mHealth to provide post‐stroke healthcare in Uganda.

**Table:** Thobias 2018

Notes	This study explored health workers' perceptions and experiences when using mHealth to improve mothers’ knowledge of reproductive and child health in Tanzania.

**Table:** Thomsen 2019

Notes	This study explored health workers' perceptions and experiences when using mHealth to improve the quality of care and potentially save the lives of mothers and newborns in Ethiopia.

**Table:** Vamos 2019

Notes	This study explored health workers' perceptions and experiences when using mHealth to promote oral health during prenatal care visits in the USA.

**Table:** Vasalampi 2017

Notes	This study explored health workers' perceptions and experiences when using mHealth to provide home‐based care services in Finland.

**Table:** Venables 2019

Notes	This study explored health workers' perceptions and experiences when using mHealth for HIV counselling and treatment adherence in Zimbabwe.

**Table:** Verwey 2014

Notes	This study explored health workers' perceptions and experiences when using mHealth to stimulate the physical activity of chronically ill clients in the Netherlands.

**Table:** Vroom 2017

Notes	This study explored health workers' perceptions and experiences when using mHealth for reporting house‐to‐house mass drug administration data for the treatment and management of lymphatic filariasis. The study was conducted in Ghana.

**Table:** Vélez 2014

Notes	This study explored health workers' perceptions and experiences when using mHealth for managing maternal client care and decision‐making, in Ghana.

**Table:** Ware 2018

Notes	This study explored health workers' perceptions and experiences regarding the sustainability and scaling‐up of an mHealth programme that was part of standard care in a specialty heart function clinic in Canada.

**Table:** Ware 2018a

Notes	This study explored health workers' perceptions and experiences when using mHealth as part of standard care in a specialty heart function clinic in Canada.

**Table:** Watson 2015

Notes	This study explored health workers' perceptions and experiences when using mHealth to provide appropriate medical treatment to reduce maternal mortality in Papua New Guinea.

**Table:** Webb 2018

Notes	This study explored health workers' perceptions and experiences when using mHealth to screen young people for mental health disorders and health compromising behaviours in Australia.

**Table:** White 2018

Notes	This study explored health workers' perceptions and experiences when using mHealth during contact investigation for tuberculosis in Uganda.

**Table:** White 2019

Notes	This study explored health workers' perceptions and experiences when using mHealth to improve the uptake and completion of pulmonary rehabilitation in the UK.

**Table:** Xiao 2019

Notes	This study explored health workers' perceptions and experiences when using mHealth for home blood pressure monitoring. The study was conducted in China.

**Table:** Zeleke 2019

Notes	This study explored health workers' perceptions and experiences with comparing mHealth with paper‐pen data capturing for demographic surveillance. The study was conducted in Ethiopia.

**Table:** Zhang 2016

Notes	This study explored health workers' perceptions and experiences about the risks of adding biomedical sensors to mHealth healthcare services. The study was conducted in Peru.

## Differences between protocol and review

In the protocol we outlined steps to conduct a framework analysis. This approach was initiated by the review authors, but none of the frameworks available were sufficiently able to capture the nuances emerging from the data. The authors therefore decided to follow a more inductive approach, using a thematic analysis, that in this instance was more suitable to the diversity of experience and perceptions emerging from the data.

## References

[CD011942-bib-0001] Mangwi Ayiasi R, Atuyambe LM, Kiguli J, Garimoi Orach C, Kolsteren P, Criel B. Use of mobile phone consultations during home visits by Community Health Workers for maternal and newborn care: community experiences from Masindi and Kiryandongo districts, Uganda. BMC Public Health 2015;15(560):1‐13. 10.1186/s12889-015-1939-3 PMC4471930 26084369

[CD011942-bib-0002] Bacchus LJ, Bullock L, Sharps P, Burnett C, Schminkey DL, Buller AM, et al. Infusing technology into perinatal home visitation in the United States for women experiencing intimate partner violence: exploring the interpretive flexibility of an mHealth intervention. Journal of Medical Internet Research 2016;18(11):e302. 10.2196/jmir.6251 PMC5133433 27856405

[CD011942-bib-0003] Bardosh KL, Murray M, Khaemba AM, Smillie K, Lester R. Operationalizing mHealth to improve patient care: a qualitative implementation science evaluation of the WelTel texting intervention in Canada and Kenya. Global Health 2017;13(1):87. 10.1186/s12992-017-0311-z PMC5717811 29208026

[CD011942-bib-0004] Barnabee G, Harrison M, Mercer M, O'Malley G. Midwives' Perceptions of an Innovative mHealth Technology's Impact on their Work and Job Satisfaction (Masters thesis). Washington (USA): University of Washington, 2014.

[CD011942-bib-0005] Braun R, Lasway C, Agarwal S, L'Engle K, Layer E, Silas L, et al. An evaluation of a family planning mobile job aid for community health workers in Tanzania. Contraception 2016;94(1):27‐33. 10.1016/j.contraception.2016.03.016 27039033

[CD011942-bib-0006] Chang LW, Kagaayi JA, Arem H, Nakigozi G, Ssempijja V, Serwadda D, et al. Impact of a mHealth intervention for peer health workers on AIDS care in rural Uganda: a mixed methods evaluation of a cluster‐randomized trial. AIDS and Behavior 2011;15(8):1776–84. 10.1007/s10461-011-9995-x PMC3265752 21739286

[CD011942-bib-0007] Cherrington AL, Agne AA, Lampkin Y, Birl A, Shelton TC, Guzman A, et al. Diabetes Connect: developing a mobile health intervention to link diabetes community health workers with primary care. Journal of Ambulatory Care Management 2015; Vol. 38, issue 4:333–45. 10.1097/JAC.0000000000000110 PMC5126964 26353025

[CD011942-bib-0008] Coetzee B, Kohrman H, Tomlinson M, Mbewu N, Roux I Le, Adam M. Community health workers' experiences of using video teaching tools during home visits: a pilot study. Health & Social Care in the Community 2017;26(2):1‐9. 10.1111/hsc.12488 PMC7534510 28872210

[CD011942-bib-0009] Garg SK, Lyles CR, Ackerman S, Handley MA, Schillinger D, Gourley G, et al. Qualitative analysis of programmatic initiatives to text patients with mobile devices in resource‐limited health systems. BMC Medical Informatics and Decision Making 2016;16(16):1‐12. 10.1186/s12911-016-0258-7 PMC4744448 26851941

[CD011942-bib-0010] Ginsburg AS, Tawiah Agyemang C, Ambler G, Delarosa J, Brunette W, Levari S, et al. mPneumonia, an innovation for diagnosing and treating childhood pneumonia in low‐resource settings: A feasibility, usability and acceptability study in Ghana. PLoS ONE 2016; Vol. 11, issue 10:e0165201. 10.1371/journal.pone.0165201 PMC5082847 27788179

[CD011942-bib-0011] Hamoy GL, Amoranto AJ, Evangelista‐Sanchez AM, Pajarillaga ED, Ongkeko Jr AM, Sylim PG, et al. Real‐time regular routine reporting for Health (R4Health): Lessons from the implementation of a large scale mobile health system for routine health services in the Philippines. Acta Medica Philippina 2016;50(4):280‐94.

[CD011942-bib-0012] Hampshire K, Porter G, Mariwah S, Munthali A, Robson E, Owusu SA, et al. Who bears the cost of 'informal mhealth'? Health‐workers' mobile phone practices and associated political‐moral economies of care in Ghana and Malawi. Health Policy and Planning 2016;0(0):1‐9. 10.1093/heapol/czw095 PMC5886236 27476501

[CD011942-bib-0013] Hao WR, Hsu YH, Chen KC, Li HC, Iqbal U, Nguyen PA, et al. LabPush: a pilot study of providing remote clinics with laboratory results via short message service (SMS) in Swaziland, Africa ‐ a qualitative study. Computer Methods and Programs in Biomedicine 2015;118:78‐83. 10.1016/j.cmpb.2014.10.005 25453385

[CD011942-bib-0014] Henry JV, Winters N, Lakati A, Oliver M, Geniets A, Mbae SM, et al. Enhancing the supervision of community health workers with WhatsApp mobile messaging: qualitative findings from 2 low‐resource settings in Kenya. Global Health: Science and Practice 2016;4(2):311–25. 10.9745/GHSP-D-15-00386 PMC4982254 27353623

[CD011942-bib-0015] Hirsch‐Moverman Y, Daftary A, Yuengling KA, Saito S, Ntoane M, Frederix K, et al. Using mHealth for HIV/TB treatment support in Lesotho: enhancing patient‐provider communication in the START study. Journal of Acquired Immune Deficiency Syndromes 2017;74(Suppl 1):S37‐43. 10.1097/QAI.0000000000001202 PMC5147041 27930610

[CD011942-bib-0016] Huq NL, Azmi AJ, Quaiyum MA, Hossain S. Toll free mobile communication: overcoming barriers in maternal and neonatal emergencies in Rural Bangladesh. Reproductive Health 2014;11(52):1‐12. 10.1186/1742-4755-11-52 PMC4107484 25015126

[CD011942-bib-0017] Ilozumba O, Dieleman M, Kraamwinkel N, Belle S Van, Chaudoury M, Broerse JE. “I am not telling. The mobile is telling”: Factors influencing the outcomes of a community health worker mHealth intervention in India. PLoS ONE 2018;13(3):e0194927. 10.1371/journal.pone.0194927 PMC5870994 29584773

[CD011942-bib-0018] Jalloh‐Vos H, Herschderfer K, Jalloh AM, Ormel H, Koning K de, Kamara SA, et al. Mobile health: Connecting managers, service providers and clients in Bombali district, Sierra Leone. Midline study report. Amsterdam: KIT 2013.

[CD011942-bib-0019] Jennings L, Ong'ech J, Simiyu R, Sirengo M, Kassaye S. Exploring the use of mobile phone technology for the enhancement of the prevention of mother‐to‐child transmission of HIV program in Nyanza, Kenya: a qualitative study. BMC Public Health 2013;13(1131):1‐9. 10.1186/1471-2458-13-1131 PMC4234194 24308409

[CD011942-bib-0020] Jones CO, Wasunna B, Sudoi R, Githinji S, Snow RW, Zurovac D. “Even if you know everything you can forget”: health worker perceptions of mobile phone text‐messaging to improve malaria case‐management in Kenya. PLoS ONE 2012;7(6):e38636. 10.1371/journal.pone.0038636 PMC3374819 22719911

[CD011942-bib-0021] Kabakyenga J, Barigye C, Brenner J, Maling S, Buchner D, Nettle‐Aquirre A, et al. A demonstration of mobile phone deployment to support the treatment of acutely ill children under five in Bushenyi district, Uganda. African Health Sciences 2016;16(1):89‐96. 10.4314/ahs.v16i1.12 PMC4915404 27358618

[CD011942-bib-0022] Khan NU, Rasheed S, Sharmin T, Ahmed T, Mahmood SS, Khatun F, et al. Experience of using mHealth to link village doctors with physicians: lessons from Chakaria, Bangladesh. BMC Medical Informatics and Decision Making 2015;15(62):1‐9. 10.1186/s12911-015-0188-9 PMC4526289 26242574

[CD011942-bib-0023] Knoble SJ, Bhusal MR. Electronic diagnostic algorithms to assist mid‐level health care workers in Nepal: a mixed‐method exploratory study. International Journal of Medical Informatics 2015;84(5):334‐40. 10.1016/j.ijmedinf.2015.01.011 25670230

[CD011942-bib-0024] Kolltveit BH, Gjengedal E, Graue M, Iversen MM, Thorne S, Kirkevold M. Conditions for success in introducing telemedicine in diabetes foot care: a qualitative inquiry. BMC Nursing 2017;16(2):1‐10. 10.1186/s12912-017-0201-y PMC5237197 28100957

[CD011942-bib-0025] Lodhia V, Karanja S. Acceptability, usability, and views on deployment of Peek, a mobile phone mHealth intervention for eye care in Kenya: qualitative study. Journal of Medical Internet Research Mhealth and Uhealth 2016;4(2):e30. 10.2196/mhealth.4746 PMC4877502 27160779

[CD011942-bib-0026] Madon S, Amaguru JO, Malecela MN. Can mobile phones help control neglected tropical diseases? Experiences from Tanzania. Social Science & Medicine 2014;102:103e110. 10.1016/j.socscimed.2013.11.036 24565147

[CD011942-bib-0027] Medhanyie AA, Moser A, Spigt M, Yebyo H, Little A, Dinant G, et al. Mobile health data collection at primary health care in Ethiopia: a feasible challenge. Journal of Clinical Epidemiology 2015;68:80‐6. 10.1016/j.jclinepi.2014.09.006 25441699

[CD011942-bib-0028] Messinger CJ, Mahmud I, Kanan S, Jahangir YT, Sarker M, Rashid SF. Utilization of mobile phones for accessing menstrual regulation services among low‐income women in Bangladesh: a qualitative analysis. Reproductive Health 2017;14(7):1‐11. 10.1186/s12978-016-0274-1 PMC5237487 28088232

[CD011942-bib-0029] Missal BP, Marandi S, Saha RK, Kisku B, MacDonald L, Desrochers R. Building Capacity to use m‐Health in Maternal, Newborn and Child Health Interventions. HealthBridge Foundation of Canada 2016.

[CD011942-bib-0030] Mitchell M, Getchell M, Nkaka M, Msellemu D, Esch J Van, Hedt‐Gauthier B. Perceived improvement in integrated management of childhood illness implementation through use of mobile technology: qualitative evidence from a pilot study in Tanzania. Journal of Health Communication 2012;17(S1):118‐27. 10.1080/10810730.2011.649105 22548605

[CD011942-bib-0031] Modi D, Gopalan R, Shah S, Venkatraman S, Desai G, Desai S, et al. Development and formative evaluation of an innovative mHealth intervention for improving coverage of community‐based maternal, newborn and child health services in rural areas of India. Global Health Action 2015;8:26769. 10.3402/gha.v8.26769 PMC4335194 25697233

[CD011942-bib-0032] Murray E, Burns J, May C, Finch T, O'Donnell C, Wallace P, et al. Why is it difficult to implement e‐health initiatives? A qualitative study. Implementation Science 2011;6(6):1‐11. 10.1186/1748-5908-6-6 PMC3038974 21244714

[CD011942-bib-0033] Murray MC, O'Shaughnessy S, Smillie K, Borek N Van, Graham R, Maan EJ. Health care providers' perspectives on a weekly text‐messaging intervention to engage HIV‐positive persons in care (WelTel BC1). AIDS Behavior 2015;19:1875–87. 10.1007/s10461-015-1151-6 26297567

[CD011942-bib-0034] Mwendwa P. Assessing the fit of RapidSMS for maternal and new‐born health: perspectives of community health workers in rural Rwanda. Development in Practice 2016;26(1):38‐51.

[CD011942-bib-0035] Nguyen LH, LeFevre AE, Jennings L, Agarwal S, Mehl G, Labrique AB, et al. Perceptions of data processes in mobile‐based versus paper‐based health information systems for maternal, newborn and child health: a qualitative study in Andhra Pradesh, India. BMJ Innovations 2015;1:167–73.

[CD011942-bib-0036] Orchard J, Freedman SB, Lowres N, Peiris D, Neubeck L. iPhone ECG screening by practice nurses and receptionists for atrial fibrillation in general practice: the GP‐SEARCH qualitative pilot study. Australian Family Physician 2014;43(5):315‐19. 24791776

[CD011942-bib-0037] Praveen D, Patel A. SMARTHealth India: development and field evaluation of a mobile clinical decision support system for cardiovascular diseases in rural India. Journal of Medical Internet Research Mhealth and Uhealth 2014;2(4):e54. 10.2196/mhealth.3568 PMC4275493 25487047

[CD011942-bib-0038] Quinn EM, Corrigan MA, O'Mullane J, Murphy D, Lehane EA, Leahy‐Warren P, et al. Clinical unity and community empowerment: the use of smartphone technology to empower community management of chronic venous ulcers through the support of a tertiary unit. PLoS ONE 2013;8(11):e78786. 10.1371/journal.pone.0078786 PMC3827111 24265716

[CD011942-bib-0039] Ramirez M, Wu S, Ryan G, Towfighi A, Vickrey BG. Using beta‐version mHealth technology for team‐based care management to support stroke prevention: an assessment of utility and challenges. JMIR Research Protocols 2017;6(5):e94. 10.2196/resprot.7106 PMC5461415 28536094

[CD011942-bib-0040] Rothstein JD, Jennings L, Moorthy A, Yang F, Gee L, Romano K, et al. Qualitative assessment of the feasibility, usability, and acceptability of a mobile client data app for community‐based maternal, neonatal, and child care in rural Ghana. International Journal of Telemedicine and Applications 2016;2016:1‐14. 10.1155/2016/2515420 PMC5192299 28070186

[CD011942-bib-0041] Schoen J, Mallett JW, Grossman‐Kahn R, Brentani A, Kaselitz E, Heisler M. Perspectives and experiences of community health workers in Brazilian primary care centers using m‐health tools in home visits with community members. Human Resources for Health 2017;15(1):71. 10.1186/s12960-017-0245-9 PMC5622566 28962569

[CD011942-bib-0042] Shao AF, Rambaud‐Althaus C, Swai N, Kahama‐Maro J, Genton B, D'Acremont V, et al. Can smartphones and tablets improve the management of childhood illness in Tanzania? A qualitative study from a primary health care worker's perspective. BMC Health Services Research 2015;15(13):1‐12. 10.1186/s12913-015-0805-4 PMC4396573 25890078

[CD011942-bib-0043] Shieshia M, Noel M, Andersson S, Felling B, Alva S, Agarwal S, et al. Strengthening community health supply chain performance through an integrated approach: Using mHealth technology and multilevel teams in Malawi. Journal of Global Health 2014;4(2):020406. 10.7189/jogh.04.020406 PMC4267094 25520796

[CD011942-bib-0044] Surka S, Edirippulige S, Steyn K, Gaziano T, Puoane T, Levitt N. Evaluating the use of mobile phone technology to enhance cardiovascular disease screening by community health workers. International Journal of Medical Informatics 2014;83(9):648‐54. 10.1016/j.ijmedinf.2014.06.008 PMC4108525 25002305

[CD011942-bib-0045] Tewari A, Kallakuri S, Devarapalli S, Jha V, Patel A, Maulik PK. Process evaluation of the systematic medical appraisal, referral and treatment (SMART) mental health project in rural India. BMC Psychiatry 2017;17(385):1‐13. 10.1186/s12888-017-1525-6 PMC5715622 29202773

[CD011942-bib-0046] Toda M, Njeru I, Zurovac D, Kareko D, O‐Tipo S, Mwau M, et al. Understanding mSOS: A qualitative study examining the implementation of a text‐messaging outbreak alert system in rural Kenya. PLoS ONE 2017;12(6):e0179408. 10.1371/journal.pone.0179408 PMC5476271 28628629

[CD011942-bib-0047] Valaitis RK, O'Mara LM. Public health nurses' perceptions of mobile computing in a school program. Computers, Informatics, Nursing 2005;23(3):153–60. 10.1097/00024665-200505000-00011 15900173

[CD011942-bib-0048] Wal R van der. Acceptance and use of mHealth Tools by Auxiliary Midwives in Myanmar: a qualitative study (Masters thesis). Montreal (Canada): University of Montreal, 2016.

[CD011942-bib-0049] Heerden A van, Sen D, Desmond C, Louw J, Richter L. App‐supported promotion of child growth and development by community health workers in Kenya: feasibility and acceptability study. Journal of Medical Internet Research mHealth and uHealth 2017;5(12):e182. 10.2196/mhealth.6911 PMC5736876 29208588

[CD011942-bib-0050] Vedanthan R, Blank E, Tuikong N, Kamano J, Misoi L, Tulienge D, et al. Usability and feasibility of a tablet‐based Decision‐Support and Integrated Record‐keeping (DESIRE) tool in the nurse management of hypertension in rural western Kenya. International Journal of Medical Informatics 2015;83(4):207–19. 10.1016/j.ijmedinf.2014.12.005 PMC4314432 25612791

[CD011942-bib-0051] Watkins JO, Goudge J, Gómez‐Olivé FX, Griffiths F. Mobile phone use among patients and health workers to enhance primary healthcare: A qualitative study in rural South Africa. Social Science & Medicine 2018;198:139–47. 10.1016/j.socscimed.2018.01.011 29335160

[CD011942-bib-0052] Westergaard RP, Genz A, Panico K, Surkan PJ, Keruly J, Hutton HE, et al. Acceptability of a mobile health intervention to enhance HIV care coordination for patients with substance use disorders. Addiction Science & Clinical Practice 2017;12(11):2‐9. 10.1186/s13722-017-0076-y PMC5405459 28441962

[CD011942-bib-0053] Wolff‐Piggott B, Coleman J, Rivett U. The clinic‐level perspective on mHealth implementation: a South African case study. Information Technology for Development 2018;24(3):532‐53.

[CD011942-bib-0054] Adokiya MN, Awoonor‐Williams JK, Beiersmann C, Müller O. The integrated disease surveillance and response system in northern Ghana: challenges to the core and support functions. BMC Health Services Research 2015;15(288):1‐11. 10.1186/s12913-015-0960-7 PMC4515924 26216356

[CD011942-bib-0055] Beauregard P, Arnaert A, Ponzoni N. Nursing students' perceptions of using smartphones in the community practicum: A qualitative study. Nurse Education Today 2017;53:1‐6. 10.1016/j.nedt.2017.03.002 28324823

[CD011942-bib-0056] Beisel U, Umlauf R, Hutchinson E, Chandler CI. The complexities of simple technologies: re‐imagining the role of rapid diagnostic tests in malaria control efforts. Malaria Journal 2016;15(64):1‐9. 10.1186/s12936-016-1083-2 PMC4743404 26850000

[CD011942-bib-0057] Boddy D, King G, Clark JS, Heaney D, Mair F. The influence of context and process when implementing e‐health. BMC Medical Informatics and Decision Making 2009;9(9):1‐9. 10.1186/1472-6947-9-9 PMC2642812 19183479

[CD011942-bib-0058] Bouskill K, Smith‐Morris C, Bresnick G, Cuadros J, Pedersen ER. Blind spots in telemedicine: a qualitative study of staff workarounds to resolve gaps in diabetes management. BMC Health Services Research 2018;18(617):1‐9. 10.1186/s12913-018-3427-9 PMC6081904 30086743

[CD011942-bib-0059] Cai RA, Beste D. Developing and evaluating JIApp: acceptability and usability of a smartphone app system to improve self‐management in young People with Juvenile Idiopathic Arthritis. Journal of Medical Internet Research Mhealth and Uhealth 2017;5:e121. 10.2196/mhealth.7229 PMC5575419 28811270

[CD011942-bib-0060] Campbell N, Schiffer E, Buxbaum A, McLean E, Perry C, Sullivan TM. Taking knowledge for health the extra mile: participatory evaluation of a mobile phone intervention for community health workers in Malawi. Global Health Science Practice 2014;2(1):23‐34. 10.9745/GHSP-D-13-00141 PMC4168601 25276560

[CD011942-bib-0061] Crilly P, Hassanali W, Khanna G, Matharu K, Patel D, Patel D, et al. Community pharmacist perceptions of their role and the use of social media and mobile health applications as tools in public health. Research in Social and Administrative Pharmacy 2019;15(1):23‐30. 10.1016/j.sapharm.2018.02.005 29501431

[CD011942-bib-0062] Dev R, Woods NF, Unger JA, Kinuthia J, Matemo D, Farid S, et al. Acceptability, feasibility and utility of a mobile health family planning decision aid for postpartum women in Kenya. Reproductive Health 2019;16(97):1‐11. 10.1186/s12978-019-0767-9 PMC6615081 31286989

[CD011942-bib-0063] Diese M, Kalonji A, Izale B, Villeneuve S, Kintaudi NM, Clarysse G, et al. Community‐based maternal, newborn, and child health surveillance: perceptions and attitudes of local stakeholders towards using mobile phone by village health volunteers in the Kenge Health Zone, Democratic Republic of Congo. BMC Public Health 2018;18(316):1‐12. 10.1186/s12889-018-5186-2 PMC5838964 29506500

[CD011942-bib-0064] Donald M, McBrien K, Jackson W, Manns BJ, Tonelli M, King‐Shier K, et al. Development and implementation of an online clinical pathway for adult chronic kidney disease in primary care: a mixed methods study. BMC Medical Informatics and Decision Making 2016;16(109):1‐11. 10.1186/s12911-016-0350-z PMC4989366 27535555

[CD011942-bib-0065] Doyle RJ, Wang N, Anthony D, Borkan J, Shield RR, Goldman RE. Computers in the examination room and the electronic health record: physicians' perceived impact on clinical encounters before and after full installation and implementation. Family Practice 2012;29:601‐8. 10.1093/fampra/cms015 22379185

[CD011942-bib-0066] Duclos V, Yé M, Moubassira K, Sanou H, Sawadogo NH, Bibeau G, et al. Situating mobile health: a qualitative study of mHealth expectations in the rural health district of Nouna, Burkina Faso. Health Research Policy and Systems 2017;15 Suppl 1:1‐13. 10.1186/s12961-017-0211-y PMC5516845 28722558

[CD011942-bib-0067] Duggal M, Chakrapani V, Liberti L, Satyanarayna V, Varghese M, Singh P, et al. Acceptability of mobile phone‐based nurse‐delivered counseling intervention to improve HIV treatment adherence and self‐care behaviors among HIV‐positive women in India. AIDS Patient Care STDS 2018;32:349‐59. 10.1089/apc.2017.0315 PMC6121177 30179531

[CD011942-bib-0068] English LL, Dunsmuir D. The PAediatric Risk Assessment (PARA) mobile app to reduce postdischarge child mortality: design, usability, and feasibility for health care workers in Uganda. Journal of Medical Internet Research mHealth and uHealth 2016;4(1):e16. 10.2196/mhealth.5167 PMC4771927 26879041

[CD011942-bib-0069] Fairbrother P, Pinnock H, Hanley J, McCloughan L, Sheikh A, Pagliari C, et al. Exploring telemonitoring and self‐management by patients with chronic obstructive pulmonary disease: a qualitative study embedded in a randomized controlled trial. Patient Education and Counseling 2013;93(3):403‐10. 10.1016/j.pec.2013.04.003 23647981

[CD011942-bib-0070] Farrell M. Use of iphones by nurses in an acute care setting to improve communication and decision‐making processes: aualitative analysis of nurses' perspectives on iphone use. Journal of Medical Internet Research mHealth and uHealth 2016;4(2):e43. 10.2196/mhealth.5071 PMC4908301 27246197

[CD011942-bib-0071] Garrett B, Klein G. Value of wireless personal digital assistants for practice: perceptions of advanced practice nurses. Journal of Clinical Nursing 2008;17(16):2146‐54. 10.1111/j.1365-2702.2008.02351.x 18705736

[CD011942-bib-0072] Golsteijn RH, Bolman C, Volders E, Peels DA, Vries H de, Lechner L. Development of a computer‐tailored physical activity intervention for prostate and colorectal cancer patients and survivors: OncoActive. BMC Cancer 2017;17(446):1‐20. 10.1186/s12885-017-3397-z PMC5485671 28651586

[CD011942-bib-0073] Griffiths F, Bryce C, Cave J, Dritsaki M, Fraser J, Hamilton K, et al. Timely digital patient‐clinician communication in specialist clinical services for young people: a mixed‐methods study (The LYNC Study). Journal of Medical Internet Research 2017;19(4):e102. 10.2196/jmir.7154 PMC5404145 28396301

[CD011942-bib-0074] Gururajan R, Hafeez‐Baig Ab. An empirical study to determine factors that motivate and limit the implementation of ICT in healthcare environments. BMC Medical Informatics and Decision Making 2014;14(98):1‐8. 10.1186/1472-6947-14-98 PMC4391394 25540040

[CD011942-bib-0075] Haimi M, Brammli‐Greenberg S, Waisman Y, Baron‐Epel O. Physicians' experiences, attitudes and challenges in a pediatric telemedicine service. Pediatric Research 2018;84(5):650‐6. 10.1038/s41390-018-0117-6 30120402

[CD011942-bib-0076] Halje K, Timpka T, Ekberg J, Bång M, Fröberg A, Eriksson H. Towards mHealth systems for support of psychotherapeutic practice: a qualitative study of researcher‐clinician collaboration in system design and evaluation. International Journal of Telemedicine and Applications 2016;2016:1‐7. 10.1155/2016/5151793 PMC4791500 27034661

[CD011942-bib-0077] Hallberg I, Ranerup A, Bengtsson U, Kjellgren K. Experiences, expectations and challenges of an interactive mobile phone‐based system to support self‐management of hypertension: patients' and professionals' perspectives. Patient Preference and Adherence 2018;12:467‐76. 10.2147/PPA.S157658 PMC5885974 29643739

[CD011942-bib-0078] Hanna L, Fairhurst K. Using information and communication technologies to consult with patients in Victorian primary care: the views of general practitioners. Australian Journal of Primary Health 2013;19(2):166‐70. 10.1071/PY11153 22950961

[CD011942-bib-0079] Harle C, Cook R, Fillingim R. Informing clinical decision support for chronic pain in primary care: how do physicians decide when to initiate opioids?. Journal of Pain 2014;15(4):S87.

[CD011942-bib-0080] Harris JL, Furberg R, Martin N, Kuhns L, Lewis MA, Coomes C, et al. Implementing an SMS‐based intervention for persons living with human immunodeficiency virus. Journal of Public Health Management and Practice 2013;19(2):E9‐16. 10.1097/PHH.0b013e3182582b59 23358304

[CD011942-bib-0081] Holzman SB, Zenilman A, Shah M. Advancing patient‐centered care in tuberculosis management: a mixed‐methods appraisal of video directly observed therapy. Open Forum Infectious Diseases 2018;5(4):1‐8. 10.1093/ofid/ofy046 PMC5917780 29732378

[CD011942-bib-0082] Johansson PE, Petersson GI, Nilsson GC. Personal digital assistant with a barcode reader ‐ a medical decision support system for nurses in home care. International Journal of Medical Informatics 2010;79(4):232‐42. 10.1016/j.ijmedinf.2010.01.004 20138577

[CD011942-bib-0083] Kuiper R. Metacognitive factors that impact student nurse use of point of care technology in clinical settings. International Journal of Nursing Education Scholarship 2010;7(1):1‐15. 10.2202/1548-923X.1866 20196764

[CD011942-bib-0084] Larsen AC. Trappings of technology: casting palliative care nursing as legal relations. Nursing Inquiry 2012;19(4):334‐44. 10.1111/j.1440-1800.2011.00568.x 23134278

[CD011942-bib-0085] Mares ML, Gustafson DH, Glass JE, Quanbeck A, McDowell H, McTavish F, et al. Implementing an mHealth system for substance use disorders in primary care: a mixed methods study of clinicians’ initial expectations and first year experiences. BMC Medical Informatics and Decision Making 2016;16(126):1‐12. 10.1186/s12911-016-0365-5 PMC5043521 27687632

[CD011942-bib-0086] Mather C, Cummings E. Unveiling the mobile learning paradox. Studies in Health Technology and Informatics 2015;218:126‐31. 26262539

[CD011942-bib-0087] McAlearney AS, Schweikhart SB, Medow MA. Doctors' experience with handheld computers in clinical practice: qualitative study. BMJ (Clinical research ed.) 2004;328(7449):1‐5. 10.1136/bmj.328.7449.1162 PMC411090 15142920

[CD011942-bib-0088] Mehta K, Kumar AM. 'M‐TRACK' (mobile phone reminders and electronic tracking tool) cuts the risk of pre‐treatment loss to follow‐up by 80% among people living with HIV under programme settings: a mixed‐methods study from Gujarat, India. Global Health Action 2018;11(1):1‐11. 10.1080/16549716.2018.1438239 PMC5827770 29482468

[CD011942-bib-0089] Melby L, Helleso R. Introducing electronic messaging in Norwegian healthcare: unintended consequences for interprofessional collaboration. International Journal of Medical Informatics 2014;83(5):343‐53. 10.1016/j.ijmedinf.2014.02.001 24636700

[CD011942-bib-0090] Mirsky JB, Tieu L, Lyles C, Sarkar U. A mixed‐methods study of patient‐provider e‐mail content in a safety‐net setting. Journal of Health Communication 2016;21(1):85‐91. 10.1080/10810730.2015.1033118 PMC5431571 26332306

[CD011942-bib-0091] Mukasa O, Mushi HP, Maire N. Do surveys with paper and electronic devices differ in quality and cost? Experience from the Rufiji Health and demographic surveillance system in Tanzania. Global Health Action 2017;10(1):1‐10. 10.1080/16549716.2017.1387984 PMC5700525 29157182

[CD011942-bib-0092] Neville RG, Greene AC, Lewis S. Patient and health care professional views and experiences of computer agent‐supported health care. Journal of Innovation in Health Informatics 2006;14(1):11‐5. 10.14236/jhi.v14i1.610 16848962

[CD011942-bib-0093] Ng SL, Phelan S, Leonard M, Galster J. A qualitative case study of smartphone‐connected hearing aids: influences on patients, clinicians, and patient‐clinician interactions. Journal of the American Academy of Audiology 2017;28(6):506‐21. 10.3766/jaaa.15153 28590895

[CD011942-bib-0094] Nygren D, Isaksson AL. Battling malaria in rural Zambia with modern technology: a qualitative study on the value of cell phones, geographical information systems, asymptomatic carriers and rapid diagnostic tests to identify, treat and control malaria. Journal of Public Health in Africa 2014;5(1):9‐13. 10.4081/jphia.2014.171 PMC5345455 28299110

[CD011942-bib-0095] Pahwa P, Lunsford S. Experiences of Indian health workers using WhatsApp for improving aseptic practices with newborns: exploratory qualitative study. Journal of Medical Internet Research Medical Informatics 2018;6(1):e13. 10.2196/medinform.8154 PMC5856937 29496651

[CD011942-bib-0096] Pimmer C, Brysiewicz P, Linxen S, Walters F, Chipps J, Grohbiel U. Informal mobile learning in nurse education and practice in remote areas‐‐a case study from rural South Africa. Nurse Education Today 2014;34(11):1398‐404. 10.1016/j.nedt.2014.03.013 24745478

[CD011942-bib-0097] Popeski N, McKeen C, Khokhar B, Edwards A, Ghali WA, Sargious P, et al. Perceived barriers to and facilitators of patient‐to‐provider e‐mail in the management of diabetes care. Canadian Journal of Diabetes 2015;39(6):478‐83. 10.1016/j.jcjd.2015.07.001 26409770

[CD011942-bib-0098] Salbach NM, Veinot P, Jaglal SB, Bayley M, Rolfe D. From continuing education to personal digital assistants: what do physical therapists need to support evidence‐based practice in stroke management?. Journal of Evaluation in Clinical Practice 2011;17(4):786‐93. 10.1111/j.1365-2753.2010.01456.x 21040248

[CD011942-bib-0099] Sampson R, Barbour R, Wilson P. Email communication at the medical primary‐secondary care interface: a qualitative exploration. British Journal of General Practice 2016;66(648):e467‐73. 10.3399/bjgp16X685273 PMC4917049 27162209

[CD011942-bib-0100] Sanabria TJ, Orta M. The Maniapure Program ‐ lessons learned from a rural experience: two decades delivering primary healthcare through telemedicine. Telemedicine Journal and eHealth 2012;18(7):1‐8. 10.1089/tmj.2011.0192 22823209

[CD011942-bib-0101] Seward MW, Simon D, Richardson M, Oken E, Gillman MW, Hivert MF. Supporting healthful lifestyles during pregnancy: a health coach intervention pilot study. BMC Pregnancy and Childbirth 2018;18(375):1‐11. 10.1186/s12884-018-2010-z PMC6142676 30223779

[CD011942-bib-0102] Smillie K, Borek N Van, Kop ML van der, Lukhwaro A, Li N, Karanja S, et al. Mobile health for early retention in HIV care: a qualitative study in Kenya (WelTel Retain). African Journal of AIDS Research 2014;13(4):331‐8. 10.2989/16085906.2014.961939 PMC5585007 25555099

[CD011942-bib-0103] Sureshkumar K, Murthy GV, Natarajan S, Naveen C, Goenka S, Kuper H. Evaluation of the feasibility and acceptability of the ‘Care for Stroke’ intervention in India, a smartphone‐enabled, carer‐supported, educational intervention for management of disability following stroke. BMJ Open 2016;6(2):e009243. 10.1136/bmjopen-2015-009243 PMC4746451 26839011

[CD011942-bib-0104] Varsi C, Ekstedt M, Gammon D, Borosund E, Ruland CM. Middle managers' experiences and role in implementing an interactive tailored patient assessment eHealth intervention in clinical practice. Computers, Informatics, Nursing: CIN 2015;33(6):249‐57. 10.1097/CIN.0000000000000158 25988851

[CD011942-bib-0105] Verwey R, Weegen S van der, Tange H, Spreeuwenberg M, Weijden T van der, Witte L de. Get moving: the practice nurse is watching you! A case study of the user‐centred design process and testing of a web‐based coaching system to stimulate the physical activity of chronically ill patients in primary care. Informatics in Primary Care 2012;20(4):289‐98. 10.14236/jhi.v20i4.19 23890341

[CD011942-bib-0106] Willcox JC, Pligt P van der. Views of women and health professionals on mHealth lifestyle interventions in pregnancy: a qualitative investigation. Journal of Medical Internet Research mHealth and uHealth 2015;3(4):e99. 10.2196/mhealth.4869 PMC4704935 26510886

[CD011942-bib-0107] Wittmann‐Price RA, Kennedy LD, Godwin C. Use of personal phones by senior nursing students to access health care information during clinical education: staff nurses' and students' perceptions. Journal of Nursing Educaction 2012;51(11):642‐6. 10.3928/01484834-20120914-04 22978275

[CD011942-bib-0108] Wong MC, Cummings E, Turner P. User‐centered design in clinical handover: exploring post‐implementation outcomes for clinicians. Studies in Health Technololgy and Informatics 2013;192:253‐7. 23920555

[CD011942-bib-0109] Wools‐Kaloustian KK, Sidle JE, Selke HM, Vedanthan R, Kemboi EK, Boit LJ, et al. A model for extending antiretroviral care beyond the rural health centre. Journal of the International AIDS Society 2009;12(22):1‐11. 10.1186/1758-2652-12-22 PMC2762459 19788755

[CD011942-bib-0110] Abejirinde IO, Douwes R, Bardaji A, Abugnaba‐Abanga R, Zweekhorst M, Roosmalen J van, et al. Pregnant women's experiences with an integrated diagnostic and decision support device for antenatal care in Ghana. BMC Pregnancy Childbirth 2018;18(1):1‐11. 10.1186/s12884-018-1853-7 PMC5989381 29871596

[CD011942-bib-0111] Abejirinde IO, Zweekhorst M, Bardaji A, Abugnaba‐Abanga R, Apentibadek N, Brouwere V De, et al. Unveiling the black box of diagnostic and clinical decision support systems for antenatal care: realist evaluation. Journal of Medical Internet Research mHealth and uHealth 2018;6(12):e11468. 10.2196/11468 PMC6320439 30578177

[CD011942-bib-0112] Abidi S, Vallis M, Piccinini‐Vallis H, Imran SA, Abidi SS. Diabetes‐related behavior change knowledge transfer to primary care practitioners and patients: implementation and evaluation of a digital health platform. Journal of Medical Internet Research Medical Informatics 2018;6(2):e25. 10.2196/medinform.9629 PMC5932333 29669705

[CD011942-bib-0113] Alam M, Banwell C, Olsen A, Lokuge K. Patients' and doctors' perceptions of a mobile phone‐based consultation service for maternal, neonatal, and infant health care in Bangladesh: a mixed‐methods study. Journal of Medical Internet Research mHealth and uHealth 2019;7(4):e11842. 10.2196/11842 PMC6658262 31008716

[CD011942-bib-0114] Amoakoh HB, Klipstein‐Grobusch K, Ansah EK, Grobbee DE, Yveoo L, Agyepong I. How and why front‐line health workers (did not) use a multifaceted mHealth intervention to support maternal and neonatal healthcare decision‐making in Ghana. BMJ Global Health 2019;4(2):e001153. 10.1136/bmjgh-2018-001153 PMC6441261 30997162

[CD011942-bib-0115] Anastasiadou D, Folkvord F, Serrano‐Troncoso E, Lupianez‐Villanueva F. Mobile health adoption in mental health: user experience of a mobile health app for patients with an eating disorder. Journal of Medical Internet Research mHealth and uHealth 2019;7(6):e12920. 10.2196/12920 PMC6592393 31199329

[CD011942-bib-0116] Asgary R, Cole H, Adongo P, Nwameme A, Maya E, Adu‐Amankwah A, et al. Acceptability and implementation challenges of smartphone‐based training of community health nurses for visual inspection with acetic acid in Ghana: mHealth and cervical cancer screening. BMJ Open 2019;9(7):e030528. 10.1136/bmjopen-2019-030528 PMC6661590 31315879

[CD011942-bib-0117] Austin L, Sharp CA, Veer SN van der, Machin M, Humphreys J, Mellor P, et al. Providing 'the bigger picture': benefits and feasibility of integrating remote monitoring from smartphones into the electronic health record. Rheumatology 2020;59(2):367‐78. 10.1093/rheumatology/kez207 PMC7223265 31335942

[CD011942-bib-0118] Barsky J, Hunter R, McAllister C, Yeates K, Campbell N, Liu P, et al. Analysis of the implementation, user perspectives, and feedback from a mobile health intervention for individuals living with hypertension (DREAM‐GLOBAL): mixed methods study. Journal of Medical Internet Research mHealth and uHealth 2019;7(12):e12639. 10.2196/12639 PMC6928701 31815678

[CD011942-bib-0119] Bauer AM, Iles‐Shih M, Ghomi RH, Rue T, Grover T, Kincler N, et al. Acceptability of mHealth augmentation of collaborative care: a mixed methods pilot study. General Hospital Psychiatry 2018;51:22‐9. 10.1016/j.genhosppsych.2017.11.010 PMC6512981 29272712

[CD011942-bib-0120] Bennett‐Levy J, Singer J, DuBois S, Hyde K. Translating e‐mental health into practice: what are the barriers and enablers to e‐mental health implementation by Aboriginal and Torres Strait Islander health professionals?. Journal of Medical Internet Research 2017;19(1):e1. 10.2196/jmir.6269 PMC5266824 28077347

[CD011942-bib-0121] Bessat C, Zonon NA, D'Acremont V. Large‐scale implementation of electronic integrated management of childhood illness (eIMCI) at the primary care level in Burkina Faso: a qualitative study on health worker perception of its medical content, usability and impact on antibiotic prescription and resistance. BMC Public Health 2019;19(449):1‐12. 10.1186/s12889-019-6692-6 PMC6489291 31035968

[CD011942-bib-0122] Bhatt S, Isaac R, Finkel M, Evans J, Grant L, Paul B, et al. Mobile technology and cancer screening: lessons from rural India. Journal of Global Health 2018;8(2):1‐9. 10.7189/jogh.08.020421 PMC6304168 30603075

[CD011942-bib-0123] Bolan NE, Sthreshley L, Ngoy B, Ledy F, Ntayingi M, Makasy D, et al. mLearning in the Democratic Republic of the Congo: a mixed‐methods feasibility and pilot cluster randomized trial using the safe delivery app. Global Health: Science and Practice 2018;6(4):693‐710. 10.9745/GHSP-D-18-00275 PMC6370362 30591577

[CD011942-bib-0124] Bonnell S, Griggs A, Avila G, Mack J, Bush RA, Vignato J, et al. Community health workers and use of mHealth: improving identification of pregnancy complications and access to care in the Dominican Republic. Health Promotion Practice 2018;19(3):331‐40. 10.1177/1524839917708795 28578606

[CD011942-bib-0125] Boyce SP, Nyangara F, Kamunyori J. A mixed‐methods quasi‐experimental evaluation of a mobile health application and quality of care in the integrated community case management program in Malawi. Journal of Global Health 2019;9(1):1‐13. 10.7189/jogh.09.010811 PMC6594718 31263554

[CD011942-bib-0126] Chirambo GB, Muula AS, Thompson M. Factors affecting sustainability of mHealth decision support tools and mHealth technologies in Malawi. Informatics in Medicine Unlocked 2019;17:1‐7.

[CD011942-bib-0127] Vries ST de, Wong L, Sutcliffe A, Houÿez F, Lasheras RC, Peter GM, et al. Factors influencing the use of a mobile app for reporting adverse drug reactions and receiving safety information: a qualitative study. Drug Safety 2017;40(5):443‐55. 10.1007/s40264-016-0494-x PMC5384960 28035492

[CD011942-bib-0128] Dharmayat KI, Tran T, Hardy V, Chirambo BG, Thompson MJ, Ide N, et al. Sustainability of ‘mhealth’ interventions in sub‐Saharan Africa: a stakeholder analysis of an electronic community case management project in Malawi. Malawi Medical Journal 2019;31(3):177‐83. 10.4314/mmj.v31i3.3 PMC6895377 31839886

[CD011942-bib-0129] DiCarlo A, Fayorsey R, Syengo M, Chege D, Sirengo M, Reidy W, et al. Lay health worker experiences administering a multi‐level combination intervention to improve PMTCT retention. BMC Health Services Research 2018;18(17):1‐13. 10.1186/s12913-017-2825-8 PMC5763814 29321026

[CD011942-bib-0130] Dodson CH, Baker E, Bost K. Thematic analysis of nurse practitioners use of clinical decision support tools and clinical mobile apps for prescriptive purposes. Journal of the American Association of Nurse Practitioners 2019;31(9):522‐6. 10.1097/JXX.0000000000000170 30829976

[CD011942-bib-0131] Donaghy E, Atherton H, Hammersley V, McNeilly H, Bikker A, Robbins L, et al. Acceptability, benefits, and challenges of video consulting: a qualitative study in primary care. British Journal of General Practice 2019;69(686):e586‐94. 10.3399/bjgp19X704141 PMC6617540 31160368

[CD011942-bib-0132] Dusabe‐Richards JN, Tesfaye HT, Mekonnen J, Kea A, Theobald S, Datiko DG. Women health extension workers: capacities, opportunities and challenges to use eHealth to strengthen equitable health systems in Southern Ethiopia. Canadian Journal of Public Health 2016;107(4‐5):e355‐61. 10.17269/CJPH.107.5569 PMC6972369 28026697

[CD011942-bib-0133] Ferrari M, Ahmad F, Shakya Y, Ledwos C, McKenzie K. Computer‐assisted client assessment survey for mental health: patient and health provider perspectives. BMC Health Services Research 2016;16(516):1‐15. 10.1186/s12913-016-1756-0 PMC5035495 27663508

[CD011942-bib-0134] Fischer AE, Sebidi J, Barron P, Lalla‐Edward ST. The MomConnect nurses and midwives support platform (NurseConnect): a qualitative process evaluation. Journal of Medical Internet Research mHealth and uHealth 2019;7(2):e11644. 10.2196/11644 PMC6391642 30758298

[CD011942-bib-0135] Gopalakrishnan L, Buback L, Fernald L, Walker D, Diamond‐Smith N. Using mHealth to improve health care delivery in India: a qualitative examination of the perspectives of community health workers and beneficiaries. PLoS ONE 2020;15(1):e0227451. 10.1371/journal.pone.0227451 PMC6961923 31940326

[CD011942-bib-0136] Grant S, Hodgkinson J, Schwartz C, Bradburn P, Franssen M, Hobbs FD, et al. Using mHealth for the management of hypertension in UK primary care: an embedded qualitative study of the TASMINH4 randomised controlled trial. British Journal of General Practice 2019;69(686):e612‐20. 10.3399/bjgp19X704585 PMC6607844 31262847

[CD011942-bib-0137] Hackett KM, Kazemi M, Sellen DW. Keeping secrets in the cloud: mobile phones, data security and privacy within the context of pregnancy and childbirth in Tanzania. Social Sciences & Medicine 2018;211(2018):190‐7. 10.1016/j.socscimed.2018.06.014 29960170

[CD011942-bib-0138] Hackett K, Kazemi M, Lafleur C, Nyella P, Godfrey L, Sellen D. 'It makes you someone who changes with the times': health worker and client perspectives on a smartphone‐based counselling application deployed in rural Tanzania. Health Policy Plan 2019;34(4):307‐15. 10.1093/heapol/czz036 31155655

[CD011942-bib-0139] Hans PK, Gray CS, Gill A, Tiessen J. The provider perspective: investigating the effect of the electronic patient‐reported outcome (ePRO) mobile application and portal on primary care provider workflow. Primary Health Care Research & Development 2017;19(2):151‐64. 10.1017/S1463423617000573 PMC6452954 28899449

[CD011942-bib-0140] Henkemans BO, Keij M, Grootjen M, Kamphuis M, Dijkshoorn A. Design and evaluation of the StartingTogether app for home visits in preventive child health care. BMC Nursing 2018;17(41):1‐16. 10.1186/s12912-018-0310-2 PMC6139149 30237751

[CD011942-bib-0141] Hutchinson E, Reyburn H, Hamlyn E, Long K, Meta J, Mbakilwa H, et al. Bringing the state into the clinic? Incorporating the rapid diagnostic test for malaria into routine practice in Tanzanian primary healthcare facilities. Global Public Health 2017;12(9):1077‐91. 10.1080/17441692.2015.1091025 PMC5526135 26457440

[CD011942-bib-0142] Ide N, Hardy V, Chirambo G, Heavin C, O’connor Y, O’donoghue J, et al. People welcomed this innovation with two hands: A qualitative report of an mhealth intervention for community case management in Malawi. Annals of Global Health 2019;85(1):1‐10. 10.5334/aogh.919 PMC6634480 31025838

[CD011942-bib-0143] Ismail A, Kumar N. Empowerment on the margins: the online experiences of community health workers. CHI Conference on Human Factors in Computing Systems Proceedings, Glasgow, Scotland, UK. Glasgow, Scotland Uk: Association for Computing Machinery, 2019:1‐15. [DOI: doi.org/10.1145/3290605.3300329]

[CD011942-bib-0144] Johnson EM, Howard C. A library mobile device deployment to enhance the medical student experience in a rural longitudinal integrated clerkship. Journal of the Medical Library Association 2019;107(1):30‐42. 10.5195/jmla.2019.442 PMC6300226 30598646

[CD011942-bib-0145] Kapoor S, O'Grady M, Gilmer E, Neighbors CJ, Kwon N, Conigliaro J, et al. Point‐of‐care mobile application to guide health professionals in conducting substance use screening and intervention: A mixed‐methods user experience study. Journal of General Internal Medicine 2019;34(2 Suppl):S315‐6.

[CD011942-bib-0146] Kaunda‐Khangamwa BN, Steinhardt LC, Rowe AK, Gumbo A, Moyo D, Nsona H, et al. The effect of mobile phone text message reminders on health workers' adherence to case management guidelines for malaria and other diseases in Malawi: lessons from qualitative data from a cluster‐randomized trial. Malaria Journal 2018;17(481):1‐13. 10.1186/s12936-018-2629-2 PMC6299948 30567603

[CD011942-bib-0147] Kawakyu N, Nduati R, Munguambe K, Coutinho J, Mburu N, DeCastro G, et al. Development and implementation of a mobile phone‐based prevention of mother‐to‐child transmission of HIV cascade analysis tool: Usability and feasibility testing in Kenya and Mozambique. Journal of Medical Internet Research 2019;7(5):e13963. 10.2196/13963 PMC6535976 31094351

[CD011942-bib-0148] Klocek A, Smahelova M, Knapova L, Elavsky S. GPs' perspectives on eHealth use in the Czech Republic: a cross‐sectional mixed‐design survey study. BJGP Open 2019;3(3):1‐12. 10.3399/bjgpopen19X101655 PMC6970584 31344683

[CD011942-bib-0149] Kolltveit BC, Thorne S, Graue M, Gjengedal E, Iversen MM, Kirkevold M. Telemedicine follow‐up facilitates more comprehensive diabetes foot ulcer care: A qualitative study in home‐based and specialist health care. Journal of Clinical Nursing 2018;27(5‐6):e1134‐45. 10.1111/jocn.14193 29193527

[CD011942-bib-0150] Kurumop SF, Bullen C, Whittaker R, Betuela I, Hetzel MW, Pulford J. Improving health worker adherence to malaria treatment guidelines in Papua New Guinea: feasibility and acceptability of a text message reminder service. PLOS ONE 2013;8(10):e76578. 10.1371/journal.pone.0076578 PMC3792049 24116122

[CD011942-bib-0151] Laar AS, Bekyieriya E, Isang S, Baguune B. Assessment of mobile health technology for maternal and child health services in rural upper west region of Ghana. Public Health 2019;168:1‐8. 10.1016/j.puhe.2018.11.014 30660898

[CD011942-bib-0152] Larsen‐Cooper E, Bancroft E, O’Toole M, Jezman Z. Where there is no phone: the benefits and limitations of using intermediaries to extend the reach of mHealth to individuals without personal phones in Malawi. African Population Studies 2015;29(1):1628‐42.

[CD011942-bib-0153] Lemay NV, Sullivan T, Jumbe B, Perry CP. Reaching remote health workers in Malawi: baseline assessment of a pilot mHealth intervention. Journal of Health Communication 2012;17(Suppl 1):105‐17. 10.1080/10810730.2011.649106 22548604

[CD011942-bib-0154] Lindberg M, Rosborg S, Ramukumba MM, HÄGglund M. Adapting mHealth to workflow ‐ a case study in South Africa. Studies in Health Technology and Informatics 2019;265:48‐53. 10.3233/SHTI190136 31431576

[CD011942-bib-0155] MacDonald ME, Diallo GS. Socio‐cultural contextual factors that contribute to the uptake of a mobile health intervention to enhance maternal health care in rural Senegal. Reproductive Health 2019;16(1):1‐12. 10.1186/s12978-019-0800-z PMC6739977 31511028

[CD011942-bib-0156] Maraba N, Hoffmann CJ, Chihota VN, Chang LW, Ismail N, Candy S, et al. Using mHealth to improve tuberculosis case identification and treatment initiation in South Africa: Results from a pilot study. PLOS ONE 2018;13(7):e0199687. 10.1371/journal.pone.0199687 PMC6029757 29969486

[CD011942-bib-0157] Martinez‐Brockman JL, Harari N, Goeschel L, Bozzi V, Perez‐Escamilla R. A qualitative analysis of text message conversations in a breastfeeding peer counselling intervention. Maternal and Child Nutrition 2019;11:e12904. 10.1111/mcn.12904 PMC7083457 31823503

[CD011942-bib-0158] Mather C, Cummings E, Gale F. Nurses as stakeholders in the adoption of mobile technology in Australian health care environments: interview study. Journal of Medical Internet Research 2019;2(1):e14279. 10.2196/14279 PMC8279446 34345771

[CD011942-bib-0159] McBride B, O’Neil JD, Hue TT, Eni R, Nguyen CV, Nguyen LT. Improving health equity for ethnic minority women in Thai Nguyen, Vietnam: qualitative results from an mHealth intervention targeting maternal and infant health service access. Journal of Public Health 2018;40(Suppl 2):ii32‐41. 10.1093/pubmed/fdy165 PMC6294030 30252117

[CD011942-bib-0160] McHenry MS, Apondi E, McAteer CI, Nyandiko WM, Fischer LJ, Ombitsa AR, et al. Tablet‐based disclosure counselling for HIV‐infected children, adolescents, and their caregivers: a pilot study. African Journal of AIDS Research 2018;17(3):249‐58. 10.2989/16085906.2018.1509101 PMC6376488 30319030

[CD011942-bib-0161] Mengesha W, Steege R, Kea AZ, Theobald S, Datiko DG. Can mHealth improve timeliness and quality of health data collected and used by health extension workers in rural Southern Ethiopia?. Journal of Public Health 2018;40(Supplement 2):ii74‐86. 10.1093/pubmed/fdy200 PMC6294041 30551131

[CD011942-bib-0162] Musabyimana A, Ruton H. Assessing the perspectives of users and beneficiaries of a community health worker mHealth tracking system for mothers and children in Rwanda. PLOS One 2018;13(6):e0198725. 10.1371/journal.pone.0198725 PMC5991741 29879186

[CD011942-bib-0163] Nes AAG, Dulmen S van, Brembo EA, Eide H. An mHealth intervention for persons with diabetes Type 2 based on acceptance and commitment therapy principles: examining treatment fidelity. Journal of Medical Internet Research mHealth and uHealth 2018;6(7):e151. 10.2196/mhealth.9942 PMC6053615 29970357

[CD011942-bib-0164] Ngor P, White LJ, Chalk J, Lubell Y, Favede C, Cheah PY, et al. Smartphones for community health in rural Cambodia: A feasibility study. Wellcome Open Research 2018;3(69):1‐13. 10.12688/wellcomeopenres.13751.1 PMC6069733 30116791

[CD011942-bib-0165] Nichols M, Singh A, Sarfo FS, Treiber F, Tagge R, Jenkins C, et al. Post‐intervention qualitative assessment of mobile health technology tomanage hypertension among Ghanaian stroke survivors. Journal of Neurological Science 2019;406:1‐9. 10.1016/j.jns.2019.116462 PMC7653548 31610382

[CD011942-bib-0166] Orchard J, Li J, Gallagher R, Freedman B, Lowres N, Neubeck L. Uptake of a primary care atrial fibrillation screening program (AF‐SMART): a realist evaluation of implementation in metropolitan and rural general practice. BMC Family Practice 2019;20(170):1‐13. 10.1186/s12875-019-1058-9 PMC6896363 31810441

[CD011942-bib-0167] Palazuelos D, Diallo AB, Palazuelos L, Carlile N, Payne JD, Franke MF. User perceptions of an mhealth medicine dosing tool for community health workers. Journal of Medical Internet Research 2013;1(1):1‐15. 10.2196/mhealth.2459 PMC4114471 25100670

[CD011942-bib-0168] Patel SJ, Subbiah S, Jones R, Muigai F, Rothschild CW, Omwodo L, et al. Providing support to pregnant women and new mothers through moderated WhatsApp groups: a feasibility study. Mhealth 2018;4(14):1‐8. 10.21037/mhealth.2018.04.05 PMC5994467 29963559

[CD011942-bib-0169] Pimmer C, Mhango S, Mzumara A, Mbvundula F. Mobile instant messaging for rural community health workers: a case from Malawi. Global Health Action 2017;10(1):1‐11. 10.1080/16549716.2017.1368236 PMC5645652 28914165

[CD011942-bib-0170] Pimmer C, Mbvundula F. One message, many voices: mobile audio counselling in health education. Journal of Health Care for the Poor and Underserved 2018;29(1):463‐80. 10.1353/hpu.2018.0031 29503312

[CD011942-bib-0171] Ramukumba MM, Hagglund M. "i feel like a nurse and my clients learn more": mHealth, capacity building and empowerment in community based care. Studies in Health Technology and Informatics 2019;265:195‐200. 10.3233/SHTI190163 31431598

[CD011942-bib-0172] Rassi C, Gore‐Langton GR, Walimbwa BG, Strachan CE, King R, Basharat S, et al. Improving health worker performance through text messaging: a mixed‐methods evaluation of a pilot intervention designed to increase coverage of intermittent preventive treatment of malaria in pregnancy in West Nile, Uganda. Plos One 2018;13(9):e0203554. 10.1371/journal.pone.0203554 PMC6126848 30188956

[CD011942-bib-0173] Rose J, Glazebrook C, Wharrad H, Siriwardena AN, Swift JA, Nathan D, et al. Proactive assessment of obesity risk during infancy (ProAsk): a qualitative study of parents' and professionals' perspectives on an mHealth intervention. BMC Public Health 2019;19(294):1‐10. 10.1186/s12889-019-6616-5 PMC6417230 30866879

[CD011942-bib-0174] Saleem JJ, Savoy A, Etherton G, Herout J. Investigating the need for clinicians to use tablet computers with a newly envisioned electronic health record. International Journal of Medical Informatics 2018;110:25‐30. 10.1016/j.ijmedinf.2017.11.013 29331252

[CD011942-bib-0175] Stålberg A, Sandberg A, Söderbäck M. Child‐centred care – health professionals' perceptions of what aspects are meaningful when using interactive technology as a facilitator in healthcare situations. Journal of Pediatric Nursing 2018;43:e10‐7. 10.1016/j.pedn.2018.07.006 30056996

[CD011942-bib-0176] Steege R, Waldman L, Datiko DG, Kea AZ, Taegtmeyer M, Theobald S. 'The phone is my boss and my helper' – a gender analysis of an mHealth intervention with health extension workers in Southern Ethiopia. Journal of Public Health 2018;40(Suppl 2):ii16‐31. 10.1093/pubmed/fdy199 PMC6294039 30551130

[CD011942-bib-0177] Terio M, Eriksson G, Kamwesiga JT, Guidetti S. What's in it for me? A process evaluation of the implementation of a mobile phone‐supported intervention after stroke in Uganda. BMC Public Health 2019;19(562):1‐13. 10.1186/s12889-019-6849-3 PMC6518972 31088411

[CD011942-bib-0178] Thobias J, Kiwanuka A. Design and implementation of an m‐health data model for improving health information access for reproductive and child health services in low resource settings using a participatory action research approach. BMC Medical Informatics and Decision Making 2018;18(45):1‐10. 10.1186/s12911-018-0622-x PMC6019504 29941008

[CD011942-bib-0179] Thomsen CF, Barrie AM, Boas IM, Lund S, Sorensen BL, Oljira FG, et al. Health workers' experiences with the safe delivery app in West Wollega Zone, Ethiopia: a qualitative study. Reproductive Health 2019;16(50):1‐11. 10.1186/s12978-019-0725-6 PMC6506934 31072399

[CD011942-bib-0180] Vamos CA, Griner SB, Kirchharr C, Green SM, DeBate R, Daley EM, et al. The development of a theory‐based eHealth app prototype to promote oral health during prenatal care visits. Translational Behavioral Medicine 2019;9(6):1100‐11. 10.1093/tbm/ibz047 PMC6875649 31009536

[CD011942-bib-0181] Vasalampi A. Adoption and use of a mobile system at home care. Studies in Health Technology & Informatics 2017;242:1042‐6. 28873926

[CD011942-bib-0182] Vélez O, Okyere PB, Kanter AS, Bakken S. A usability study of a mobile health application for rural Ghanaian midwives. Journal of Midwifery and Women's Health 2014;59(2):184‐91. 10.1111/jmwh.12071 PMC3976680 24400748

[CD011942-bib-0183] Venables E, Ndlovu Z, Munyaradzi D, Martinez‐Perez G, Mbofana E, Nyika P, et al. Patient and health‐care worker experiences of an HIV viral load intervention using SMS: A qualitative study. PLoS ONE 2019;14(4):e0215236. 10.1371/journal.pone.0215236 PMC6459516 30973925

[CD011942-bib-0184] Verwey R, Weegen S van der, Spreeuwenberg M, Tange H, Weijden T van der, Witte L de. A pilot study of a tool to stimulate physical activity in patients with COPD or Type 2 diabetes in primary care. Journal of Telemedicine and Telecare 2014;20(1):29‐34. 10.1177/1357633X13519057 24414397

[CD011942-bib-0185] Vroom FB. Feasibility of mobile Health for Treatment Coverage Reporting: Lymphatic Filariasis control Programme in Ghana (PhD thesis). Accra (Ghana): University of Ghana, 2017.

[CD011942-bib-0186] Ware P, Ross HJ, Cafazzo JA, Laporte A, Gordon K, Seto E. User‐centered adaptation of an existing heart failure telemonitoring program to ensure sustainability and scalability: qualitative study. Journal of Medical and Internet Research Cardio 2018;2(2):e11466. 10.2196/11466 PMC6857927 31758774

[CD011942-bib-0187] Ware P, Ross HJ, Cafazzo JA, Laporte A, Gordon K, Seto E. Evaluating the implementation of a mobile phone‐based telemonitoring program: longitudinal study guided by the consolidated framework for implementation research. Journal of Medical Internet Research mHealth and uHealth 2018;6(7):e10768. 10.2196/10768 PMC6092591 30064970

[CD011942-bib-0188] Watson AH, Sabumei G, Mola G, Iedema R. Maternal health phone line: saving women in Papua New Guinea. Journal of Personalized Medicine 2015;5(2):120‐39. 10.3390/jpm5020120 PMC4493491 25923199

[CD011942-bib-0189] Webb MJ, Wadley G, Sanci LA. Experiences of general practitioners and practice support staff using a health and lifestyle screening app in primary health care: implementation case study. Journal of Medical Internet Research mHealth and uHealth 2018;6(4):e105. 10.2196/mhealth.8778 PMC5941099 29691209

[CD011942-bib-0190] White EB, Meyer AJ, Ggita JM, Babirye D, Mark D, Ayakaka I, et al. Feasibility, acceptability, and adoption of digital fingerprinting during contact investigation for tuberculosis in Kampala, Uganda: a parallel‐convergent mixed‐methods analysis. Journal of Medical Internet Research 2018;20(11):e11541. 10.2196/11541 PMC6265600 30442637

[CD011942-bib-0191] White P, Gilworth G, Lewin S, Hogg L, Tuffnell R, Taylor SJ, et al. Improving uptake and completion of pulmonary rehabilitation in COPD with lay health workers: Feasibility of a clinical trial. International Journal of Chronic Obstructive Pulmonary Disease 2019;14:631‐43. 10.2147/COPD.S188731 PMC6419591 30880952

[CD011942-bib-0192] Xiao M, Lei X, Zhang F, Sun Z, Harris VC, Tang X, et al. Home blood pressure monitoring by a mobile‐based model in Chongqing, China: a feasibility study. International Journal of Environmental Research and Public Health 2019;16(18):1‐13. 10.3390/ijerph16183325 PMC6765873 31509950

[CD011942-bib-0193] Zeleke AA, Worku AG, Demissie A, Otto‐Sobotka F, Wilken M, Lipprandt M, et al. Evaluation of electronic and paper‐pen data capturing tools for data quality in a public health survey in a health and demographic surveillance site, Ethiopia: randomized controlled crossover health care information technology evaluation. Journal of Medical Internet Research mHealth and uHealth 2019;7(2):e10995. 10.2196/10995 PMC6388101 30741642

[CD011942-bib-0194] Zhang J, Joshi R, Sun J, Rosenthal SR, Tong M, Li C, et al. A feasibility study on using smartphones to conduct short‐version verbal autopsies in rural China. Population Health Metrics 2016;14(31):1‐13. 10.1186/s12963-016-0100-6 PMC4992268 27547126

[CD011942-bib-0195] Agarwal S, LeFevre AE, Lee J, L’Engle K, Mehl G, Sinha C, et al. Guidelines for reporting of health interventions using mobile phones: mobile health (mHealth) evidence reporting and assessment (mERA) checklist. BMJ 2016;352:i1174. 10.1136/bmj.i1174 26988021

[CD011942-bib-0196] Agarwal S, Tamrat T, Fønhus MS, Henschke N, Bergman H, Mehl GL, et al. Tracking health commodity inventory and notifying stock levels via mobile devices. Cochrane Database of Systematic Reviews 2018, Issue 1. [DOI: 10.1002/14651858.CD012907] PMC8094928 33539585

[CD011942-bib-0197] Agarwal S, Vasudevan L, Tamrat T, Glenton C, Lewin S, Bergman H, et al. Digital tracking, provider decision support systems, and targeted client communication via mobile devices to improve primary health care. Cochrane Database of Systematic Reviews 2018, Issue 1. [DOI: 10.1002/14651858.CD012925] PMC11975193 40193137

[CD011942-bib-0198] Agarwal S, Tamrat T, Glenton C, Lewin S, Henschke N, Maayan N, et al. Decision‐support tools via mobile devices to improve quality of care in primary healthcare settings. Cochrane Database of Systematic Reviews 2018, Issue 2. [DOI: 10.1002/14651858.CD012944] PMC8406991 34314020

[CD011942-bib-0199] Akter S, Ray P. mHealth ‐ an ultimate platform to serve the unserved. Yearbook of Medical Informatics 2010;2010:94‐100. 20938579

[CD011942-bib-0200] Ames HM, Glenton C, Lewin S, Tamrat T, Akama E, Leon N. Clients' perceptions and experiences of targeted digital communication accessible via mobile devices for reproductive, maternal, newborn, child, and adolescent health: a qualitative evidence synthesis. Cochrane Database of Systematic Reviews 2019, Issue 10. [DOI: 10.1002/14651858.CD013447] PMC6791116 31608981

[CD011942-bib-0201] Anderson‐Lewis C, Darville G, Mercado RE, Howell S, Maggio S Di. mHealth technology use and implications in historically underserved and minority populations in the United States: systematic literature review. Journal of Medical Internet Research mHealth and uHealth 2018;6(6):e128. 10.2196/mhealth.8383 PMC6028762 29914860

[CD011942-bib-0202] Aranda‐Jan CB, Mohutsiwa‐Dibe N, Loukanova S. Systematic review on what works, what does not work and why of implementation of mobile health (mHealth) projects in Africa. BMC Public Health 2014;14(188):1‐15. 10.1186/1471-2458-14-188 PMC3942265 24555733

[CD011942-bib-0203] Atherton H, Sawmynaden P, Meyer B, Car J. Email for the coordination of healthcare appointments and attendance reminders. Cochrane Database of Systematic Reviews 2012, Issue 8. [DOI: 10.1002/14651858.CD007981.pub2] PMC11627138 22895971

[CD011942-bib-0204] Atkins S, Lewin S, Smith H, Engel M, Fretheim A, Volmink J. Conducting a meta‐ethnography of qualitative literature: lessons learnt. BMC Medical Research Methodology 2008;8(21):1‐10. 10.1186/1471-2288-8-21 PMC2374791 18416812

[CD011942-bib-0205] Awofeso N. What is the difference between ’primary care’and ’primary healthcare’?. Quality in Primary Care 2004;12:93‐4.

[CD011942-bib-0206] Barron P, Peter J, LeFevre AE, Sebidi J, Bekker M, Allen R, et al. Mobile health messaging service and helpdesk for South African mothers (MomConnect): history, successes and challenges. BMJ Global Health 2018;3(Suppl 2):e000559. 10.1136/bmjgh-2017-000559 PMC5922496 29713503

[CD011942-bib-0207] Blaya JA, Fraser HS, Holt B. E‐health technologies show promise in developing countries. Health Affairs 2010;29(2):244‐51. 10.1377/hlthaff.2009.0894 20348068

[CD011942-bib-0208] Boonstra TW, Nicholas J, Wong QJ, Shaw F, Townsend S, Christensen H. Using mobile phone sensor technology for mental health research: integrated analysis to identify hidden challenges and potential solutions. Journal of Medical Internet Research 2018;20(7):e10131. 10.2196/10131 PMC6090171 30061092

[CD011942-bib-0209] Braun R, Catalani C, Wimbush J, Israelski D. Community health workers and mobile technology: a systematic review of the literature. PLoS ONE 2013;8(6):e65772. 10.1371/journal.pone.0065772 PMC3680423 23776544

[CD011942-bib-0210] Catalani C, Philbrick W, Fraser H, Mechael P, Israelski DM. MHealth for HIV treatment & prevention: a systematic review of the literature. The Open AIDS Journal 2013;7:17‐41. 10.2174/1874613620130812003 PMC3795408 24133558

[CD011942-bib-0211] Chang LW, Njie‐Carr V, Kalenge S, Kelly JF, Bollinger RC, Alamo‐Talisuna S. Perceptions and acceptability of mHealth interventions for improving patient care at a community‐based HIV/AIDS clinic in Uganda: a mixed methods study. AIDS Care 2013;25(7):874‐80. 10.1080/09540121.2013.774315 PMC3688650 23452084

[CD011942-bib-0212] Chib A, Velthoven MH Van, Car J. mHealth adoption in low‐resource environments: a review of the use of mobile healthcare in developing countries. Journal of Health Communication 2015;20(1):4‐34. 10.1080/10810730.2013.864735 24673171

[CD011942-bib-0213] Veritas Health Innovation. Covidence. Version accessed 16 March 2020. Melbourne, Australia: Veritas Health Innovation.

[CD011942-bib-0214] Dunn EE, Gainforth HL, Robertson‐Wilson JE. Behavior change techniques in mobile applications for sedentary behavior. Digital Health 2018;4:1‐8. 10.1177/2055207618785798 PMC6043935 31463076

[CD011942-bib-0215] Gagnon MP, Legare F, Labrecque M, Fremont P, Pluye P, Gagnon J, et al. Interventions for promoting information and communication technologies adoption in healthcare professionals. Cochrane Database of Systematic Reviews 2009, Issue 1. [DOI: 10.1002/14651858.CD006093.pub2] PMC3973635 19160265

[CD011942-bib-0216] Gilson L. Health Policy and Systems Research: a methodology reader. 1st Edition. Geneva, Switzerland: WHO Document Production Services, 2012.

[CD011942-bib-0217] Glenton C, Colvin CJ, Carlsen B, Swartz A, Lewin S, Noyes J, et al. Barriers and facilitators to the implementation of lay health worker programmes to improve access to maternal and child health: qualitative evidence synthesis. Cochrane Database of Systematic Reviews 2013, Issue 10. [DOI: 10.1002/14651858.CD010414.pub2] PMC6396344 24101553

[CD011942-bib-0218] Glenton C, Carlsen B, Lewin S, Munthe‐Kaas H, Colvin CJ, Tunçalp Ö, et al. Applying GRADE‐CERQual to qualitative evidence synthesis findings—paper 5: how to assess adequacy of data. Implementation Science 2018;13(Suppl 1):1‐8. 10.1186/s13012-017-0692-7 PMC5791045 29384077

[CD011942-bib-0219] Global Health Watch. Primary health care: a review and critical appraisal of its revitalization. www.ghwatch.org/sites/www.ghwatch.org/files/B1_0.pdf (accessed prior to 23 March 2020).

[CD011942-bib-0220] Gonçalves‐Bradley DC, Buckley BS, Fønhus MS, Glenton C, Henschke N, Lewin S, et al. Mobile‐based technologies to support client to healthcare provider communication and management of care. Cochrane Database of Systematic Reviews 2018, Issue 1. [DOI: 10.1002/14651858.CD012928] PMC7437392 32813281

[CD011942-bib-0221] Gonçalves‐Bradley D, Buckley BS, Fønhus MS, Glenton C, Henschke N, Lewin S, et al. Mobile‐based technologies to support healthcare provider to healthcare provider communication and management of care. Cochrane Database of Systematic Reviews 2018, Issue 1. [DOI: 10.1002/14651858.CD012927] PMC7437392 32813281

[CD011942-bib-0222] Grimsbø GH, Engelsrud GH, Ruland CM, Finset A. Cancer patients’ experiences of using an interactive health communication application (IHCA). International Journal of Qualitative Studies on Health and Well‐being 2012;7:1‐14. 10.3402/qhw.v7i0.15511 PMC3349955 22582085

[CD011942-bib-0223] Gurol‐Urganci I, Jongh T De, Vodopivec‐Jamsek V, Atun R, Car J. Mobile phone messaging reminders for attendance at healthcare appointments. Cochrane Database of Systematic Reviews 2013, Issue 12. [DOI: 10.1002/14651858.CD007458.pub3] PMC6485985 24310741

[CD011942-bib-0224] Hall CS, Fottrell E, Wilkinson S, Byass P. Assessing the impact of mHealth interventions in low‐ and middle‐income countries – what has been shown to work?. Global Health Action 2014;7:1‐12. 10.3402/gha.v7.25606 PMC4216389 25361730

[CD011942-bib-0225] Huang F, Blaschke S, Lucas H. Beyond pilotitis: taking digital health interventions to the national level in China and Uganda. Globalization and Health 2017;13(49):1‐11. 10.1186/s12992-017-0275-z PMC5535287 28756767

[CD011942-bib-0226] Hurt K, Walker RJ, Campbell JA, Egede LE. mHealth interventions in low and middle‐income countries: a systematic review. Global Journal of Health Science 2016;8(9):183‐93. 10.5539/gjhs.v8n9p183 PMC5064069 27157176

[CD011942-bib-0227] Konttila J, Siira H, Kyngäs H, Lahtinen M, Elo S, Kääriäinen M, et al. Healthcare professionals’ competence in digitalisation: a systematic review. Journal of Clinical Nursing 2019;28(5‐6):745‐61. 10.1111/jocn.14710 30376199

[CD011942-bib-0228] Labrique AB, Kirk GD, Westergaard RP, Merritt MW. Ethical issues in mHealth research involving persons living with HIV/AIDS and substance abuse. AIDS Research and Treatment 2013;2013:189645. 10.1155/2013/189645 PMC3792525 24171110

[CD011942-bib-0229] Labrique A, Vasudevan L, Chang LW, Mehl G. H_pe for mHealth: More “y” or “o” on the horizon?. International Journal of Medical Informatics 2013;82(5):467‐9. 10.1016/j.ijmedinf.2012.11.016 PMC3849805 23279850

[CD011942-bib-0230] Labrique AB, Vasudevan L, Kochi E, Fabricant R, Mehl G. mHealth innovations as health system strengthening tools: 12 common applications and a visual framework. Global Health: Science Practice 2013;1(2):160‐71. 10.9745/GHSP-D-13-00031 PMC4168567 25276529

[CD011942-bib-0231] Langlois E, Daniels K, Akl E. Evidence Synthesis for Health Policy and Systems: a methods guide. Geneva, Switzerland: World Health Organization, 2018. 33877749

[CD011942-bib-0232] Lee SH, Nurmatov UB, Nwaru BI, Mukherjee M, Grant L, Pagliari C. Effectiveness of mHealth interventions for maternal, newborn and child health in low‐ and middle‐income countries: systematic review and meta‐analysis. Journal of Global Health 2016;6(1):1‐17. 10.7189/jogh.06.010401 PMC4643860 26649177

[CD011942-bib-0233] Leon N, Schneider H, Daviaud E. Applying a framework for assessing the health system challenges to scaling up mHealth in South Africa. BMC Medical Informatics and Decision Making 2012;12(123):1‐12. 10.1186/1472-6947-12-123 PMC3534437 23126370

[CD011942-bib-0234] Lewin SA, Dick J, Pond P, Zwarenstein M, Aja G, Wyk B van, et al. Lay health workers in primary and community health care. Cochrane Database of Systematic Reviews 2005, Issue 3. [DOI: 10.1002/14651858.CD004015.pub2] 15674924

[CD011942-bib-0235] Lewin S, Booth A, Glenton C, Munthe‐Kaas H, Rashidian A, Wainwright M, et al. Applying GRADE‐CERQual to qualitative evidence synthesis findings: introduction to the series. Implementation Science 2018;13(Suppl 1):1‐13. 10.1186/s13012-017-0688-3 PMC5791040 29384079

[CD011942-bib-0236] Marcolino MS, Oliveira JAQ. The impact of mHealth interventions: systematic review of systematic reviews. Journal of Medical Internet Research mHealth and uHealth 2018;6(1):e23. 10.2196/mhealth.8873 PMC5792697 29343463

[CD011942-bib-0237] Mbuagbaw L, Medley N, Darzi AJ, Richardson M, Habiba GK, Ongolo‐Zogo P. Health system and community level interventions for improving antenatal care coverage and health outcomes. Cochrane Database of Systematic Reviews 2015, Issue 12. [DOI: 10.1002/14651858.CD010994.pub2] PMC4676908 26621223

[CD011942-bib-0238] McCloud RF, Okechukwu CA, Sorensen G, Viswanath K. Beyond access: barriers to Internet health information seeking among the urban poor. Journal of the American Medical Informatics Association 2016;23(6):1053‐9. 10.1093/jamia/ocv204 PMC5070515 27206459

[CD011942-bib-0239] Mehl G, Labrique A. Prioritizing integrated mHealth strategies for universal health coverage. Science 2014;345(6202):1284‐7. 10.1126/science.1258926 25214614

[CD011942-bib-0240] Muldoon LK, Hogg WE, Levitt M. Primary care (PC) and primary health care (PHC): what is the difference?. Canadian Journal of Public Health/Revue Canadienne de Santé Publique 2006;97(5):409‐11. 10.1007/BF03405354 PMC6976192 17120883

[CD011942-bib-0241] Neupane S, Odendaal W, Friedman I, Jassat W, Schneider H, Doherty T. Comparing a paper based monitoring and evaluation system to a mHealth system to support the national community health worker programme, South Africa: an evaluation. BMC Medical Informatics and Decision Making 2014;14(69):1‐9. 10.1186/1472-6947-14-69 PMC4150556 25106499

[CD011942-bib-0242] Olaniran A, Smith H, Unkels R, Bar‐Zeev S, Broek N van den. Who is a community health worker? A systematic review of definitions. Global Health Action 2017;10(1):1‐13. 10.1080/16549716.2017.1272223 PMC5328349 28222653

[CD011942-bib-0243] Pappas Y, Atherton H, Sawmynaden P, Car J. Email for clinical communication between healthcare professionals. Cochrane Database of Systematic Reviews 2012, Issue 9. [DOI: 10.1002/14651858.CD007979] 22972116

[CD011942-bib-0244] Pawson R, Tilley N. Realistic evaluation bloodlines. American Journal of Evaluation 2001;22:317–24.

[CD011942-bib-0245] Qiang CZ, Yamamichi M, Hausman V, Altman D, Unit IS. Mobile applications for the health sector. Washington: World Bank 2011.

[CD011942-bib-0246] Sandelowski M. Sample size in qualitative research. Research in Nursing & Health 1995;18(2):179‐83. 10.1002/nur.4770180211 7899572

[CD011942-bib-0247] Siedner MJ, Lankowski A, Musinga D, Jackson J, Muzoora C, Hunt PW, et al. Optimizing network connectivity for mobile health technologies in sub‐Saharan Africa. PloS One 2012;7(9):e45643. 10.1371/journal.pone.0045643 PMC3460947 23029155

[CD011942-bib-0248] Thomas J, Harden A. Methods for the thematic synthesis of qualitative research in systematic reviews. BMC Medical Research Methodology 2008;8(45):1‐10. 10.1186/1471-2288-8-45 PMC2478656 18616818

[CD011942-bib-0249] Tomlinson M, Rotheram‐Borus MJ, Swartz L, Tsai AC. Scaling up mHealth: where is the evidence?. PLoS Medicine 2013;10(2):e1001382. 10.1371/journal.pmed.1001382 PMC3570540 23424286

[CD011942-bib-0250] Vasudevan L, Henschke N, Glenton C, Lewin S, Maayan N, Eyers J, et al. Birth and death notification via mobile devices. Cochrane Database of Systematic Reviews 2018, Issue 1. [DOI: 10.1002/14651858.CD012909] PMC8785898 34271590

[CD011942-bib-0251] Vervloet M, Linn AJ, Weert JC van, Bakker DH de, Bouvy ML, Dijk L van. The effectiveness of interventions using electronic reminders to improve adherence to chronic medication: a systematic review of the literature. Journal of the American Medical Informatics Assocation 2012;19(5):696‐704. 10.1136/amiajnl-2011-000748 PMC3422829 22534082

[CD011942-bib-0252] Wang EH, Zhou L, Chen SHK, Hill K, Parmanto B. An mHealth platform for supporting clinical data integration into augmentative and alternative communication service delivery: user‐centered design and usability evaluation. Journal of Medical Internet Research Rehabilitation and Assistive Technologies 2018;5(2):e14. 10.2196/rehab.9009 PMC6081606 30042092

[CD011942-bib-0253] West KL. mHealth: A Comprehensive and Contemporary Look at Emerging Technologies in Mobile Health (Honours thesis). Tennessee (USA): University of Tennessee, 2014.

[CD011942-bib-0254] World Health Organization. mHealth: new horizons for health through mobile technologies: based on the findings of the second Global Survey on eHealth. apps.who.int/iris/bitstream/handle/10665/44607/9789241564250_eng.pdf?sequence=1&isAllowed=y (accessed prior to 24 March 2020).

[CD011942-bib-0255] World Health Organization. Classification of Digital Health Interventions. www.who.int/reproductivehealth/publications/mhealth/classification‐digital‐health‐interventions/en/2018.

[CD011942-bib-0256] WHO Guideline: Recommendations on Digital Interventions for Health System Strengthening. apps.who.int/iris/bitstream/handle/10665/311941/9789241550505‐eng.pdf?ua=1 2019. 31162915

[CD011942-bib-0257] Odendaal WA, Goudge J, Griffiths F, Tomlinson M, Leon N, Daniels K. Healthcare workers' perceptions and experience on using mHealth technologies to deliver primary healthcare services: qualitative evidence synthesis. Cochrane Database of Systematic Reviews 2015, Issue 11. [DOI: 10.1002/14651858.CD011942] PMC4966615 27478408

